# Supplementary material for: Lobule-Related Action Potential Shape- and History-Dependent Current Integration in Purkinje Cells of Adult and Developing Mice
Source: Cells. 2023 Feb 15;12(4):623. doi: 10.3390/cells12040623 (PMC9953991; doi:10.3390/cells12040623)
Supplement: Supplementary file 1 [file cells-12-00623-s001.zip › Supplementary_figures-proofs.pdf]

cells MDPI

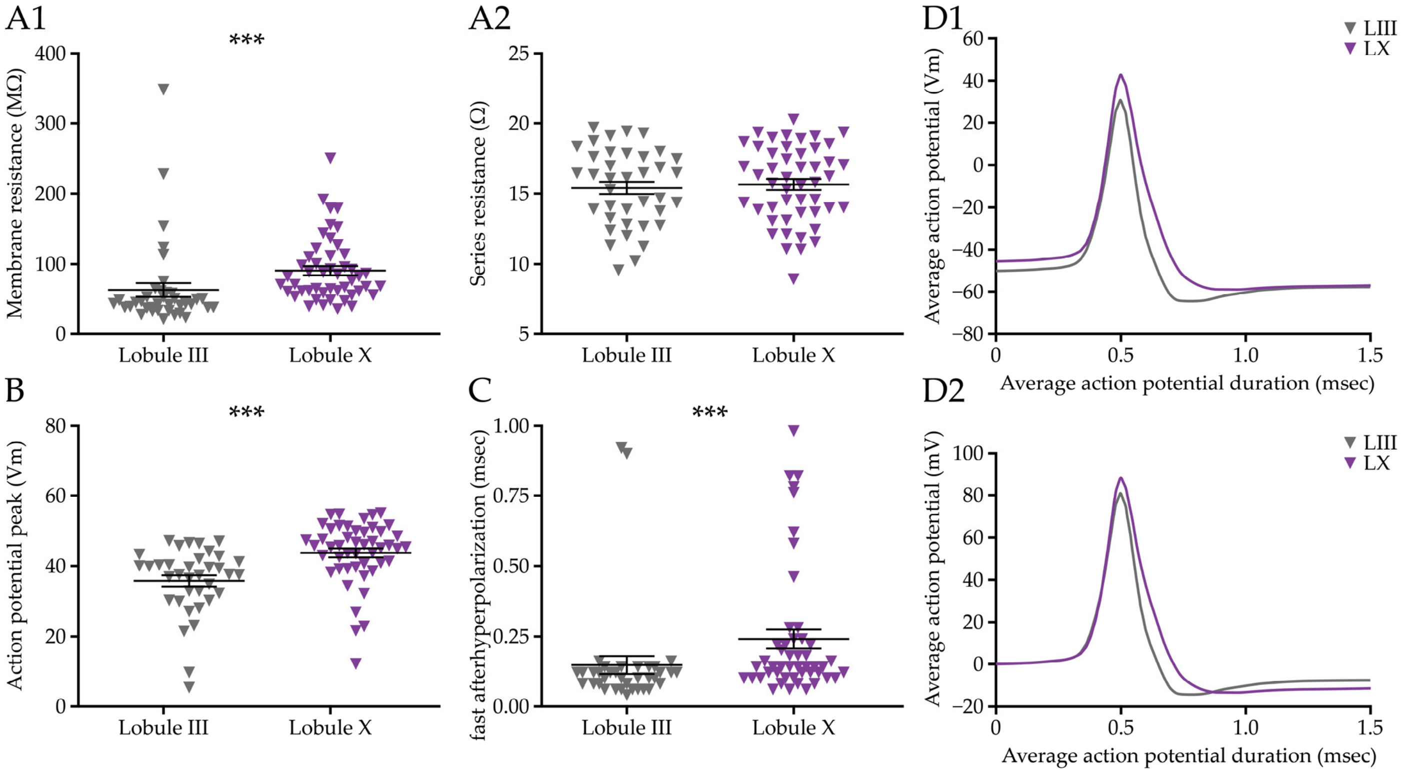

Figure S1. Additional cellular properties and action potential features of different subpopulations. A, Analysis of membrane and series resistance, complementing input resistance analysis in Figure 1B. B, Plot of the spike maximum, the peak membrane potential value of the action potential. C. Analysis of the duration of the fast after hyperpolarization (in msec). D1, Superimposed traces of the average action potential in LIII and LX. D2, The start of the trace from D1 normalized at 0 mV with superimposed traces of the average action potential in LIII and LX. Asterisks (***) denote $p<0.001$. See Table S1 for all statistical data.

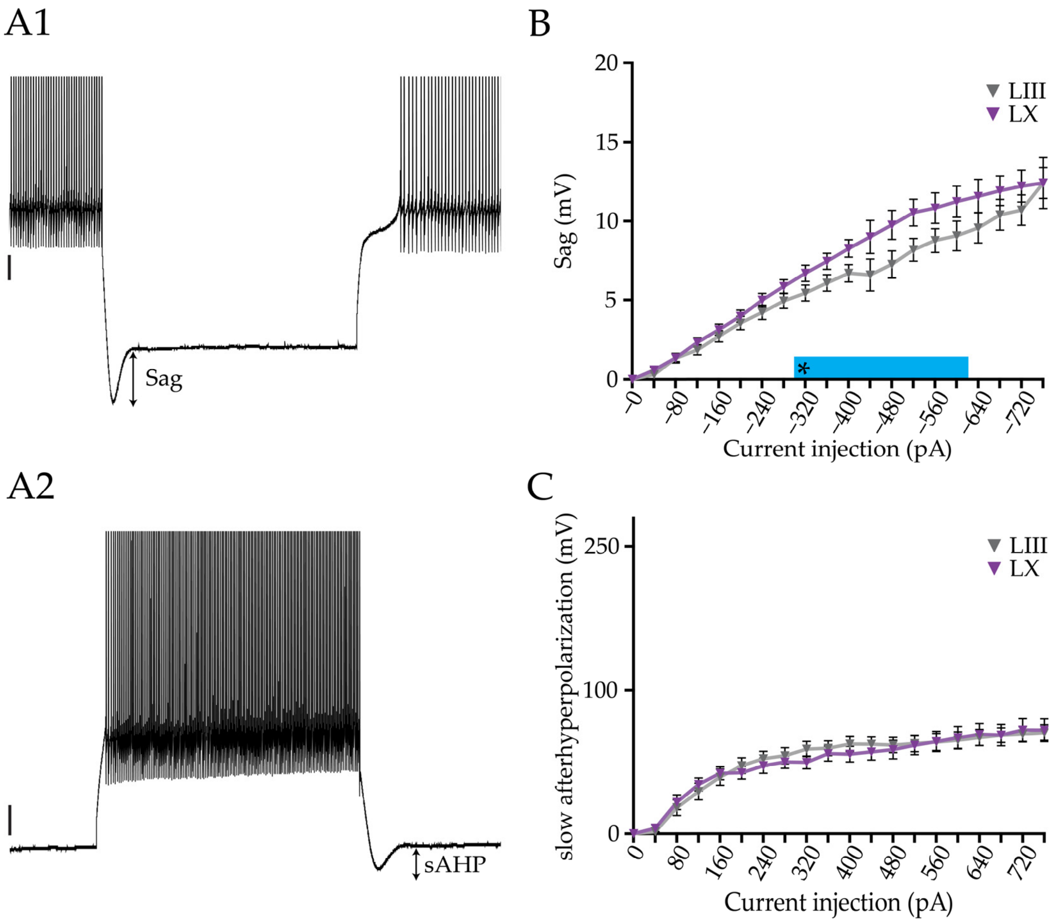

Figure S2. Comparison of voltage sag and slow after hyperpolarization. A, example traces showing the voltage sag following a negative current pulse (top) and the slow after hyperpolarization (bottom). B, Analysis of the voltage sag induced by current injections ranging from 0 to –706 pA for PCs from LIII and LX. C, Plot of slow afterhyperpolarization amplitude against different positive current pulses. Vertical scale bar in A1, 10 mV; A2 10 mV. Asterisk denotes $p<0.05$. See Table S2 for all statistical data.

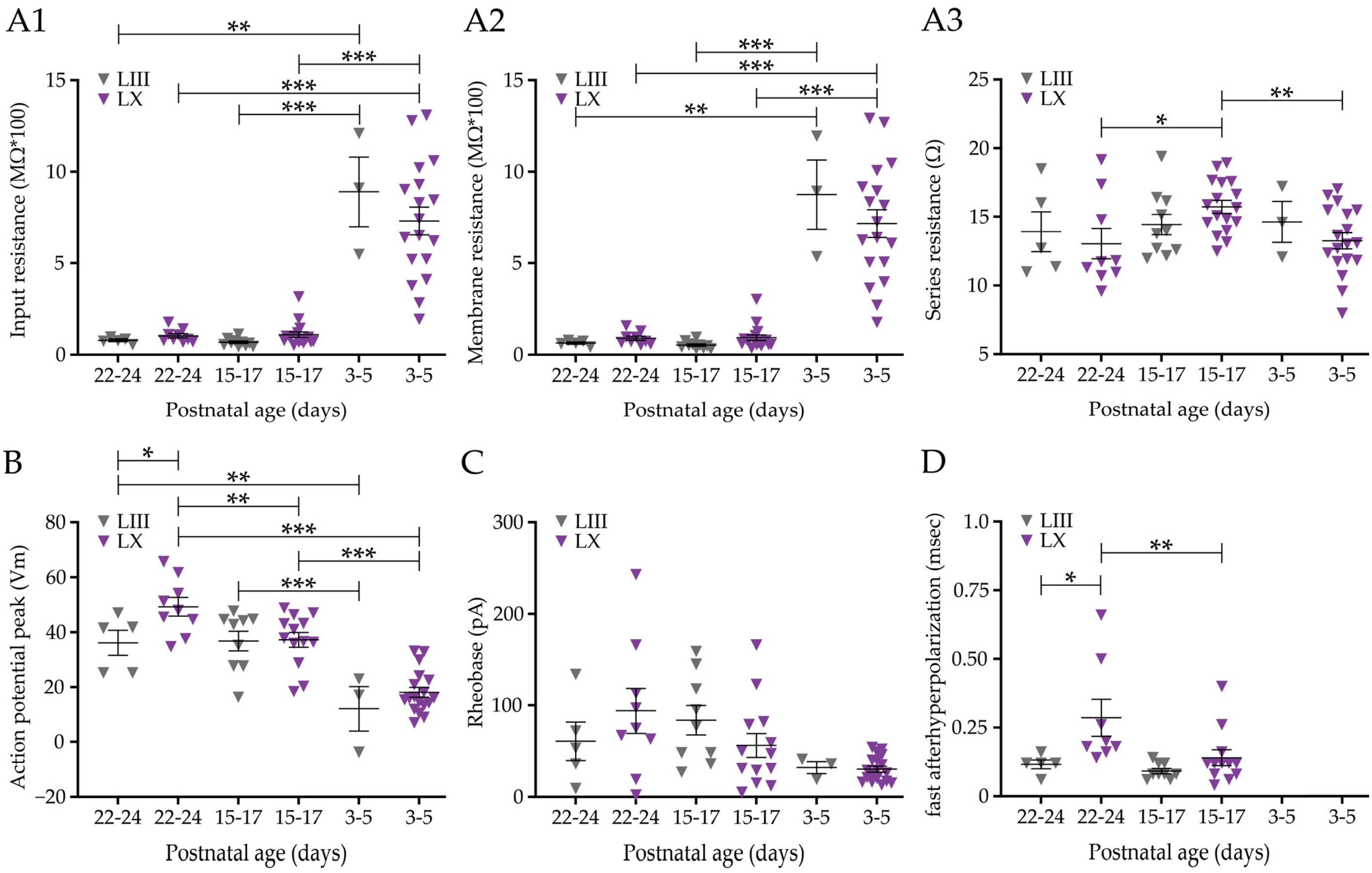

Figure S3. Cellular and action potential properties tracked over development. A, Plots of input resistance (A1), membrane resistance (A2) and series resistance (A3) for P22-24, P15-17 and P3-5 mice, comparing PCs from LIII and LX. B-D, Similar analysis of spike maximum (B), rheobase (C) and the fast hyperpolarization duration (D). Asterisks denote: * for p<0.05, ** for p<0.01 and *** for p<0.001. See Table S4 for all statistical data.

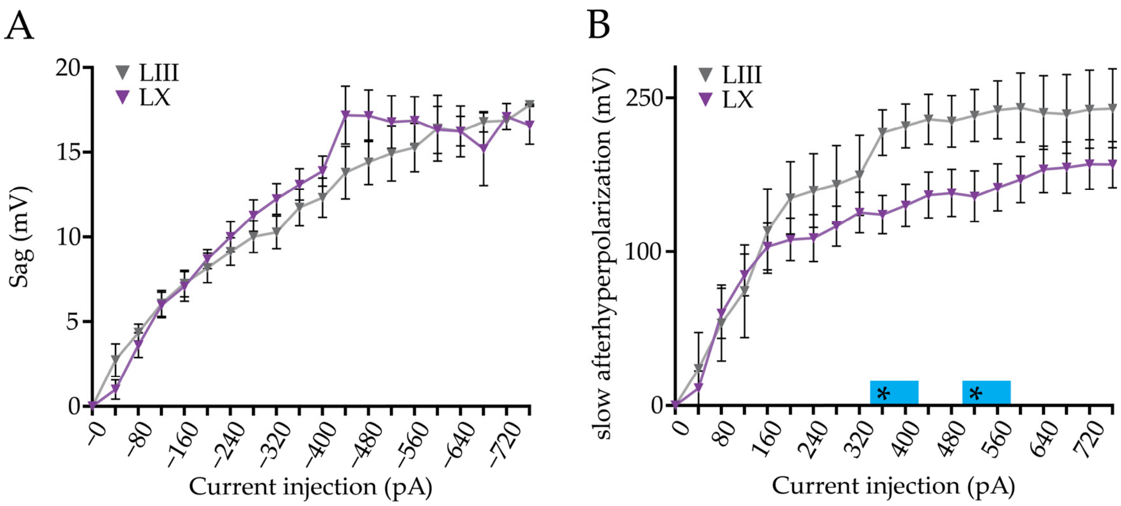

Figure S4. Voltage sag and slow after hyperpolarization in Purkinje cells of juvenile mice. A, Analysis of the voltage sag, comparing PCs from the LIII and LX of P15-17 mice. B, Comparison of the slow after hyperpolarization amplitude in the same PCs. Asterisk denotes $p<0.05$. See Table S5 for all statistical data.

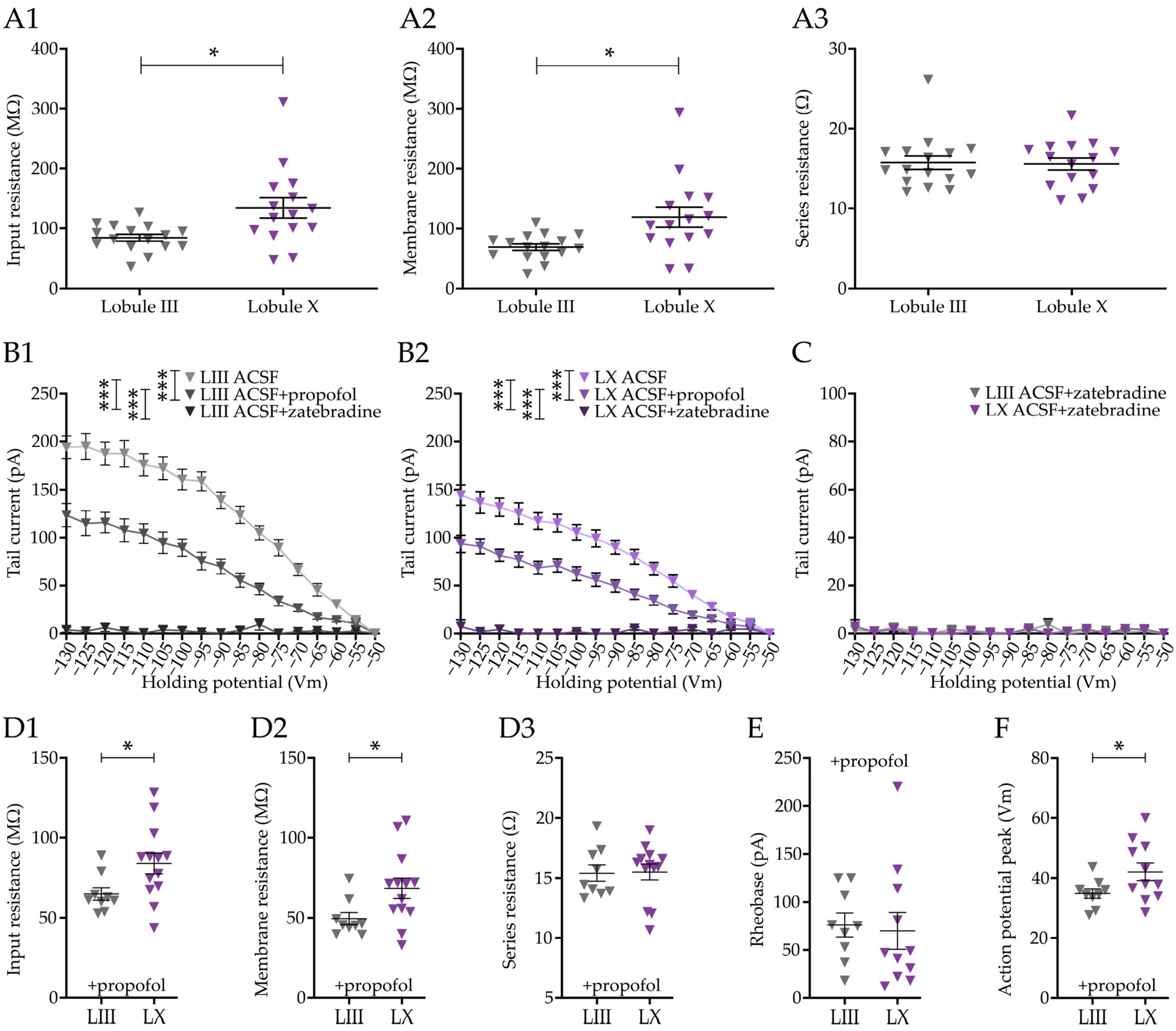

Figure S5. Additional information on HCN currents in subpopulations of cerebellar Purkinje cells. A, Analysis of input (A1), membrane (A2), and series resistance (A3), of the patched PCs used in the tail current experiment with all channel blockers added to the aCSF, except the HCN channel blockers propofol and zatebradine. B, Tail currents analysis comparing the responses in PCs from LIII (B1) and LX (B2) both in >P30 mice in different aCSF solutions. C, Is a comparison between LIII and LX of the tail current after blocking all HCN currents with zatebradine. D, Analysis of input (D1), membrane (D2), and series resistance (D3), of the patched PCs used in the action potential waveform analysis, with propofol added to the aCSF. E-F, analysis of the rheobase and spike maximum of the same cells used in D. See Figure 1 for comparison. Asterisks denote: * for p<0.05, ** for p<0.01 and *** for p<0.001. Table S7 for all statistical data.
