# Supplementary material for: Lobule-Related Action Potential Shape- and History-Dependent Current Integration in Purkinje Cells of Adult and Developing Mice
Source: Cells. 2023 Feb 15;12(4):623. doi: 10.3390/cells12040623 (PMC9953991; doi:10.3390/cells12040623)
Supplement: Supplementary file 1 [file cells-12-00623-s001.zip › Supplementary_tables-proofs.pdf]

cells

Table S1. Statistical data for *in vitro* whole cell patch electrophysiology in the adult mouse (P>30), for action potential analysis. Descriptive values are represented as ± SEM. Abbreviations: LIII = Lobule III, LX = Lobule X, *df* = degrees of freedom, Hz = hertz, pA = picoampere, Ω = Ohm, mV = millivolt, Vm = membrane voltage, msec = millisecond.

Related Figure	Test Applied	Group	*p*-Value	Population-Value	Descriptive Values	Population Size
Figure 1B	Mann–Whitney U test	LIII vs. LX	**<u><0.0001</u>**	(*U*) 379	78.22 ± 9.8 Ω vs. 106.1 ± 6.6 Ω	38 vs. 48 cells
Figure 1C	Mann–Whitney U test	LIII vs. LX	**<u>0.0304</u>**	(*U*) 625	74.25 ± 9.6 pA vs. 47.13 ± 5.5 pA	36 vs. 48 cells
Figure 1D	Mann–Whitney U test	LIII vs. LX	0.3809	(*U*) 750	-43.46 ± 1.0 mV vs. -42.21 ± 1.0 mV	36 vs. 47 cells
Figure 1E	Mann–Whitney U test	LIII vs. LX	**<u>0.0002</u>**	(*U*) 465	88.11 ± 1.8 mV vs. 95.95 ± 1.5 mV	36 vs. 48 cells
Figure 1F1	Mann–Whitney U test	LIII vs. LX	**<u>0.0011</u>**	(*U*) 537	0.145 ± 0.004 msec vs. 0.173 ± 0.006 msec	37 vs. 48 cells
Figure 1F2	Mann–Whitney U test	LIII vs. LX	0.1621	(*U*) 739	0.138 ± 0.007 msec vs. 0.129 ± 0.003 msec	37 vs. 48 cells
Figure 1F3	Welch's *t*-test	LIII vs. LX	**<u><0.0001</u>**	(*t*) 6.805 (*df*) 77	0.118 ± 0.005 msec vs. 0.181 ± 0.008 msec	37 vs. 48 cells
Figure 1G	Unpaired *t*-test	LIII vs. LX	**<u>0.0346</u>**	(*t*) 2.149 (*df*) 82	10.97 ± 0.7 mV vs. 9.11 ± 0.6 mV	37 vs. 48 cells
Figure 1 S1A1	Mann–Whitney U test	LIII vs. LX	**<u><0.0001</u>**	(*U*) 367	62.82 ± 10.0 Ω vs.90.41 ± 6.6 Ω	38 vs. 48 cells
Figure 1 S1A2	Unpaired *t*-test	LIII vs. LX	0.6703	(*t*) 0.427 (*df*) 84	15.40 ± 0.5 Ω vs. 15.66 ± 0.4 Ω	38 vs. 48 cells
Figure 1 S1B	Mann–Whitney U test	LIII vs. LX	**<u><0.0001</u>**	(*U*) 390	35.72 ± 1.6 Vm vs. 43.75 ± 1.3 Vm	36 vs. 48 cells
Figure 1 S1C	Mann–Whitney U test	LIII vs. LX	**<u>0.0005</u>**	(*U*) 490	0.147 ± 0.032 msec vs. 0.240 ± 0.034 msec	36 vs. 48 cells

Table S2. Statistical data for in vitro whole cell patch electrophysiology in the adult mouse (P>30), for negative and positive current step analysis. Descriptive values are represented as ± SEM. Abbreviations: LIII = Lobule III, LX = Lobule X, *df* = degrees of freedom, Hz = hertz, pA = picoampere, mV = millivolt.

Related Figure	Test Applied	Group	*p*-Value	Population-Value	Descriptive Values	Population Size
Figure 2B1	Mixed-effects analysis	-0 pA: LIII vs. LX			0.00 ± 0.0 mV	24 vs. 28 cells
		-40 pA: LIII vs. LX	0.2300	(*t*) 1.201 (*df*) 676	-3.40 ± 0.9 mV vs. -4.86 ± 0.9 mV	24 vs. 28 cells
		-80 pA: LIII vs. LX	0.6317	(*t*) 0.480 (*df*) 676	-11.08 ± 0.8 mV vs. -10.49 ± 0.8 mV	24 vs. 28 cells
		-120 pA: LIII vs. LX	0.1699	(*t*) 1.374 (*df*) 676	-13.26 ± 0.9 mV vs. -14.94 ± 0.7 mV	24 vs. 28 cells
		-160 pA: LIII vs. LX	0.1846	(*t*) 1.328 (*df*) 676	-15.67 ± 0.7 mV vs. -17.29 ± 0.7 mV	24 vs. 28 cells
		-200 pA: LIII vs. LX	0.1561	(*t*) 1.420 (*df*) 676	-17.82 ± 0.6 mV vs. -19.59 ± 0.7 mV	24 vs. 28 cells
		-240 pA: LIII vs. LX	0.0718	(*t*) 1.803 (*df*) 676	-19.63 ± 0.6 mV vs. -21.83 ± 0.7 mV	24 vs. 28 cells
		-280 pA: LIII vs. LX	**0.0414**	(*t*) 2.044 (*df*) 676	-21.37 ± 0.7 mV vs. -23.87 ± 0.8 mV	24 vs. 28 cells
		-320 pA: LIII vs. LX	**0.0126**	(*t*) 2.502 (*df*) 676	-22.87 ± 0.7 mV vs. -25.92 ± 0.8 mV	24 vs. 28 cells
		-360 pA: LIII vs. LX	**0.0035**	(*t*) 2.929 (*df*) 676	-24.40 ± 0.8 mV vs. -27.97 ± 0.9 mV	24 vs. 28 cells
		-400 pA: LIII vs. LX	**0.0015**	(*t*) 3.196 (*df*) 676	-26.04 ± 0.8 mV vs. -29.94 ± 1.0 mV	24 vs. 28 cells
		-440 pA: LIII vs. LX	**0.0022**	(*t*) 3.078 (*df*) 676	-25.27 ± 1.2 mV vs. -33.35 ± 2.4 mV	7 vs. 9 cells
		-480 pA: LIII vs. LX	**0.0008**	(*t*) 3.354 (*df*) 676	-26.56 ± 1.3 mV vs. -35.11 ± 2.3 mV	7 vs. 9 cells
		-520 pA: LIII vs. LX	**0.0005**	(*t*) 3.483 (*df*) 676	-28.58 ± 1.4 mV vs. -37.45 ± -2.5 mV	7 vs. 9 cells
		-560 pA: LIII vs. LX	**0.0001**	(*t*) 3.880 (*df*) 676	-30.02 ± 1.4 mV vs. -39.46 ± 2.7 mV	7 vs. 9 cells
		-600 pA: LIII vs. LX	**>0.0001**	(*t*) 4.223 (*df*) 676	-31.59 ± 1.5 mV vs. -41.61 ± 2.9 mV	7 vs. 9 cells
		-640 pA: LIII vs. LX	**>0.0001**	(*t*) 4.670 (*df*) 676	-33.12 ± 1.6 mV vs. -43.90 ± 3.3 mV	7 vs. 9 cells
		-680 pA: LIII vs. LX	**>0.0001**	(*t*) 4.930 (*df*) 676	-34.43 ± 1.7 mV vs. -45.65 ± 3.3 mV	7 vs. 9 cells
		-720 pA: LIII vs. LX	**>0.0001**	(*t*) 5.405 (*df*) 676	-35.96 ± 1.7 mV vs. -47.98 ± 3.4 mV	7 vs. 9 cells
		-760 pA: LIII vs. LX	**>0.0001**	(*t*) 5.597 (*df*) 676	-37.32 ± 1.7 mV vs. -49.67 ± 3.4 mV	7 vs. 9 cells
Figure 2B2	Mixed-effects analysis	-0 pA: LIII vs. LX	**>0.0001**	(*t*) 5.409 (*df*) 676	45.17 ± 7.2 Hz vs. 29.14 ± 3.5 Hz	24 vs. 28 cells
		-40 pA: LIII vs. LX	**>0.0001**	(*t*) 4.073 (*df*) 676	24.21 ± 6.6 Hz vs. 12.14 ± 3.2 Hz	24 vs. 28 cells
		-80 pA: LIII vs. LX	0.9025	(*t*) 0.123 (*df*) 676	3.21 ± 2.3 Hz vs. 3.57 ± 2.0 Hz	24 vs. 28 cells
		-120 pA: LIII vs. LX	0.6835	(*t*) 0.408 (*df*) 676	1.21 ± 1.0 Hz vs. 0.00 ± 0.0 Hz	24 vs. 28 cells
		-160 pA: LIII vs. LX	>0.9999	(*t*) 0.000 (*df*) 676	0.00 ± 0.0 Hz vs. 0.00 ± 0.0 Hz	24 vs. 28 cells
		-200 pA: LIII vs. LX	>0.9999	(*t*) 0.000 (*df*) 676	0.00 ± 0.0 Hz vs. 0.00 ± 0.0 Hz	24 vs. 28 cells
		-240 pA: LIII vs. LX	>0.9999	(*t*) 0.000 (*df*) 676	0.00 ± 0.0 Hz vs. 0.00 ± 0.0 Hz	24 vs. 28 cells
		-280 pA: LIII vs. LX	>0.9999	(*t*) 0.000 (*df*) 676	0.00 ± 0.0 Hz vs. 0.00 ± 0.0 Hz	24 vs. 28 cells
		-320 pA: LIII vs. LX	>0.9999	(*t*) 0.000 (*df*) 676	0.00 ± 0.0 Hz vs. 0.00 ± 0.0 Hz	24 vs. 28 cells
		-360 pA: LIII vs. LX	>0.9999	(*t*) 0.000 (*df*) 676	0.00 ± 0.0 Hz vs. 0.00 ± 0.0 Hz	24 vs. 28 cells
		-400 pA: LIII vs. LX	>0.9999	(*t*) 0.000 (*df*) 676	0.00 ± 0.0 Hz vs. 0.00 ± 0.0 Hz	24 vs. 28 cells
		-440 pA: LIII vs. LX	0.7388	(*t*) 0.334 (*df*) 676	0.00 ± 0.0 Hz vs. 0.00 ± 0.0 Hz	7 vs. 9 cells
		-480 pA: LIII vs. LX	0.7388	(*t*) 0.334 (*df*) 676	0.00 ± 0.0 Hz vs. 0.00 ± 0.0 Hz	7 vs. 9 cells
		-520 pA: LIII vs. LX	0.7388	(*t*) 0.334 (*df*) 676	0.00 ± 0.0 Hz vs. 0.00 ± 0.0 Hz	7 vs. 9 cells
		-560 pA: LIII vs. LX	0.7388	(*t*) 0.334 (*df*) 676	0.00 ± 0.0 Hz vs. 0.00 ± 0.0 Hz	7 vs. 9 cells
		-600 pA: LIII vs. LX	0.7388	(*t*) 0.334 (*df*) 676	0.00 ± 0.0 Hz vs. 0.00 ± 0.0 Hz	7 vs. 9 cells
		-640 pA: LIII vs. LX	0.7388	(*t*) 0.334 (*df*) 676	0.00 ± 0.0 Hz vs. 0.00 ± 0.0 Hz	7 vs. 9 cells
		-680 pA: LIII vs. LX	0.7388	(*t*) 0.334 (*df*) 676	0.00 ± 0.0 Hz vs. 0.00 ± 0.0 Hz	7 vs. 9 cells
		-720 pA: LIII vs. LX	0.7388	(*t*) 0.334 (*df*) 676	0.00 ± 0.0 Hz vs. 0.00 ± 0.0 Hz	7 vs. 9 cells
		-760 pA: LIII vs. LX	0.7388	(*t*) 0.334 (*df*) 676	0.00 ± 0.0 Hz vs. 0.00 ± 0.0 Hz	7 vs. 9 cells
Figure 2B3	Mixed-effects analysis	-0 pA: LIII vs. LX	**>0.9999**	(*t*) 0.000 (*df*) 676	100 ± 0 % vs. 100 ± 0 %	24 vs. 28 cells
		-40 pA: LIII vs. LX	0.0015	(*t*) 3.189 (*df*) 676	45 ± 12 % vs. 28 ± 6 %	24 vs. 28 cells
		-80 pA: LIII vs. LX	0.0242	(*t*) 2.259 (*df*) 676	17 ± 14 % vs. 6 ± 3 %	24 vs. 28 cells
		-120 pA: LIII vs. LX	**0.4035**	(*t*) 0.000 (*df*) 676	4 ± 3 % vs. 0 ± 0 %	24 vs. 28 cells
		-160 pA: LIII vs. LX	**>0.9999**	(*t*) 0.000 (*df*) 676	0 ± 0 % vs. 0 ± 0 %	24 vs. 28 cells
		-200 pA: LIII vs. LX	**>0.9999**	(*t*) 0.000 (*df*) 676	0 ± 0 % vs. 0 ± 0 %	24 vs. 28 cells
		-240 pA: LIII vs. LX	**>0.9999**	(*t*) 0.000 (*df*) 676	0 ± 0 % vs. 0 ± 0 %	24 vs. 28 cells
		-280 pA: LIII vs. LX	**>0.9999**	(*t*) 0.000 (*df*) 676	0 ± 0 % vs. 0 ± 0 %	24 vs. 28 cells
		-320 pA: LIII vs. LX	**>0.9999**	(*t*) 0.000 (*df*) 676	0 ± 0 % vs. 0 ± 0 %	24 vs. 28 cells
		-360 pA: LIII vs. LX	**>0.9999**	(*t*) 0.000 (*df*) 676	0 ± 0 % vs. 0 ± 0 %	24 vs. 28 cells
		-400 pA: LIII vs. LX	**>0.9999**	(*t*) 0.190 (*df*) 676	0 ± 0 % vs. 0 ± 0 %	24 vs. 28 cells
		-440 pA: LIII vs. LX	**0.8498**	(*t*) 0.190 (*df*) 676	0 ± 0 % vs. 0 ± 0 %	7 vs. 9 cells
		-480 pA: LIII vs. LX	**0.8498**	(*t*) 0.190 (*df*) 676	0 ± 0 % vs. 0 ± 0 %	7 vs. 9 cells
		-520 pA: LIII vs. LX	**0.8498**	(*t*) 0.190 (*df*) 676	0 ± 0 % vs. 0 ± 0 %	7 vs. 9 cells
		-560 pA: LIII vs. LX	**0.8498**	(*t*) 0.190 (*df*) 676	0 ± 0 % vs. 0 ± 0 %	7 vs. 9 cells
		-600 pA: LIII vs. LX	**0.8498**	(*t*) 0.190 (*df*) 676	0 ± 0 % vs. 0 ± 0 %	7 vs. 9 cells
		-640 pA: LIII vs. LX	**0.8498**	(*t*) 0.190 (*df*) 676	0 ± 0 % vs. 0 ± 0 %	7 vs. 9 cells
		-680 pA: LIII vs. LX	**0.8498**	(*t*) 0.190 (*df*) 676	0 ± 0 % vs. 0 ± 0 %	7 vs. 9 cells

		-720 pA: LIII vs. LX	**0.8498**	(*t*) 0.190 (*df*) 676	0 ± 0 % vs. 0 ± 0 %	7 vs. 9 cells
		-760 pA: LIII vs. LX	**0.8498**	(*t*) 0.190 (*df*) 676	0 ± 0 % vs. 0 ± 0 %	7 vs. 9 cells
		0 pA: LIII vs. LX			0.00 ± 0.0 mV	24 vs. 28 cells
		40 pA: LIII vs. LX	0.1283	(*t*) 1.522 (*df*) 1020	2.49 ± 0.1 mV vs. 4.25 ± 0.5 mV	24 vs. 28 cells
		80 pA: LIII vs. LX	0.1128	(*t*) 1.587 (*df*) 1020	8.22 ± 0.9 mV vs. 10.05 ± 0.7 mV	24 vs. 28 cells
		120 pA: LIII vs. LX	0.0658	(*t*) 1.842 (*df*) 1020	11.02 ± 0.8 mV vs. 13.16 ± 0.6 mV	24 vs. 28 cells
		160 pA: LIII vs. LX	0.2259	(*t*) 1.212 (*df*) 1020	13.24 ± 0.7 mV vs. 14.64 ± 0.5 mV	24 vs. 28 cells
		200 pA: LIII vs. LX	0.4621	(*t*) 0.736 (*df*) 1020	14.31 ± 0.5 mV vs. 15.16 ± 0.6 mV	24 vs. 28 cells
		240 pA: LIII vs. LX	0.6287	(*t*) 0.484 (*df*) 1020	14.94 ± 0.5 mV vs. 15.50 ± 0.5 mV	24 vs. 28 cells
		280 pA: LIII vs. LX	0.4827	(*t*) 0.702 (*df*) 1020	15.60 ± 0.5 mV vs. 14.79 ± 0.7 mV	24 vs. 28 cells
		320 pA: LIII vs. LX	0.4296	(*t*) 0.790 (*df*) 1020	15.61 ± 0.6 mV vs. 14.70 ± 0.7 mV	24 vs. 28 cells
Figure	Two-way	360 pA: LIII vs. LX	0.2566	(*t*) 1.135 (*df*) 1020	15.77 ± 0.6 mV vs. 14.45 ± 0.7 mV	24 vs. 28 cells
2C1	ANOVA	400 pA: LIII vs. LX	0.0875	(*t*) 1.711 (*df*) 1020	15.98 ± 0.7 mV vs. 14.00 ± 0.9 mV	24 vs. 28 cells
		440 pA: LIII vs. LX	0.2934	(*t*) 1.051 (*df*) 1020	15.92 ± 0.8 mV vs. 14.70 ± 0.9 mV	7 vs. 9 cells
		480 pA: LIII vs. LX	0.3516	(*t*) 0.932 (*df*) 1020	16.37 ± 1.0 mV vs. 15.29 ± 0.9 mV	7 vs. 9 cells
		520 pA: LIII vs. LX	0.1777	(*t*) 1.349 (*df*) 1020	16.71 ± 1.0 mV vs. 15.15 ± 0.8 mV	7 vs. 9 cells
		560 pA: LIII vs. LX	0.0537	(*t*) 1.931 (*df*) 1020	17.51 ± 1.1 mV vs. 15.28 ± 0.8 mV	7 vs. 9 cells
		600 pA: LIII vs. LX	0.1716	(*t*) 1.368 (*df*) 1020	17.76 ± 1.3 mV vs. 16.18 ± 0.8 mV	7 vs. 9 cells
		640 pA: LIII vs. LX	**0.0299**	(*t*) 2.174 (*df*) 1020	19.33 ± 1.1 mV vs. 16.81 ± 0.9 mV	7 vs. 9 cells
		680 pA: LIII vs. LX	**0.0418**	(*t*) 2.038 (*df*) 1020	20.26 ± 1.2 mV vs. 17.90 ± 0.9 mV	7 vs. 9 cells
		720 pA: LIII vs. LX	**0.0405**	(*t*) 2.051 (*df*) 1020	20.99 ± 1.2 mV vs. 18.61 ± 0.9 mV	7 vs. 9 cells
		760 pA: LIII vs. LX	**0.0380**	(*t*) 2.078 (*df*) 1020	22.01 ± 1.3 mV vs. 19.60 ± 0.9 mV	7 vs. 9 cells
		0 pA: LIII vs. LX	>0.9999	(*t*) 0.000 (*df*) 1020	0.00 ± 0.0 Hz vs. 0.00 ± 0.0 Hz	26 vs. 27 cells
		40 pA: LIII vs. LX	0.8196	(*t*) 0.228 (*df*) 1020	0.00 ± 0.0 Hz vs. 2.37 ± 1.5 Hz	26 vs. 27 cells
		80 pA: LIII vs. LX	0.9230	(*t*) 0.097 (*df*) 1020	16.89 ± 5.1 Hz vs. 17.89 ± 3.6 Hz	26 vs. 27 cells
		120 pA: LIII vs. LX	0.7867	(*t*) 0.271 (*df*) 1020	30.07 ± 6.6 Hz vs. 32.89 ± 4.2 Hz	26 vs. 27 cells
		160 pA: LIII vs. LX	0.8170	(*t*) 0.232 (*df*) 1020	43.92 ± 7.1 Hz vs. 41.52 ± 4.4 Hz	26 vs. 27 cells
		200 pA: LIII vs. LX	0.5404	(*t*) 0.612 (*df*) 1020	55.77 ± 7.7 Hz vs. 49.41 ± 4.5 Hz	26 vs. 27 cells
		240 pA: LIII vs. LX	0.2165	(*t*) 1.237 (*df*) 1020	68.85 ± 7.9 Hz vs. 56.00 ± 4.6 Hz	26 vs. 27 cells
		280 pA: LIII vs. LX	0.1009	(*t*) 1.642 (*df*) 1020	79.62 ± 8.3 Hz vs. 62.56 ± 4.9 Hz	26 vs. 27 cells
		320 pA: LIII vs. LX	**0.0312**	(*t*) 2.157 (*df*) 1020	91.12 ± 8.3 Hz vs. 68.70 ± 5.2 Hz	26 vs. 27 cells
Figure	Two-way	360 pA: LIII vs. LX	**0.0072**	(*t*) 2.694 (*df*) 1020	102.58 ± 8.6 Hz vs. 74.59 ± 5.3 Hz	26 vs. 27 cells
2C2	ANOVA	400 pA: LIII vs. LX	**0.0017**	(*t*) 3.145 (*df*) 1020	113.04 ± 9.0 Hz vs. 80.37 ± 5.5 Hz	26 vs. 27 cells
		440 pA: LIII vs. LX	**0.0003**	(*t*) 3.589 (*df*) 1020	123.77 ± 9.4 Hz vs. 86.48 ± 5.8 Hz	26 vs. 27 cells
		480 pA: LIII vs. LX	**<0.0001**	(*t*) 4.174 (*df*) 1020	134.89 ± 9.8 Hz vs. 91.52 ± 6.0 Hz	26 vs. 27 cells
		520 pA: LIII vs. LX	**<0.0001**	(*t*) 4.602 (*df*) 1020	144.96 ± 10.3 Hz vs. 97.15 ± 6.2 Hz	26 vs. 27 cells
		560 pA: LIII vs. LX	**<0.0001**	(*t*) 5.039 (*df*) 1020	154.54 ± 10.8 Hz vs. 102.19 ± 6.5 Hz	26 vs. 27 cells
		600 pA: LIII vs. LX	**<0.0001**	(*t*) 5.504 (*df*) 1020	163.77 ± 11.2 Hz vs. 106.59 ± 6.6 Hz	26 vs. 27 cells
		640 pA: LIII vs. LX	**<0.0001**	(*t*) 5.976 (*df*) 1020	173.19 ± 11.4 Hz vs. 111.11 ± 6.8 Hz	26 vs. 27 cells
		680 pA: LIII vs. LX	**<0.0001**	(*t*) 6.390 (*df*) 1020	182.27 ± 11.4 Hz vs. 115.89 ± 7.1 Hz	26 vs. 27 cells
		720 pA: LIII vs. LX	**<0.0001**	(*t*) 6.838 (*df*) 1020	191.04 ± 11.4 Hz vs. 120.00 ± 7.2 Hz	26 vs. 27 cells
		760 pA: LIII vs. LX	**<0.0001**	(*t*) 7.335 (*df*) 1020	200.42 ± 11.7 Hz vs. 124.22 ± 7.4 Hz	26 vs. 27 cells
		0 pA: LIII vs. LX	>0.9999	(*t*) 0.000 (*df*) 1020	0 ± 0 % vs. 0 ± 0 %	26 vs. 27 cells
		40 pA: LIII vs. LX	0.4887	(*t*) 0.693 (*df*) 1020	0 ± 0 % vs. 2 ± 1 %	26 vs. 27 cells
		80 pA: LIII vs. LX	**0.0114**	(*t*) 2.535 (*df*) 1020	7 ± 2 % vs. 13 ± 3 %	26 vs. 27 cells
		120 pA: LIII vs. LX	**<0.0001**	(*t*) 4.576 (*df*) 1020	14 ± 2 % vs. 24 ± 2 %	26 vs. 27 cells
		160 pA: LIII vs. LX	**<0.0001**	(*t*) 4.830 (*df*) 1020	20 ± 3 % vs. 31 ± 2 %	26 vs. 27 cells
		200 pA: LIII vs. LX	**<0.0001**	(*t*) 5.250 (*df*) 1020	26 ± 3 % vs. 38 ± 2 %	26 vs. 27 cells
		240 pA: LIII vs. LX	**<0.0001**	(*t*) 4.893 (*df*) 1020	33 ± 2 % vs. 44 ± 2 %	26 vs. 27 cells
		280 pA: LIII vs. LX	**<0.0001**	(*t*) 4.902 (*df*) 1020	38 ± 2 % vs. 49 ± 2 %	26 vs. 27 cells
		320 pA: LIII vs. LX	**<0.0001**	(*t*) 4.533 (*df*) 1020	44 ± 2 % vs. 54 ± 2 %	26 vs. 27 cells
Figure	Two-way	360 pA: LIII vs. LX	**<0.0001**	(*t*) 4.160 (*df*) 1020	50 ± 2 % vs. 59 ± 2 %	26 vs. 27 cells
2C3	ANOVA	400 pA: LIII vs. LX	**<0.0001**	(*t*) 3.957 (*df*) 1020	95 ± 2 % vs. 64 ± 2 %	26 vs. 27 cells
		440 pA: LIII vs. LX	**0.0002**	(*t*) 3.705 (*df*) 1020	61 ± 1 % vs. 69 ± 1 %	26 vs. 27 cells
		480 pA: LIII vs. LX	**0.0025**	(*t*) 3.036 (*df*) 1020	66 ± 1 % vs. 73 ± 1 %	26 vs. 27 cells
		520 pA: LIII vs. LX	**0.0046**	(*t*) 2.842 (*df*) 1020	71 ± 1 % vs. 78 ± 1 %	26 vs. 27 cells
		560 pA: LIII vs. LX	**0.0109**	(*t*) 2.551 (*df*) 1020	76 ± 1 % vs. 82 ± 1 %	26 vs. 27 cells
		600 pA: LIII vs. LX	**0.0398**	(*t*) 2.058 (*df*) 1020	80 ± 1 % vs. 85 ± 1 %	26 vs. 27 cells
		640 pA: LIII vs. LX	0.1273	(*t*) 1.526 (*df*) 1020	86 ± 1 % vs. 89 ± 1 %	26 vs. 27 cells
		680 pA: LIII vs. LX	0.2524	(*t*) 1.145 (*df*) 1020	91 ± 1 % vs. 93 ± 0 %	26 vs. 27 cells
		720 pA: LIII vs. LX	0.5578	(*t*) 0.586 (*df*) 1020	95 ± 0 % vs. 96 ± 0 %	26 vs. 27 cells
		760 pA: LIII vs. LX	>0.9999	(*t*) 0.000 (*df*) 1020	100 ± 0 % vs. 100 ± 0 %	26 vs. 27 cells
Figure 2	Mixed-	-0 pA: LIII vs. LX			0.00 ± 0.0 mV vs. 0.00 ± 0.0 mV	24 vs. 28 cells
S1B	effects	-40 pA: LIII vs. LX	0.6272	(*t*) 0.486 (*df*) 676	0.30 ± 0.1 mV vs. 0.60 ± 0.2 mV	24 vs. 28 cells

	analysis	-80 pA: LIII vs. LX	0.9784	(*t*) 0.027 (*df*) 676	1.33 ± 0.3 mV vs. 1.35 ± 0.2 mV	24 vs. 28 cells
		-120 pA: LIII vs. LX	0.4429	(*t*) 0.768 (*df*) 676	1.85 ± 0.3 mV vs. 2.32 ± 0.3 mV	24 vs. 28 cells
		-160 pA: LIII vs. LX	0.4927	(*t*) 0.686 (*df*) 676	2.70 ± 0.4 mV vs. 3.12 ± 0.4 mV	24 vs. 28 cells
		-200 pA: LIII vs. LX	0.4668	(*t*) 0.728 (*df*) 676	3.54 ± 0.4 mV vs. 3.98 ± 0.4 mV	24 vs. 28 cells
		-240 pA: LIII vs. LX	0.1982	(*t*) 1.288 (*df*) 676	4.22 ± 0.4 mV vs. 4.99 ± 0.4 mV	24 vs. 28 cells
		-280 pA: LIII vs. LX	0.1248	(*t*) 1.537 (*df*) 676	4.94 ± 0.4 mV vs. 5.87 ± 0.4 mV	24 vs. 28 cells
		-320 pA: LIII vs. LX	**0.0381**	(*t*) 2.078 (*df*) 676	5.44 ± 0.5 mV vs. 6.70 ± 0.5 mV	24 vs. 28 cells
		-360 pA: LIII vs. LX	**0.0236**	(*t*) 2.269 (*df*) 676	6.08 ± 0.5 mV vs. 7.45 ± 0.5 mV	24 vs. 28 cells
		-400 pA: LIII vs. LX	**0.0095**	(*t*) 2.600 (*df*) 676	6.69 ± 0.5 mV vs. 8.26 ± 0.6 mV	24 vs. 28 cells
		-440 pA: LIII vs. LX	**0.0155**	(*t*) 2.426 (*df*) 676	6.58 ± 1.0 mV vs. 9.00 ± 1.1 mV	7 vs. 9 cells
		-480 pA: LIII vs. LX	**0.0116**	(*t*) 2.532 (*df*) 676	7.26 ± 0.9 mV vs. 9.76 ± 0.9 mV	7 vs. 9 cells
		-520 pA: LIII vs. LX	**0.0190**	(*t*) 2.350 (*df*) 676	8.18 ± 0.7 mV vs. 10.54 ± 0.8 mV	7 vs. 9 cells
		-560 pA: LIII vs. LX	**0.0494**	(*t*) 1.969 (*df*) 676	8.77 ± 0.7 mV vs. 10.83 ± 1.0 mV	7 vs. 9 cells
		-600 pA: LIII vs. LX	**0.0349**	(*t*) 2.113 (*df*) 676	9.07 ± 0.9 mV vs. 11.24 ± 1.0 mV	7 vs. 9 cells
		-640 pA: LIII vs. LX	0.0576	(*t*) 1.902 (*df*) 676	9.57 ± 1.0 mV vs. 11.58 ± 1.1 mV	7 vs. 9 cells
		-680 pA: LIII vs. LX	0.1962	(*t*) 1.294 (*df*) 676	10.40 ± 1.0 mV vs. 11.93 ± 0.9 mV	7 vs. 9 cells
		-720 pA: LIII vs. LX	0.2001	(*t*) 1.283 (*df*) 676	10.70 ± 1.0 mV vs. 12.22 ± 1.0 mV	7 vs. 9 cells
		-760 pA: LIII vs. LX	0.5226	(*t*) 0.640 (*df*) 676	12.41 ± 1.6 mV vs. 12.42 ± 1.0 mV	7 vs. 9 cells
Figure 2 S1C	Two-way ANOVA	0 pA: LIII vs. LX	>0.9999	(*t*) 0.000 (*df*) 1020	0.00 ± 0.0 mV vs. 0.00 ± 0.0 mV	26 vs. 27 cells
		40 pA: LIII vs. LX	0.7561	(*t*) 0.311 (*df*) 1020	0.07 ± 0.1 mV vs. 0.19 ± 0.1 mV	26 vs. 27 cells
		80 pA: LIII vs. LX	0.6293	(*t*) 0.483 (*df*) 1020	0.91 ± 0.3 mV vs. 1.10 ± 0.3 mV	26 vs. 27 cells
		120 pA: LIII vs. LX	0.5389	(*t*) 0.615 (*df*) 1020	1.47 ± 0.3 mV vs. 1.71 ± 0.2 mV	26 vs. 27 cells
		160 pA: LIII vs. LX	0.7305	(*t*) 0.345 (*df*) 1020	1.97 ± 0.3 mV vs. 2.11 ± 0.2 mV	26 vs. 27 cells
		200 pA: LIII vs. LX	0.5314	(*t*) 0.626 (*df*) 1020	2.37 ± 0.3 mV vs. 2.13 ± 0.2 mV	26 vs. 27 cells
		240 pA: LIII vs. LX	0.5345	(*t*) 0.621 (*df*) 1020	2.61 ± 0.3 mV vs. 2.37 ± 0.3 mV	26 vs. 27 cells
		280 pA: LIII vs. LX	0.5728	(*t*) 0.564 (*df*) 1020	2.72 ± 0.3 mV vs. 2.49 ± 0.2 mV	26 vs. 27 cells
		320 pA: LIII vs. LX	0.2359	(*t*) 1.186 (*df*) 1020	2.95 ± 0.3 mV vs. 2.49 ± 0.2 mV	26 vs. 27 cells
		360 pA: LIII vs. LX	0.6282	(*t*) 0.485 (*df*) 1020	2.98 ± 0.3 mV vs. 2.80 ± 0.3 mV	26 vs. 27 cells
		400 pA: LIII vs. LX	0.3656	(*t*) 0.905 (*df*) 1020	3.13 ± 0.3 mV vs. 2.77 ± 0.3 mV	26 vs. 27 cells
		440 pA: LIII vs. LX	0.4909	(*t*) 0.689 (*df*) 1020	3.12 ± 0.3 mV vs. 2.85 ± 0.3 mV	26 vs. 27 cells
		480 pA: LIII vs. LX	0.6834	(*t*) 0.408 (*df*) 1020	3.09 ± 0.3 mV vs. 2.93 ± 0.3 mV	26 vs. 27 cells
		520 pA: LIII vs. LX	0.8736	(*t*) 0.159 (*df*) 1020	3.15 ± 0.3 mV vs. 3.08 ± 0.3 mV	26 vs. 27 cells
		560 pA: LIII vs. LX	0.9643	(*t*) 0.045 (*df*) 1020	3.20 ± 0.3 mV vs. 3.21 ± 0.4 mV	26 vs. 27 cells
		600 pA: LIII vs. LX	0.8260	(*t*) 0.220 (*df*) 1020	3.26 ± 0.3 mV vs. 3.35 ± 0.4 mV	26 vs. 27 cells
		640 pA: LIII vs. LX	0.7859	(*t*) 0.272 (*df*) 1020	3.35 ± 0.3 mV vs. 3.46 ± 0.4 mV	26 vs. 27 cells
		680 pA: LIII vs. LX	0.9943	(*t*) 0.007 (*df*) 1020	3.43 ± 0.3 mV vs. 3.44 ± 0.4 mV	26 vs. 27 cells
		720 pA: LIII vs. LX	0.7041	(*t*) 0.380 (*df*) 1020	3.47 ± 0.3 mV vs. 3.62 ± 0.4 mV	26 vs. 27 cells
		760 pA: LIII vs. LX	0.8276	(*t*) 0.218 (*df*) 1020	3.52 ± 0.3 mV vs. 3.61 ± 0.4 mV	26 vs. 27 cells

Table S3. Statistical data for in vitro whole cell patch electrophysiology in the adult mouse (P>30), for negative and positive current ramp analysis. Descriptive values are represented as ± SEM. Abbreviations: LIII = Lobule III, LIII↓ = Lobule III downphase at the current ramp, LIII↑ = Lobule III upphase at the current ramp, LX = Lobule X, LX↓ = Lobule X downphase at the current ramp, LX↑ = Lobule X upphase at the current ramp, *df* = degrees of freedom, Hz = hertz, mV = millivolt.

Related Figure	Test Applied	Group	*p*-Value	Population-Value	Descriptive Values	Population Size
Figure 3B1	Two-way ANOVA	-0 pA: LIII vs. LX			0.00 ± 0.0 mV vs. 0.00 ± 0.0 mV	23 vs. 28 cells
		-0 pA: LIII vs. LX	0.9981	(*t*) 0.002 (*df*) 1666	0.06 ± 0.0 mV vs. 0.06 ± 0.07 mV	23 vs. 28 cells
		-0 pA: LIII vs. LX	0.9658	(*t*) 0.043 (*df*) 1666	0.02 ± 0.0 mV vs. -0.02 ± 0.1 mV	23 vs. 28 cells
		-0 pA: LIII vs. LX	0.9491	(*t*) 0.064 (*df*) 1666	-0.04 ± 0.0 mV vs. 0.02 ± 0.1 mV	23 vs. 28 cells
		-0 pA: LIII vs. LX	0.9609	(*t*) 0.049 (*df*) 1666	0.00 ± 0.1 mV vs. 0.04 ± 0.1 mV	23 vs. 28 cells
		-0 pA: LIII vs. LX	0.9969	(*t*) 0.004 (*df*) 1666	-0.02 ± 0.1 mV vs. -0.02 ± 0.1 mV	23 vs. 28 cells
		-0 pA: LIII vs. LX	0.9518	(*t*) 0.060 (*df*) 1666	-0.09 ± 0.1 mV vs. -0.04 ± 0.1 mV	23 vs. 28 cells
		-0 pA: LIII vs. LX	0.8012	(*t*) 0.252 (*df*) 1666	-0.10 ± 0.1 mV vs. 0.10 ± 0.1 mV	23 vs. 28 cells
		-0 pA: LIII vs. LX	0.9786	(*t*) 0.027 (*df*) 1666	-0.11 ± 0.1 mV vs. -0.13 ± 0.1 mV	23 vs. 28 cells
		-0 pA: LIII vs. LX	0.9809	(*t*) 0.024 (*df*) 1666	-0.16 ± 0.1 mV vs.-0.14 ± 0.1 mV	23 vs. 28 cells
		-40 pA: LIII vs. LX	0.7749	(*t*) 0.286 (*df*) 1666	-0.56 ± 0.1 mV vs.-0.79 ± 0.1 mV	23 vs. 28 cells
		-80 pA: LIII vs. LX	0.4059	(*t*) 0.831 (*df*) 1666	-3.53 ± 0.8 mV vs. -2.86 ± 0.5 mV	23 vs. 28 cells
		-120 pA: LIII vs. LX	0.1539	(*t*) 1.426 (*df*) 1666	-11.03 ± 1.2 mV vs. -9.89 ± 1.2 mV	23 vs. 28 cells
		-160 pA: LIII vs. LX	0.3807	(*t*) 0.877 (*df*) 1666	-15.77 ± 0.8 mV vs. -16.47 ± 1.1 mV	23 vs. 28 cells
		-200 pA: LIII vs. LX	**0.0019**	(*t*) 3.113 (*df*) 1666	-17.77 ± 0.8 mV vs. -20.26 ± 0.7mV	23 vs. 28 cells
		-240 pA: LIII vs. LX	**0.0005**	(*t*) 3.481 (*df*) 1666	-19.94 ± 0.7 mV vs. -22.73 ± 0.8 mV	23 vs. 28 cells
		-280 pA: LIII vs. LX	**0.0002**	(*t*) 3.781 (*df*) 1666	-21.87 ± 0.8 mV vs. -24.90 ± 0.9 mV	23 vs. 28 cells
		-320 pA: LIII vs. LX	**<0.0001**	(*t*) 4.025 (*df*) 1666	-23.35 ± 0.8 mV vs. -26.57 ± 0.9 mV	23 vs. 28 cells
		-280 pA: LIII vs. LX	**0.0023**	(*t*) 3.059 (*df*) 1666	-20.95 ± 0.7 mV vs. -23.41 ± 0.9 mV	23 vs. 28 cells
		-240 pA: LIII vs. LX	**0.0146**	(*t*) 2.444 (*df*) 1666	-19.19 ± 0.7 mV vs. -21.15 ± 0.8 mV	23 vs. 28 cells
		-200 pA: LIII vs. LX	0.1012	(*t*) 1.640 (*df*) 1666	-17.31 ± 0.7 mV vs. -18.63 ± 0.8 mV	23 vs. 28 cells
		-160 pA: LIII vs. LX	0.2155	(*t*) 1.239 (*df*) 1666	-15.14 ± 0.7 mV vs. -16.13 ± 0.8 mV	23 vs. 28 cells
		-120 pA: LIII vs. LX	0.4816	(*t*) 0.704 (*df*) 1666	-12.73 ± 0.7 mV vs. -13.30 ± 0.7 mV	23 vs. 28 cells
		-80 pA: LIII vs. LX	0.9268	(*t*) 0.092 (*df*) 1666	-10.24 ± 0.7 mV vs. -10.31 ± 0.7 mV	23 vs. 28 cells
		-40 pA: LIII vs. LX	0.3062	(*t*) 1.024 (*df*) 1666	-7.48 ± 0.7 mV vs. -6.66 ± 0.8 mV	23 vs. 28 cells
		-0 pA: LIII vs. LX	0.8513	(*t*) 0.188 (*df*) 1666	-2.29 ± 0.6 mV vs. -2.44 ± 0.7 mV	23 vs. 28 cells
		-0 pA: LIII vs. LX	0.3672	(*t*) 0.902 (*df*) 1666	-0.81 ± 0.4 mV vs. -1.53 ± 0.6 mV	23 vs. 28 cells
		-0 pA: LIII vs. LX	0.5021	(*t*) 0.671 (*df*) 1666	-0.48 ± 0.4 mV vs.-1.02 ± 0.5 mV	23 vs. 28 cells
		-0 pA: LIII vs. LX	0.5332	(*t*) 0.623 (*df*) 1666	-0.19 ± 0.2 mV vs. -0.69 ± 0.5 mV	23 vs. 28 cells
		-0 pA: LIII vs. LX	0.5857	(*t*) 0.545 (*df*) 1666	-0.09 ± 0.2 mV vs. -0.53 ± 0.4 mV	23 vs. 28 cells
		-0 pA: LIII vs. LX	0.5030	(*t*) 0.670 (*df*) 1666	0.12 ± 0.2 mV vs. -0.41 ± 0.4 mV	23 vs. 28 cells
		-0 pA: LIII vs. LX	0.6352	(*t*) 0.475 (*df*) 1666	0.12 ± 0.1 mV vs.-0.26 ± 0.3 mV	23 vs. 28 cells
		-0 pA: LIII vs. LX	0.7281	(*t*) 0.348 (*df*) 1666	0.11 ± 0.1 mV vs. -0.17 ± 0.2 mV	23 vs. 28 cells
		-0 pA: LIII vs. LX	0.9379	(*t*) 0.078 (*df*) 1666	0.19 ± 0.1 mV vs. 0.13 ± 0.2 mV	23 vs. 28 cells
Figure 3B2	Two-way ANOVA	-0 pA: LIII↓ vs. LIII↑	0.7678	(*t*) 0.276 (*df*) 347	0.06 ± 0.0 mV vs. 0.19 ± 0.1 mV	23 cells
		-0 pA: LIII↓ vs. LIII↑	0.8409	(*t*) 0.188 (*df*) 347	0.02 ± 0.0 mV vs. 0.11 ± 0.1 mV	
		-0 pA: LIII↓ vs. LIII↑	0.7312	(*t*) 0.321 (*df*) 347	-0.04 ± 0.0 mV vs. 0.12 ± 0.1 mV	
		-0 pA: LIII↓ vs. LIII↑	0.7915	(*t*) 0.247 (*df*) 347	0.00 ± 0.1 mV vs. 0.12 ± 0.2 mV	
		-0 pA: LIII↓ vs. LIII↑	0.8788	(*t*) 0.143 (*df*) 347	-0.02 ± 0.1 mV vs. -0.09 ± 0.2 mV	
		-0 pA: LIII↓ vs. LIII↑	0.8116	(*t*) 0.223 (*df*) 347	-0.09 ± 0.1 mV vs. -0.19 ± 0.2 mV	
		-0 pA: LIII↓ vs. LIII↑	0.3987	(*t*) 0.789 (*df*) 347	-0.10 ± 0.1 mV vs. -0.49 ± 0.4 mV	
		-0 pA: LIII↓ vs. LIII↑	0.1200	(*t*) 1.455 (*df*) 347	-0.11 ± 0.1 mV vs. -0.81 ± 0.4 mV	
		-0 pA: LIII↓ vs. LIII↑	**<0.0001**	(*t*) 4.433 (*df*) 347	-0.16 ± 0.1 mV vs. -2.29 ± 0.5 mV	
		-40 pA: LIII↓ vs. LIII↑	**<0.0001**	(*t*) 14.36 (*df*) 347	-0.56 ± 0.1 mV vs. -7.48 ± 0.6 mV	
		-80 pA: LIII↓ vs. LIII↑	**<0.0001**	(*t*) 13.92 (*df*) 347	-3.53 ± 0.1 mV vs. -10.24 ± 0.7 mV	
		-120 pA: LIII↓ vs. LIII↑	**0.0002**	(*t*) 3.521 (*df*) 347	-11.03 ± 1.2 mV vs. -12.73 ± 0.7 mV	
		-160 pA: LIII↓ vs. LIII↑	0.1629	(*t*) 1.306 (*df*) 347	-15.77 ± 0.8 mV vs. -15.14 ± 0.7 mV	
		-200 pA: LIII↓ vs. LIII↑	0.3150	(*t*) 0.937 (*df*) 347	-17.77 ± 0.8 mV vs. -17.31 ± 0.7 mV	
		-240 pA: LIII↓ vs. LIII↑	0.0964	(*t*) 1.556 (*df*) 347	-19.94 ± 0.7 mV vs. -19.19 ± 0.7 mV	
		-280 pA: LIII↓ vs. LIII↑	**0.0431**	(*t*) 1.895 (*df*) 347	-21.87 ± 0.8 mV vs. -20.95 ± 0.7 mV	
		-320 pA			-23.35 ± 0.8 mV	
Figure 3B3	Two-way ANOVA	-0 pA: LX↓ vs. LX↑	0.8868	(*t*) 0.142 (*df*) 432	0.06 ± 0.1 mV vs. 0.13 ± 0.2 mV	28 cells
		-0 pA: LX↓ vs. LX↑	0.7625	(*t*) 0.302 (*df*) 432	-0.02 ± 0.1 mV vs. -0.17 ± 0.2 mV	
		-0 pA: LX↓ vs. LX↑	0.5870	(*t*) 0.544 (*df*) 432	0.02 ± 0.1 mV vs. -0.26 ± 0.3 mV	
		-0 pA: LX↓ vs. LX↑	0.3697	(*t*) 0.898 (*df*) 432	0.04 ± 0.1 mV vs. -0.41 ± 0.4 mV	
		-0 pA: LX↓ vs. LX↑	0.3242	(*t*) 0.987 (*df*) 432	-0.02 ± 0.1 mV vs. -0.53 ± 0.4 mV	
		-0 pA: LX↓ vs. LX↑	0.1987	(*t*) 1.287 (*df*) 432	-0.04 ± 0.1 mV vs. -0.69 ± 0.5 mV	

		-0 pA: LX↓ vs. LX↑	**0.0283**	(*t*) 2.201 (*df*) 432	0.10 ± 0.1 mV vs. -1.02 ± 0.5 mV	
		-0 pA: LX↓ vs. LX↑	**0.0061**	(*t*) 2.756 (*df*) 432	-0.13 ± 0.1 mV vs. -1.53 ± 0.6 mV	
		-0 pA: LX↓ vs. LX↑	**<0.0001**	(*t*) 4.531 (*df*) 432	-0.14 ± 0.1 mV vs. -2.44 ± 0.7 mV	
		-40 pA: LX↓ vs. LX↑	**<0.0001**	(*t*) 11.540 (*df*) 432	-0.79 ± 0.1 mV vs. -6.66 ± 0.8 mV	
		-80 pA: LX↓ vs. LX↑	**<0.0001**	(*t*) 14.630 (*df*) 432	-2.86 ± 0.6 mV vs. -10.31 ± 0.7 mV	
		-120 pA: LX↓ vs. LX↑	**<0.0001**	(*t*) 6.689 (*df*) 432	-9.89 ± 1.2 mV vs. -13.30 ± 0.7 mV	
		-160 pA: LX↓ vs. LX↑	0.5055	(*t*) 0.666 (*df*) 432	-16.47 ± 1.1 mV vs. -16.13 ± 0.8 mV	
		-200 pA: LX↓ vs. LX↑	**0.0014**	(*t*) 3.208 (*df*) 432	-20.26 ± 0.7 mV vs. -18.83 ± 0.8 mV	
		-240 pA: LX↓ vs. LX↑	**0.0020**	(*t*) 3.106 (*df*) 432	-22.73 ± 0.8 mV vs. -21.15 ± 0.8 mV	
		-280 pA: LX↓ vs. LX↑	**0.0035**	(*t*) 2.933 (*df*) 432	-24.89 ± 0.9 mV vs. -23.41 ± 0.9 mV	
		-320 pA			-26.54 ± 0.9 mV	
Figure 3C1	Two-way ANOVA	-0 pA: LIII vs. LX	**0.0412**	(*t*) 2.043 (*df*) 1666	39.82 ± 6.2 Hz vs. 29.31 ± 3.3 Hz	23 vs. 28 cells
		-0 pA: LIII vs. LX	**0.0252**	(*t*) 2.240 (*df*) 1666	41.01 ± 6.3 Hz vs. 29.50 ± 3.3 Hz	23 vs. 28 cells
		-0 pA: LIII vs. LX	**0.0287**	(*t*) 2.189 (*df*) 1666	41.12 ± 6.4 Hz vs. 29.87 ± 3.4 Hz	23 vs. 28 cells
		-0 pA: LIII vs. LX	**0.0264**	(*t*) 2.223 (*df*) 1666	41.44 ± 6.4 Hz vs. 30.02 ± 3.4 Hz	23 vs. 28 cells
		-0 pA: LIII vs. LX	**0.0198**	(*t*) 2.33 (*df*) 1666	42.24 ± 6.5 Hz vs. 30.00 ± 3.4 Hz	23 vs. 28 cells
		-0 pA: LIII vs. LX	**0.0163**	(*t*) 2.404 (*df*) 1666	42.62 ± 6.5 Hz vs. 30.24 ± 3.4 Hz	23 vs. 28 cells
		-0 pA: LIII vs. LX	**0.0162**	(*t*) 2.407 (*df*) 1666	42.76 ± 6.5 Hz vs. 30.44 ± 3.5 Hz	23 vs. 28 cells
		-0 pA: LIII vs. LX	**0.0166**	(*t*) 2.397 (*df*) 1666	43.01 ± 6.6 Hz vs. 30.57 ± 3.4 Hz	23 vs. 28 cells
		-0 pA: LIII vs. LX	**0.0156**	(*t*) 2.421 (*df*) 1666	43.05 ± 6.6 Hz vs. 30.30 ± 3.5 Hz	23 vs. 28 cells
		-0 pA: LIII vs. LX	**0.0129**	(*t*) 2.488 (*df*) 1666	32.05 ± 6.1 Hz vs. 23.70 ± 3.3 Hz	23 vs. 28 cells
		-40 pA: LIII vs. LX	0.1045	(*t*) 1.624 (*df*) 1666	14.76 ± 4.7 Hz vs. 11.53 ± 2.9 Hz	23 vs. 28 cells
		-80 pA: LIII vs. LX	0.5305	(*t*) 0.627 (*df*) 1666	4.68 ± 2.18 Hz vs. 4.21 ± 2.0 Hz	23 vs. 28 cells
		-120 pA: LIII vs. LX	0.9267	(*t*) 0.091 (*df*) 1666	0.46 ± 0.38 Hz vs. 1.30 ± 0.9 Hz	23 vs. 28 cells
		-160 pA: LIII vs. LX	0.8696	(*t*) 0.164 (*df*) 1666	0.00 ± 0.0 Hz vs. 0.09 ± 0.1 Hz	23 vs. 28 cells
		-200 pA: LIII vs. LX	0.9861	(*t*) 0.017 (*df*) 1666	0.00 ± 0.0 Hz vs. 0.00 ± 0.0 Hz	23 vs. 28 cells
		-240 pA: LIII vs. LX	>0.9999	(*t*) 0.000 (*df*) 1666	0.00 ± 0.0 Hz vs. 0.00 ± 0.0 Hz	23 vs. 28 cells
		-280 pA: LIII vs. LX	>0.9999	(*t*) 0.000 (*df*) 1666	0.00 ± 0.0 Hz vs. 0.00 ± 0.0 Hz	23 vs. 28 cells
		-320 pA: LIII vs. LX	>0.9999	(*t*) 0.000 (*df*) 1666	0.00 ± 0.0 Hz vs. 0.00 ± 0.0 Hz	23 vs. 28 cells
		-280 pA: LIII vs. LX	>0.9999	(*t*) 0.000 (*df*) 1666	0.00 ± 0.0 Hz vs. 0.00 ± 0.0 Hz	23 vs. 28 cells
		-240 pA: LIII vs. LX	>0.9999	(*t*) 0.000 (*df*) 1666	0.00 ± 0.0 Hz vs. 0.00 ± 0.0 Hz	23 vs. 28 cells
		-200 pA: LIII vs. LX	>0.9999	(*t*) 0.000 (*df*) 1666	0.00 ± 0.0 Hz vs. 0.00 ± 0.0 Hz	23 vs. 28 cells
		-160 pA: LIII vs. LX	>0.9999	(*t*) 0.000 (*df*) 1666	0.00 ± 0.0 Hz vs. 0.00 ± 0.0 Hz	23 vs. 28 cells
		-120 pA: LIII vs. LX	0.8919	(*t*) 0.136 (*df*) 1666	0.70 ± 0.6 Hz vs. 0.00 ± 0.0 Hz	23 vs. 28 cells
		-80 pA: LIII vs. LX	0.7853	(*t*) 0.273 (*df*) 1666	1.47 ± 1.1 Hz vs. 0.07 ± 0.1 Hz	23 vs. 28 cells
		-40 pA: LIII vs. LX	0.8375	(*t*) 0.205 (*df*) 1666	3.93 ± 1.9 Hz vs. 2.88 ± 1.4 Hz	23 vs. 28 cells
		-0 pA: LIII vs. LX	0.1714	(*t*) 1.368 (*df*) 1666	17.89 ± 5.3 Hz vs. 10.86 ± 2.3 Hz	23 vs. 28 cells
		-0 pA: LIII vs. LX	0.1105	(*t*) 1.597 (*df*) 1666	25.49 ± 5.5 Hz vs. 17.28 ± 2.9 Hz	23 vs. 28 cells
		-0 pA: LIII vs. LX	0.1499	(*t*) 1.441 (*df*) 1666	27.46 ± 5.5 Hz vs. 20.06 ± 3.1 Hz	23 vs. 28 cells
		-0 pA: LIII vs. LX	0.1471	(*t*) 1.450 (*df*) 1666	29.65 ± 5.6 Hz vs. 22.19 ± 3.1 Hz	23 vs. 28 cells
		-0 pA: LIII vs. LX	0.1142	(*t*) 1.580 (*df*) 1666	31.50 ± 5.6 Hz vs. 23.38 ± 3.1 Hz	23 vs. 28 cells
		-0 pA: LIII vs. LX	0.1240	(*t*) 1.539 (*df*) 1666	32.22 ± 5.7 Hz vs. 24.31 ± 3.0 Hz	23 vs. 28 cells
		-0 pA: LIII vs. LX	0.0728	(*t*) 1.795 (*df*) 1666	34.99 ± 5.7 Hz vs. 25.77 ± 3.0 Hz	23 vs. 28 cells
		-0 pA: LIII vs. LX	0.0583	(*t*) 1.895 (*df*) 1666	36.00 ± 5.9 Hz vs. 26.26 ± 3.0 Hz	23 vs. 28 cells
		-0 pA: LIII vs. LX	0.0602	(*t*) 1.881 (*df*) 1666	37.28 ± 6.1 Hz vs. 27.61 ± 3.0 Hz	23 vs. 28 cells
Figure 3C2	Mixed-effects analysis	-0 pA: LIII↓ vs. LIII↑	0.0891	(*t*) 1.705 (*df*) 352	41.01 ± 6.3 Hz vs. 37.28 ± 6.1 Hz	23 cells
		-0 pA: LIII↓ vs. LIII↑	**0.0200**	(*t*) 2.337 (*df*) 352	41.12 ± 6.4 Hz vs. 36.00 ± 5.9 Hz	
		-0 pA: LIII↓ vs. LIII↑	**0.0167**	(*t*) 2.943 (*df*) 352	41.44 ± 6.4 Hz vs. 34.99 ± 5.7 Hz	
		-0 pA: LIII↓ vs. LIII↑	**0.0002**	(*t*) 4.568 (*df*) 352	42.24 ± 6.5 Hz vs. 32.22 ± 5.7 Hz	
		-0 pA: LIII↓ vs. LIII↑	**<0.0001**	(*t*) 4.949 (*df*) 352	42.35 ± 6.4 Hz vs. 31.50 ± 5.6 Hz	
		-0 pA: LIII↓ vs. LIII↑	**<0.0001**	(*t*) 5.917 (*df*) 352	42.62 ± 6.5 Hz vs. 29.65 ± 5.6 Hz	
		-0 pA: LIII↓ vs. LIII↑	**<0.0001**	(*t*) 6.979 (*df*) 352	42.76 ± 6.5 Hz vs. 27.46 ± 5.5 Hz	
		-0 pA: LIII↓ vs. LIII↑	**<0.0001**	(*t*) 7.990 (*df*) 352	43.01 ± 6.6 Hz vs. 25.49 ± 5.5 Hz	
		-0 pA: LIII↓ vs. LIII↑	**<0.0001**	(*t*) 11.48 (*df*) 352	43.05 ± 6.6 Hz vs. 17.89 ± 5.3 Hz	
		-40 pA: LIII↓ vs. LIII↑	**<0.0001**	(*t*) 12.83 (*df*) 352	32.05 ± 6.1 Hz vs. 3.93 ± 1.9 Hz	
		-80 pA: LIII↓ vs. LIII↑	**<0.0001**	(*t*) 6.061 (*df*) 352	14.76 ± 4.7 Hz vs. 1.47 ± 1.1 Hz	
		-120 pA: LIII↓ vs. LIII↑	0.0700	(*t*) 1.817 (*df*) 352	4.68 ± 2.2 Hz vs. 0.70 ± 0.6 Hz	
		-160 pA: LIII↓ vs. LIII↑	0.8341	(*t*) 0.210 (*df*) 352	0.46 ± 0.4 Hz vs. 0.00 ± 0.00 Hz	
		-200 pA: LIII↓ vs. LIII↑	>0.9999	(*t*) 0.000 (*df*) 352	0.00 ± 0.0 Hz vs. 0.00 ± 0.00 Hz	
		-240 pA: LIII↓ vs. LIII↑	>0.9999	(*t*) 0.000 (*df*) 352	0.00 ± 0.0 Hz vs. 0.00 ± 0.00 Hz	
		-280 pA: LIII↓ vs. LIII↑	>0.9999	(*t*) 0.000 (*df*) 352	0.00 ± 0.0 Hz vs. 0.00 ± 0.00 Hz	
		-320 pA			0.00 ± 0.0 Hz	
Figure 3C3	Mixed-effects	-0 pA: LX↓ vs. LX↑	0.2304	(*t*) 0.836 (*df*) 459	29.50 ± 3.3 Hz vs. 27.61 ± 3.0 Hz	28 cells
		-0 pA: LX↓ vs. LX↑	**0.0222**	(*t*) 1.597 (*df*) 459	29.87 ± 3.4 Hz vs. 26.26 ± 3.0 Hz	

	analysis	-0 pA: LX↓ vs. LX↑	**0.0071**	(*t*) 1.882 (*df*) 459	30.02 ± 3.4 Hz vs. 25.77 ± 3.0 Hz	
		-0 pA: LX↓ vs. LX↑	**0.0002**	(*t*) 2.628 (*df*) 459	30.25 ± 3.4 Hz vs. 24.31 ± 3.0 Hz	
		-0 pA: LX↓ vs. LX↑	**<0.0001**	(*t*) 2.928 (*df*) 459	30.00 ± 3.4 Hz vs. 23.38 ± 3.1 Hz	
		-0 pA: LX↓ vs. LX↑	**<0.0001**	(*t*) 3.563 (*df*) 459	30.24 ± 3.5 Hz vs. 22.19 ± 3.1 Hz	
		-0 pA: LX↓ vs. LX↑	**<0.0001**	(*t*) 4.594 (*df*) 459	30.43 ± 3.4 Hz vs. 20.06 ± 3.1 Hz	
		-0 pA: LX↓ vs. LX↑	**<0.0001**	(*t*) 5.877 (*df*) 459	30.57 ± 3.5 Hz vs. 17.28 ± 2.9 Hz	
		-0 pA: LX↓ vs. LX↑	**<0.0001**	(*t*) 8.585 (*df*) 459	30.26 ± 3.5 Hz vs. 10.86 ± 2.3 Hz	
		-40 pA: LX↓ vs. LX↑	**<0.0001**	(*t*) 9.216 (*df*) 459	23.70 ± 3.3 Hz vs. 2.88 ± 1.4 Hz	
		-80 pA: LX↓ vs. LX↑	**<0.0001**	(*t*) 5.072 (*df*) 459	11.53 ± 2.9 Hz vs. 0.07 ± 0.1 Hz	
		-120 pA: LX↓ vs. LX↑	**0.0077**	(*t*) 1.863 (*df*) 459	4.21 ± 2.0 Hz vs. 0.00 ± 0.0 Hz	
		-160 pA: LX↓ vs. LX↑	0.4075	(*t*) 0.577 (*df*) 459	1.30 ± 0.9 Hz vs. 0.00 ± 0.0 Hz	
		-200 pA: LX↓ vs. LX↑	0.9547	(*t*) 0.040 (*df*) 459	0.09 ± 0.1 Hz vs. 0.00 ± 0.0 Hz	
		-240 pA: LX↓ vs. LX↑	>0.9999	(*t*) 0.000 (*df*) 459	0.00 ± 0.0 Hz vs. 0.00 ± 0.0 Hz	
		-280 pA: LX↓ vs. LX↑	>0.9999	(*t*) 0.000 (*df*) 459	0.00 ± 0.0 Hz vs. 0.00 ± 0.0 Hz	
		-320 pA			0.00 ± 0.0 Hz	
Figure 3D1	Mixed-effects analysis	0 pA: LIII vs. LX			0.00 ± 0.0 mV vs. 0.00 ± 0.0 mV	26 vs. 27 cells
		0 pA: LIII vs. LX	0.8868	(*t*) 0.142 (*df*) 1694	0.00 ± 0.0 mV vs. 0.02 ± 0.0 mV	26 vs. 27 cells
		0 pA: LIII vs. LX	0.9017	(*t*) 0.124 (*df*) 1694	-0.01 ± 0.0 mV vs. 0.02 ± 0.0 mV	26 vs. 27 cells
		0 pA: LIII vs. LX	0.8848	(*t*) 0.145 (*df*) 1694	0.00 ± 0.0 mV vs. 0.02 ± 0.0 mV	26 vs. 27 cells
		0 pA: LIII vs. LX	0.9079	(*t*) 0.116 (*df*) 1694	0.01 ± 0.0 mV vs. 0.05 ± 0.0 mV	26 vs. 27 cells
		0 pA: LIII vs. LX	0.9282	(*t*) 0.090 (*df*) 1694	0.01 ± 0.0 mV vs. 0.07 ± 0.0 mV	26 vs. 27 cells
		0 pA: LIII vs. LX	0.911	(*t*) 0.117 (*df*) 1694	0.03 ± 0.0 mV vs. 0.10 ± 0.0 mV	26 vs. 27 cells
		0 pA: LIII vs. LX	0.8817	(*t*) 0.149 (*df*) 1694	0.03 ± 0.0 mV vs. 0.13 ± 0.0 mV	26 vs. 27 cells
		0 pA: LIII vs. LX	0.9276	(*t*) 0.091 (*df*) 1694	0.04 ± 0.0 mV vs. 0.10 ± 0.0 mV	26 vs. 27 cells
		0 pA: LIII vs. LX	0.8813	(*t*) 0.149 (*df*) 1694	0.04 ± 0.0 mV vs. 0.14 ± 0.0 mV	26 vs. 27 cells
		40 pA: LIII vs. LX	0.7201	(*t*) 0.359 (*df*) 1694	0.52 ± 0.1 mV vs. 0.76 ± 0.2 mV	26 vs. 27 cells
		80 pA: LIII vs. LX	**0.0412**	(*t*) 2.043 (*df*) 1694	3.83 ± 0.1 mV vs. 5.18 ± 0.3 mV	26 vs. 27 cells
		120 pA: LIII vs. LX	**0.0331**	(*t*) 2.132 (*df*) 1694	7.82 ± 0.6 mV vs. 9.23 ± 0.5 mV	26 vs. 27 cells
		160 pA: LIII vs. LX	**0.0179**	(*t*) 2.371 (*df*) 1694	11.50 ± 0.6 mV vs. 13.06 ± 0.5 mV	26 vs. 27 cells
		200 pA: LIII vs. LX	0.3822	(*t*) 0.874 (*df*) 1694	14.33 ± 0.6 mV vs. 14.91 ± 0.5 mV	26 vs. 27 cells
		240 pA: LIII vs. LX	0.3957	(*t*) 0.850 (*df*) 1694	15.74 ± 0.5 mV vs. 16.30 ± 0.7 mV	26 vs. 27 cells
		280 pA: LIII vs. LX	0.9212	(*t*) 0.099 (*df*) 1694	16.40 ± 0.5 mV vs. 16.46 ± 0.7 mV	26 vs. 27 cells
		320 pA: LIII vs. LX	0.3040	(*t*) 1.028 (*df*) 1694	16.34 ± 0.6 mV vs. 15.66 ± 0.9 mV	26 vs. 27 cells
		280 pA: LIII vs. LX	0.7905	(*t*) 0.266 (*df*) 1694	16.64 ± 0.6 mV vs. 16.46 ± 0.7 mV	26 vs. 27 cells
		240 pA: LIII vs. LX	0.8633	(*t*) 0.172 (*df*) 1694	16.20 ± 0.5 mV vs. 16.09 ± 0.7 mV	26 vs. 27 cells
		200 pA: LIII vs. LX	0.6796	(*t*) 0.413 (*df*) 1694	15.01 ± 0.5 mV vs. 15.28 ± 0.7 mV	26 vs. 27 cells
		160 pA: LIII vs. LX	**0.0445**	(*t*) 2.011 (*df*) 1694	13.24 ± 0.7 mV vs. 14.57 ± 0.7 mV	26 vs. 27 cells
		120 pA: LIII vs. LX	**0.0157**	(*t*) 2.419 (*df*) 1694	11.43 ± 0.9 mV vs. 13.03 ± 0.8 mV	26 vs. 27 cells
		80 pA: LIII vs. LX	**<0.0001**	(*t*) 4.810 (*df*) 1694	7.95 ± 1.0 mV vs. 11.13 ± 1.0 mV	26 vs. 27 cells
		40 pA: LIII vs. LX	**<0.0001**	(*t*) 6.316 (*df*) 1694	3.82 ± 1.1 mV vs. 7.99 ± 1.2 mV	26 vs. 27 cells
		0 pA: LIII vs. LX	**0.0405**	(*t*) 2.050 (*df*) 1694	0.03 ± 0.4 mV vs. 1.39 ± 0.7 mV	26 vs. 27 cells
		0 pA: LIII vs. LX	0.6118	(*t*) 0.508 (*df*) 1694	0.05 ± 0.1 mV vs. -0.29 ± 0.2 mV	26 vs. 27 cells
		0 pA: LIII vs. LX	0.6395	(*t*) 0.469 (*df*) 1694	0.11 ± 0.1 mV vs. -0.20 ± 0.1 mV	26 vs. 27 cells
		0 pA: LIII vs. LX	0.7359	(*t*) 0.337 (*df*) 1694	0.04 ± 0.0 mV vs. -0.18 ± 0.0 mV	26 vs. 27 cells
		0 pA: LIII vs. LX	0.6687	(*t*) 0.428 (*df*) 1694	0.06 ± 0.0 mV vs. -0.11 ± 0.0 mV	26 vs. 27 cells
		0 pA: LIII vs. LX	0.7170	(*t*) 0.363 (*df*) 1694	0.05 ± 0.0 mV vs. -0.07 ± 0.0 mV	26 vs. 27 cells
		0 pA: LIII vs. LX	0.7840	(*t*) 0.247 (*df*) 1694	0.03 ± 0.0 mV vs. -0.03 ± 0.0 mV	26 vs. 27 cells
		0 pA: LIII vs. LX	0.8254	(*t*) 0.221 (*df*) 1694	0.04 ± 0.0 mV vs. 0.01 ± 0.0 mV	26 vs. 27 cells
		0 pA: LIII vs. LX	0.7569	(*t*) 0.310 (*df*) 1694	0.04 ± 0.0 mV vs. -0.05 ± 0.1 mV	26 vs. 27 cells
Figure 3D2	Mixed-effects analysis	0 pA: LIII↓ vs. LIII↑	0.9151	(*t*) 0.107 (*df*) 416	0.00 ± 0.0 mV vs. 0.04 ± 0.0 mV	26 cells
		0 pA: LIII↓ vs. LIII↑	0.8991	(*t*) 0.127 (*df*) 416	-0.10 ± 0.0 mV vs. 0.04 ± 0.0 mV	
		0 pA: LIII↓ vs. LIII↑	0.9263	(*t*) 0.093 (*df*) 416	0.00 ± 0.0 mV vs. 0.03 ± 0.0 mV	
		0 pA: LIII↓ vs. LIII↑	0.9033	(*t*) 0.122 (*df*) 416	0.01 ± 0.0 mV vs. 0.05 ± 0.0 mV	
		0 pA: LIII↓ vs. LIII↑	0.8708	(*t*) 0.163 (*df*) 416	0.01 ± 0.0 mV vs. 0.06 ± 0.0 mV	
		0 pA: LIII↓ vs. LIII↑	0.9752	(*t*) 0.031 (*df*) 416	0.03 ± 0.0 mV vs. 0.04 ± 0.0 mV	
		0 pA: LIII↓ vs. LIII↑	0.8540	(*t*) 0.184 (*df*) 416	0.03 ± 0.0 mV vs. 0.11 ± 0.1 mV	
		0 pA: LIII↓ vs. LIII↑	0.9793	(*t*) 0.026 (*df*) 416	0.04 ± 0.0 mV vs. 0.05 ± 0.1 mV	
		0 pA: LIII↓ vs. LIII↑	0.9917	(*t*) 0.010 (*df*) 416	0.04 ± 0.0 mV vs. 0.03 ± 0.4 mV	
		40 pA: LIII↓ vs. LIII↑	**<0.0001**	(*t*) 7.420 (*df*) 416	0.52 ± 0.1 mV vs. 3.82 ± 1.1 mV	
		80 pA: LIII↓ vs. LIII↑	**<0.0001**	(*t*) 9.265 (*df*) 416	3.83 ± 0.1 mV vs. 7.95 ± 1.0 mV	
		120 pA: LIII↓ vs. LIII↑	**<0.0001**	(*t*) 8.126 (*df*) 416	7.82 ± 0.6 mV vs. 11.43 ± 0.9 mV	
		160 pA: LIII↓ vs. LIII↑	**0.0001**	(*t*) 3.918 (*df*) 416	11.50 ± 0.6 mV vs. 13.24 ± 0.7 mV	
		200 pA: LIII↓ vs. LIII↑	0.1301	(*t*) 1.517 (*df*) 416	14.33 ± 0.6 mV vs. 15.01 ± 0.5 mV	
		240 pA: LIII↓ vs. LIII↑	0.3025	(*t*) 1.032 (*df*) 416	15.74 ± 0.5 mV vs. 16.20 ± 0.5 mV	

		280 pA: LIII↓ vs. LIII↑	0.5926	(*t*) 0.535 (*df*) 416	16.40 ± 0.5 mV vs. 16.64 ± 0.6 mV	
		320 pA			16.34 ± 0.6 mV	
		0 pA: LX↓ vs. LX↑	0.8721	(*t*) 0.161 (*df*) 415	0.02 ± 0.0 mV vs. -0.05 ± 0.1 mV	
		0 pA: LX↓ vs. LX↑	0.9535	(*t*) 0.058 (*df*) 415	0.02 ± 0.0 mV vs. 0.01 ± 0.0 mV	
		0 pA: LX↓ vs. LX↑	0.8999	(*t*) 0.126 (*df*) 415	0.02 ± 0.0 mV vs. -0.03 ± 0.0 mV	
		0 pA: LX↓ vs. LX↑	0.8054	(*t*) 0.247 (*df*) 415	0.05 ± 0.0 mV vs. -0.07 ± 0.0 mV	
		0 pA: LX↓ vs. LX↑	0.6601	(*t*) 0.440 (*df*) 415	0.07 ± 0.0 mV vs. -0.11 ± 0.0 mV	
		0 pA: LX↓ vs. LX↑	0.5917	(*t*) 0.537 (*df*) 415	0.10 ± 0.0 mV vs. -0.18 ± 0.0 mV	
		0 pA: LX↓ vs. LX↑	0.5366	(*t*) 0.619 (*df*) 415	0.13 ± 0.0 mV vs. -0.20 ± 0.1 mV	
		0 pA: LX↓ vs. LX↑	0.4664	(*t*) 0.729 (*df*) 415	0.10 ± 0.0 mV vs. -0.29 ± 0.2 mV	
Figure 3D3	Mixed-effects analysis	0 pA: LX↓ vs. LX↑	**0.0180**	(*t*) 2.375 (*df*) 415	0.14 ± 0.0 mV vs. 1.39 ± 0.7 mV	27 cells
		40 pA: LX↓ vs. LX↑	**<0.0001**	(*t*) 13.470 (*df*) 415	0.76 ± 0.2 mV vs. 7.99 ± 1.2 mV	
		80 pA: LX↓ vs. LX↑	**<0.0001**	(*t*) 11.300 (*df*) 415	5.18 ± 0.3 mV vs. 11.13 ± 1.0 mV	
		120 pA: LX↓ vs. LX↑	**<0.0001**	(*t*) 7.226 (*df*) 415	9.23 ± 0.5 mV vs. 13.03 ± 0.8 mV	
		160 pA: LX↓ vs. LX↑	**0.0045**	(*t*) 2.860 (*df*) 415	13.06 ± 0.5 mV vs. 14.57 ± 0.7 mV	
		200 pA: LX↓ vs. LX↑	0.4821	(*t*) 0.704 (*df*) 415	14.91 ± 0.5 mV vs. 15.28 ± 0.7 mV	
		240 pA: LX↓ vs. LX↑	0.6829	(*t*) 0.409 (*df*) 415	16.30 ± 0.7 mV vs. 16.09 ± 0.7 mV	
		280 pA: LX↓ vs. LX↑	0.9961	(*t*) 0.005 (*df*) 415	16.46 ± 0.7 mV vs. 16.46 ± 0.7 mV	
		320 pA			15.66 ± 0.9 mV vs. 15.66 ± 0.9 mV	
		0 pA: LIII vs. LX			0.00 ± 0.0 Hz vs. 0.00 ± 0.0 Hz	26 vs. 27 cells
		0 pA: LIII vs. LX			0.00 ± 0.0 Hz vs. 0.00 ± 0.0 Hz	26 vs. 27 cells
		0 pA: LIII vs. LX			0.00 ± 0.0 Hz vs. 0.00 ± 0.0 Hz	26 vs. 27 cells
		0 pA: LIII vs. LX			0.00 ± 0.0 Hz vs. 0.00 ± 0.0 Hz	26 vs. 27 cells
		0 pA: LIII vs. LX			0.00 ± 0.0 Hz vs. 0.00 ± 0.0 Hz	26 vs. 27 cells
		0 pA: LIII vs. LX			0.00 ± 0.0 Hz vs. 0.00 ± 0.0 Hz	26 vs. 27 cells
		0 pA: LIII vs. LX			0.00 ± 0.0 Hz vs. 0.00 ± 0.0 Hz	26 vs. 27 cells
		0 pA: LIII vs. LX			0.00 ± 0.0 Hz vs. 0.00 ± 0.0 Hz	26 vs. 27 cells
		0 pA: LIII vs. LX			0.00 ± 0.0 Hz vs. 0.00 ± 0.0 Hz	26 vs. 27 cells
		0 pA: LIII vs. LX			0.00 ± 0.0 Hz vs. 0.00 ± 0.0 Hz	26 vs. 27 cells
		40 pA: LIII vs. LX			0.00 ± 0.0 Hz vs. 0.00 ± 0.0 Hz	26 vs. 27 cells
		80 pA: LIII vs. LX	0.8280	(*t*) 0.217 (*df*) 1694	0.73 ± 0.0 Hz vs. 1.78 ± 1.0 Hz	26 vs. 27 cells
		120 pA: LIII vs. LX	0.6231	(*t*) 0.492 (*df*) 1694	13.10 ± 4.4 Hz vs. 10.73 ± 2.7 Hz	26 vs. 27 cells
		160 pA: LIII vs. LX	0.5397	(*t*) 0.613 (*df*) 1694	29.79 ± 6.6 Hz vs. 26.83 ± 3.7 Hz	26 vs. 27 cells
		200 pA: LIII vs. LX	0.1272	(*t*) 1.526 (*df*) 1694	47.75 ± 7.3 Hz vs. 40.40 ± 4.3 Hz	26 vs. 27 cells
		240 pA: LIII vs. LX	**0.0156**	(*t*) 2.421 (*df*) 1694	63.79 ± 7.8 Hz vs. 52.13 ± 4.2 Hz	26 vs. 27 cells
Figure 3E1	Mixed-effects analysis	280 pA: LIII vs. LX	**0.0009**	(*t*) 3.331 (*df*) 1694	77.81 ± 8.3 Hz vs. 61.76 ± 4.4 Hz	26 vs. 27 cells
		320 pA: LIII vs. LX	**<0.0001**	(*t*) 4.350 (*df*) 1694	88.65 ± 8.6 Hz vs. 67.69 ± 4.7 Hz	26 vs. 27 cells
		280 pA: LIII vs. LX	**0.0004**	(*t*) 3.543 (*df*) 1694	77.99 ± 8.3 Hz vs. 60.92 ± 4.4 Hz	26 vs. 27 cells
		240 pA: LIII vs. LX	**0.0181**	(*t*) 2.366 (*df*) 1694	64.00 ± 8.1 Hz vs. 52.60 ± 4.2 Hz	26 vs. 27 cells
		200 pA: LIII vs. LX	0.2112	(*t*) 1.251 (*df*) 1694	49.95 ± 7.4 Hz vs. 43.92 ± 3.9 Hz	26 vs. 27 cells
		160 pA: LIII vs. LX	0.6611	(*t*) 0.138 (*df*) 1694	36.57 ± 6.7 Hz vs. 34.46 ± 3.6 Hz	26 vs. 27 cells
		120 pA: LIII vs. LX	0.7107	(*t*) 0.371 (*df*) 1694	23.42 ± 5.5 Hz vs. 25.21 ± 3.2 Hz	26 vs. 27 cells
		80 pA: LIII vs. LX	0.6104	(*t*) 0.510 (*df*) 1694	12.71 ± 4.0 Hz vs. 15.17 ± 2.4 Hz	26 vs. 27 cells
		40 pA: LIII vs. LX	0.8125	(*t*) 0.237 (*df*) 1694	4.20 ± 1.8 Hz vs. 5.35 ± 1.2 Hz	26 vs. 27 cells
		0 pA: LIII vs. LX	0.9964	(*t*) 0.004 (*df*) 1694	0.48 ± 0.4 Hz vs. 0.50 ± 0.2 Hz	26 vs. 27 cells
		0 pA: LIII vs. LX	0.9769	(*t*) 0.029 (*df*) 1694	0.21 ± 0.2 Hz vs. 0.07 ± 0.0 Hz	26 vs. 27 cells
		0 pA: LIII vs. LX	0.9924	(*t*) 0.009 (*df*) 1694	0.06 ± 0.1 Hz vs. 0.02 ± 0.0 Hz	26 vs. 27 cells
		0 pA: LIII vs. LX			0.00 ± 0.0 Hz vs. 0.00 ± 0.0 Hz	26 vs. 27 cells
		0 pA: LIII vs. LX			0.00 ± 0.0 Hz vs. 0.00 ± 0.0 Hz	26 vs. 27 cells
		0 pA: LIII vs. LX			0.00 ± 0.0 Hz vs. 0.00 ± 0.0 Hz	26 vs. 27 cells
		0 pA: LIII vs. LX			0.00 ± 0.0 Hz vs. 0.00 ± 0.0 Hz	26 vs. 27 cells
		0 pA: LIII vs. LX			0.00 ± 0.0 Hz vs. 0.00 ± 0.0 Hz	26 vs. 27 cells
		0 pA: LIII vs. LX			0.00 ± 0.0 Hz vs. 0.00 ± 0.0 Hz	26 vs. 27 cells
		0 pA: LIII↓ vs. LIII↑			0.00 ± 0.0 Hz vs. 0.00 ± 0.0 Hz	
		0 pA: LIII↓ vs. LIII↑			0.00 ± 0.0 Hz vs. 0.00 ± 0.0 Hz	
		0 pA: LIII↓ vs. LIII↑			0.00 ± 0.0 Hz vs. 0.00 ± 0.0 Hz	
		0 pA: LIII↓ vs. LIII↑			0.00 ± 0.0 Hz vs. 0.00 ± 0.0 Hz	
		0 pA: LIII↓ vs. LIII↑			0.00 ± 0.0 Hz vs. 0.00 ± 0.0 Hz	
Figure 3E2	Mixed-effects analysis	0 pA: LIII↓ vs. LIII↑			0.00 ± 0.0 Hz vs. 0.00 ± 0.0 Hz	26 cells
		0 pA: LIII↓ vs. LIII↑	0.9879	(*t*) 0.015 (*df*) 416	0.00 ± 0.0 Hz vs. 0.06 ± 0.1 Hz	
		0 pA: LIII↓ vs. LIII↑	0.9610	(*t*) 0.049 (*df*) 416	0.00 ± 0.0 Hz vs. 0.21 ± 0.2 Hz	
		0 pA: LIII↓ vs. LIII↑	0.9098	(*t*) 0.113 (*df*) 416	0.00 ± 0.0 Hz vs. 0.48 ± 0.4 Hz	
		40 pA: LIII↓ vs. LIII↑	0.3224	(*t*) 0.991 (*df*) 416	0.00 ± 0.0 Hz vs. 4.2 ± 1.8 Hz	
		80 pA: LIII↓ vs. LIII↑	**0.0050**	(*t*) 2.824 (*df*) 416	0.73 ± 0.3 Hz vs. 12.71 ± 4.0 Hz	

		120 pA: LIII↓ vs. LIII↑	**0.0154**	(*t*) 2.432 (*df*) 416	13.10 ± 4.4 Hz vs. 23.42 ± 5.5 Hz	
		160 pA: LIII↓ vs. LIII↑	0.1106	(*t*) 1.599 (*df*) 416	29.79 ± 6.6 Hz vs. 36.57 ± 6.7 Hz	
		200 pA: LIII↓ vs. LIII↑	0.6045	(*t*) 0.518 (*df*) 416	47.75 ± 7.3 Hz vs. 49.95 ± 7.4 Hz	
		240 pA: LIII↓ vs. LIII↑	0.96410	(*t*) 0.049 (*df*) 416	63.79 ± 7.8 Hz vs. 64 ± 8.1 Hz	
		280 pA: LIII↓ vs. LIII↑	0.9667	(*t*) 0.042 (*df*) 416	77.81 ± 8.3 Hz vs. 77.99 ± 8.3 Hz	
		320 pA			88.65 ± 8.6 Hz	
Figure 3E3	Mixed-effects analysis	0 pA: LX↓ vs. LX↑			0.00 ± 0.0 Hz vs. 0.00 ± 0.0 Hz	27 cells
		0 pA: LX↓ vs. LX↑			0.00 ± 0.0 Hz vs. 0.00 ± 0.0 Hz	
		0 pA: LX↓ vs. LX↑			0.00 ± 0.0 Hz vs. 0.00 ± 0.0 Hz	
		0 pA: LX↓ vs. LX↑			0.00 ± 0.0 Hz vs. 0.00 ± 0.0 Hz	
		0 pA: LX↓ vs. LX↑			0.00 ± 0.0 Hz vs. 0.00 ± 0.0 Hz	
		0 pA: LX↓ vs. LX↑			0.00 ± 0.0 Hz vs. 0.00 ± 0.0 Hz	
		0 pA: LX↓ vs. LX↑	0.9902	(*t*) 0.012 (*df*) 415	0.00 ± 0.0 Hz vs. 0.02 ± 0.0 Hz	
		0 pA: LX↓ vs. LX↑	0.9641	(*t*) 0.045 (*df*) 415	0.00 ± 0.0 Hz vs. 0.07 ± 0.0 Hz	
		0 pA: LX↓ vs. LX↑	0.7389	(*t*) 0.334 (*df*) 415	0.00 ± 0.0 Hz vs. 0.50 ± 0.2 Hz	
		40 pA: LX↓ vs. LX↑	**0.0004**	(*t*) 3.550 (*df*) 415	0.00 ± 0.0 Hz vs. 5.35 ± 1.2 Hz	
		80 pA: LX↓ vs. LX↑	**<0.0001**	(*t*) 8.892 (*df*) 415	1.78 ± 1.0 Hz vs. 15.17 ± 2.4 Hz	
		120 pA: LX↓ vs. LX↑	**<0.0001**	(*t*) 9.613 (*df*) 415	10.73 ± 2.7 Hz vs. 25.21 ± 3.2 Hz	
		160 pA: LX↓ vs. LX↑	**<0.0001**	(*t*) 5.064 (*df*) 415	26.83 ± 3.7 Hz vs. 34.46 ± 3.6 Hz	
		200 pA: LX↓ vs. LX↑	**0.0198**	(*t*) 2.340 (*df*) 415	40.40 ± 4.3 Hz vs. 43.92 ± 3.9 Hz	
		240 pA: LX↓ vs. LX↑	0.7551	(*t*) 0.312 (*df*) 415	52.13 ± 4.2 Hz vs. 52.60 ± 4.2 Hz	
		280 pA: LX↓ vs. LX↑	0.5762	(*t*) 0.559 (*df*) 415	61.76 ± 4.4 Hz vs. 60.92 ± 4.4 Hz	
		320 pA			67.69 ± 4.7 Hz vs. 67.69 ± 4.7 Hz	

Table S4. Statistical data for in vitro whole cell patch electrophysiology in the juvenile mouse (P3-24), for action potential analysis. Descriptive values are represented as ± SEM. Abbreviations: LIII = Lobule III, LX = Lobule X, *df* = degrees of freedom, pA = picoampere, Ω = ohm, mV = millivolt, Vm = membrane voltage, msec = millisecond.

Related Figure	Test Applied	Group	*p*-Value	Population-Value	Descriptive Values	Population Size
Figure 4B	One-way ANOVA	P3-5: LIII vs. LX	0.5629	(*t*) 0.582 (*df*) 51	-21.89 ± 5.2 Vm vs. -25.91 ± 3.4 Vm	3 vs. 18 cells
		P15-17: LIII vs LX	0.7420	(*t*) 0.331 (*df*) 51	-38.35 ± 3.1 Vm vs. -36.76 ± 2.1 Vm	9 vs. 13 cells
		P22-24: LIII vs LX	0.8364	(*t*) 0.208 (*df*) 51	-38.51 ± 5.4 Vm vs. -39.79 ± 2.8 Vm	5 vs. 9 cells
		LIII: P3-5 vs. P15-17	**0.0303**	(*t*) 2.228 (*df*) 51	-21.89 ± 5.2 Vm vs. -38.35 ± 3.1 Vm	3 vs. 9 cells
		LIII: P3-5 vs. P22-24	**0.0451**	(*t*) 2.054 (*df*) 51	-21.89 ± 5.2 Vm vs. -38.51 ± 5.4 Vm	3 vs. 5 cells
		LIII: P15-17 vs. P22-24	0.7830	(*t*) 0.026 (*df*) 51	-38.35 ± 3.1 Vm vs. -38.51 ± 5.4 Vm	9 vs. 5 cells
		LX: P3-5 vs. P15-17	**0.0097**	(*t*) 2.095 (*df*) 51	-25.91 ± 3.4 Vm vs. -36.76 ± 2.1 Vm	18 vs. 13 cells
		LX: P3-5 vs. P22-24	**0.0034**	(*t*) 3.068 (*df*) 51	-25.91 ± 3.4 Vm vs. -39.79 ± 2.8 Vm	18 vs. 9 cells
		LX: P15-17 vs. P22-24	0.5302	(*t*) 0.632 (*df*) 51	-36.76 ± 2.1 Vm vs. -39.79 ± 2.8 Vm	13 vs. 9 cells
Figure 4C	One-way ANOVA	P3-5: LIII vs. LX	0.4313	(*t*) 0.793 (*df*) 51	61.24 ± 5.8 mV vs. 66.77 ± 2.2 mV	3 vs. 18 cells
		P15-17: LIII vs LX	0.8533	(*t*) 0.186 (*df*) 51	89.09 ± 4.7 mV vs. 89.99 ± 3.6 mV	9 vs. 13 cells
		P22-24: LIII vs LX	0.2000	(*t*) 1.298 (*df*) 51	89.30 ± 6.4 mV vs. 97.39 ± 2.3 mV	5 vs. 9 cells
		LIII: P3-5 vs. P15-17	**0.0005**	(*t*) 3.740 (*df*) 51	61.24 ± 5.8 mV vs. 89.09 ± 4.7 mV	3 vs. 9 cells
		LIII: P3-5 vs. P22-24	**0.0012**	(*t*) 3.439 (*df*) 51	61.24 ± 5.8 mV vs. 89.30 ± 6.4 mV	3 vs. 5 cells
		LIII: P15-17 vs. P22-24	0.9735	(*t*) 0.033 (*df*) 51	89.09 ± 4.7 mV vs. 89.30 ± 6.4 mV	9 vs. 5 cells
		LX: P3-5 vs. P15-17	**<0.0001**	(*t*) 5.712 (*df*) 51	66.77 ± 2.2 mV vs. 89.99 ± 3.6 mV	18 vs. 13 cells
		LX: P3-5 vs. P22-24	**<0.0001**	(*t*) 3.990 (*df*) 51	66.77 ± 2.2 mV vs. 97.39 ± 2.3 mV	18 vs. 9 cells
		LX: P15-17 vs. P22-24	0.1329	(*t*) 1.527 (*df*) 51	89.99 ± 3.6 mV vs. 97.39 ± 2.3 mV	13 vs. 9 cells
Figure 4D1	Kruskal–Wallis test	P3-5: LIII vs. LX	0.9698	(Z) 0.038	0.89 ± 0.17 msec vs. 0.85 ± 0.06 msec	3 vs. 18 cells
		P15-17: LIII vs LX	0.2963	(Z) 1.044	0.15 ± 0.01 msec vs. 0.18 ± 0.01 msec	9 vs. 13 cells
		P22-24: LIII vs LX	0.0705	(Z) 1.809	0.15 ± 0.01 msec vs. 0.22 ± 0.02 msec	5 vs. 9 cells
		LIII: P3-5 vs. P15-17	**0.0013**	(Z) 3.217	0.89 ± 0.17 msec vs. 0.15 ± 0.01 msec	3 vs. 9 cells
		LIII: P3-5 vs. P22-24	**0.0026**	(Z) 3.012	0.89 ± 0.17 msec vs. 0.15 ± 0.01 msec	3 vs. 5 cells
		LIII: P15-17 vs. P22-24	0.9220	(Z) 0.098	0.15 ± 0.01 msec vs. 0.15 ± 0.01 msec	9 vs. 5 cells
		LX: P3-5 vs. P15-17	**<0.0001**	(Z) 4.583	0.85 ± 0.06 msec vs. 0.18 ± 0.01 msec	18 vs. 13 cells
		LX: P3-5 vs. P22-24	**0.0043**	(Z) 2.858	0.85 ± 0.06 msec vs. 0.22 ± 0.02 msec	18 vs. 9 cells
		LX: P15-17 vs. P22-24	0.2475	(Z) 1.156	0.18 ± 0.01 msec vs. 0.22 ± 0.02 msec	13 vs. 9 cells
Figure 4D2	Kruskal–Wallis test	P3-5: LIII vs. LX	0.9436	(Z) 0.071	0.69 ± 0.16 msec vs. 0.65 ± 0.07 msec	3 vs. 18 cells
		P15-17: LIII vs LX	0.9530	(Z) 0.059	0.16 ± 0.02 msec vs. 0.16 ± 0.02 msec	9 vs. 13 cells
		P22-24: LIII vs LX	0.1674	(Z) 1.381	0.12 ± 0.00 msec vs. 0.15 ± 0.01 msec	5 vs. 9 cells
		LIII: P3-5 vs. P15-17	**0.0125**	(Z) 2.499	0.69 ± 0.16 msec vs. 0.16 ± 0.02 msec	3 vs. 9 cells
		LIII: P3-5 vs. P22-24	**0.0012**	(Z) 3.233	0.69 ± 0.16 msec vs. 0.12 ± 0.00 msec	3 vs. 5 cells
		LIII: P15-17 vs. P22-24	0.2124	(Z) 1.247	0.16 ± 0.02 msec vs. 0.12 ± 0.00 msec	9 vs. 5 cells
		LX: P3-5 vs. P15-17	**<0.0001**	(Z) 4.385	0.65 ± 0.07 msec vs. 0.16 ± 0.02 msec	18 vs. 13 cells
		LX: P3-5 vs. P22-24	**0.0002**	(Z) 3.790	0.65 ± 0.07 msec vs. 0.15 ± 0.01 msec	18 vs. 9 cells
		LX: P15-17 vs. P22-24	0.9099	(Z) 0.113	0.16 ± 0.02 msec vs. 0.15 ± 0.01 msec	13 vs. 9 cells
Figure 4D3	Brown-Forsythe and Welch ANOVA test	P3-5: LIII vs. LX	0.6002	(*t*) 0.575(*df*) 4	0.64 ± 0.12 msec vs. 0.72 ± 0.07 msec	3 vs. 18 cells
		P15-17: LIII vs LX	**0.0081**	(*t*) 3.006 (*df*) 17	0.14 ± 0.00 msec vs. 0.17 ± 0.01 msec	9 vs. 13 cells
		P22-24: LIII vs LX	**<0.0001**	(*t*) 6.076 (*df*)12	0.13 ± 0.01 msec vs. 0.24 ± 0.02 msec	5 vs. 9 cells
		LIII: P3-5 vs. P15-17	0.0547	(*t*) 4.089 (*df*) 2	0.64 ± 0.12 msec vs. 0.14 ± 0.00 msec	3 vs. 9 cells
		LIII: P3-5 vs. P22-24	0.0516	(*t*) 4.175 (*df*) 2	0.64 ± 0.12 msec vs. 0.13 ± 0.01 msec	3 vs. 5 cells
		LIII: P15-17 vs. P22-24	0.3282	(*t*) 1.068 (*df*) 6	0.14 ± 0.00 msec vs. 0.13 ± 0.01 msec	9 vs. 5 cells
		LX: P3-5 vs. P15-17	**<0.0001**	(*t*) 7.680 (*df*) 18	0.72 ± 0.07 msec vs. 0.17 ± 0.01 msec	18 vs. 13 cells
		LX: P3-5 vs. P22-24	**<0.0001**	(*t*) 6.626 (*df*) 19	0.72 ± 0.07 msec vs. 0.24 ± 0.02 msec	18 vs. 9 cells
		LX: P15-17 vs. P22-24	**0.0026**	(*t*) 3.635 (*df*) 14	0.17 ± 0.01 msec vs. 0.24 ± 0.02 msec	13 vs. 9 cells
Figure 4E	Kruskal–Wallis test	P3-5: LIII vs. LX	>0.9999	(Z) 0.000	0.00 ± 0.00 mV vs. 0.00 ± 0.00 mV	3 vs. 18 cells
		P15-17: LIII vs LX	0.3885	(Z) 0.862	8.24 ± 0.94 mV vs. 6.55 ± 1.02 mV	9 vs. 13 cells
		P22-24: LIII vs LX	0.6090	(Z) 0.512	11.06 ± 1.81 mV vs. 9.36 ± 1.28 mV	5 vs. 9 cells
		LIII: P3-5 vs. P15-17	**0.0116**	(Z) 2.524	0.00 ± 0.00 mV vs. 8.24 ± 0.94 mV	3 vs. 9 cells
		LIII: P3-5 vs. P22-24	**0.0026**	(Z) 3.013	0.00 ± 0.00 mV vs. 11.06 ± 1.81 mV	3 vs. 5 cells
		LIII: P15-17 vs. P22-24	0.3537	(Z) 0.928	8.24 ± 0.94 mV vs. 11.06 ± 1.81 mV	9 vs. 5 cells
		LX: P3-5 vs. P15-17	**0.0003**	(Z) 3.596	0.00 ± 0.00 mV vs. 6.55 ± 1.02 mV	18 vs. 13 cells
		LX: P3-5 vs. P22-24	**<0.0001**	(Z) 4.690	0.00 ± 0.00 mV vs. 9.36 ± 1.28 mV	18 vs. 9 cells
		LX: P15-17 vs. P22-24	0.1623	(Z) 1.397	6.55 ± 1.02 mV vs. 9.36 ± 1.28 mV	13 vs. 9 cells
Figure 4 S1A1	Kruskal–Wallis test	3-5: LIII vs. LX	0.7708	(Z) 0.291	889.3 ± 191.1 Ω vs. 729.6 ± 75.7 Ω	3 vs. 18 cells
		P15-17: LIII vs LX	0.0616	(Z) 1.869	66.76 ± 6.7 Ω vs. 108.5 ± 15.4 Ω	10 vs. 17 cells
		P22-24: LIII vs LX	0.4568	(Z) 0.744	77.41 ± 7.0 Ω vs. 102.4 ± 12.19 Ω	5 vs. 9 cells
		LIII: P3-5 vs. P15-17	**0.0003**	(Z) 3.635	889.3 ± 191.1 Ω vs. 66.76 ± 6.7 Ω	3 vs. 10 cells

		LIII: P3-5 vs. P22-24	**0.0059**	(*Z*) 2.753	889.3 ± 191.1 Ω vs. 77.41 ± 7.0 Ω	3 vs. 5 cells
		LIII: P15-17 vs. P22-24	0.4850	(*Z*) 0.698	66.76 ± 6.7 Ω vs. 77.41 ± 7.0 Ω	10 vs. 5 cells
		LX: P3-5 vs. P15-17	**<0.0001**	(*Z*) 4.335	729.6 ± 75.7 Ω vs. 108.5 ± 15.4 Ω	18 vs. 17 cells
		LX: P3-5 vs. P22-24	**0.0005**	(*Z*) 3.462	729.6 ± 75.7 Ω vs. 102.4 ± 12.19 Ω	18 vs. 9 cells
		LX: P15-17 vs. P22-24	0.8986	(*Z*) 0.127	108.5 ± 15.4 Ω vs. 102.4 ± 12.19 Ω	17 vs. 9 cells
Figure 4 S1A2	Kruskal–Wallis test	3-5: LIII vs. LX	0.7974	(*Z*) 0.257	874.7 ± 190.4 Ω vs. 716.3 ± 75.7 Ω	3 vs. 18 cells
		P15-17: LIII vs LX	0.0626	(*Z*) 1.862	52.33 ± 6.4 Ω vs. 92.74 ± 15.3 Ω	10 vs. 17 cells
		P22-24: LIII vs LX	0.4344	(*Z*) 0.782	63.50 ± 6.4 Ω vs. 89.32 ± 11.7 Ω	5 vs. 9 cells
		LIII: P3-5 vs. P15-17	**0.0003**	(*Z*) 3.632	874.7 ± 190.4 Ω vs. 52.33 ± 6.4 Ω	3 vs. 10 cells
		LIII: P3-5 vs. P22-24	**0.0070**	(*Z*) 2.697	874.7 ± 190.4 Ω vs. 63.50 ± 6.4 Ω	3 vs. 5 cells
		LIII: P15-17 vs. P22-24	0.4418	(*Z*) 0.769	52.33 ± 6.4 Ω vs. 63.50 ± 6.4 Ω	10 vs. 5 cells
		LX: P3-5 vs. P15-17	**<0.0001**	(*Z*) 5.656	716.3 ± 75.7 Ω vs. 92.74 ± 15.3 Ω	18 vs. 17 cells
		LX: P3-5 vs. P22-24	**0.0008**	(*Z*) 3.364	716.3 ± 75.7 Ω vs. 89.32 ± 11.7 Ω	18 vs. 9 cells
		LX: P15-17 vs. P22-24	0.7799	(*Z*) 0.280	92.74 ± 15.3 Ω vs. 89.32 ± 11.7 Ω	17 vs. 9 cells
Figure 4 S1A3	One-way ANOVA	3-5: LIII vs. LX	0.3858	(*t*) 0.874 (*df*) 56	14.62 ±1.5 Ω vs. 13.25 ± 0.6 Ω	3 vs. 18 cells
		P15-17: LIII vs LX	0.2043	(*t*)1.284 (*df*) 56	14.43 ± 0.8 Ω vs. 15.71 ± 0.5 Ω	10 vs. 17 cells
		P22-24: LIII vs LX	0.5386	(*t*) 0.619 (*df*) 56	13.91 ± 1.4 Ω vs. 13.04 ± 1.1 Ω	5 vs. 9 cells
		LIII: P3-5 vs. P15-17	0.9072	(*t*) 0.117 (*df*) 56	14.62 ±1.5 Ω vs. 14.43 ± 0.8 Ω	3 vs. 10 cells
		LIII: P3-5 vs. P22-24	0.7008	(*t*) 0.386 (*df*) 56	14.62 ±1.5 Ω vs. 13.91 ± 1.4 Ω	3 vs. 5 cells
		LIII: P15-17 vs. P22-24	0.7097	(*t*) 0.374 (*df*) 56	14.43 ± 0.8 Ω vs. 13.91 ± 1.4 Ω	10 vs. 5 cells
		LX: P3-5 vs. P15-17	**0.0054**	(*t*) 2.897 (*df*) 56	13.25 ± 0.6 Ω vs. 15.71 ± 0.5 Ω	18 vs. 17 cells
		LX: P3-5 vs. P22-24	0.8414	(*t*) 0.201 (*df*) 56	13.25 ± 0.6 Ω vs. 13.04 ± 1.1 Ω	18 vs. 9 cells
		LX: P15-17 vs. P22-24	**0.0127**	(*t*) 2.576 (*df*) 56	15.71 ± 0.5 Ω vs. 13.04 ± 1.1 Ω	17 vs. 9 cells
Figure 4 S1B	One-way ANOVA	3-5: LIII vs. LX	0.3212	(*t*) 1.002 (*df*) 51	12.06 ± 8.1 Vm vs. 18.07 ± 1.8 Vm	3 vs. 18 cells
		P15-17: LIII vs LX	0.9179	(*t*) 0.104 (*df*) 51	36.79 ± 3.6 Vm vs. 37.22 ± 2.7 Vm	9 vs. 13 cells
		P22-24: LIII vs LX	**0.0180**	(*t*) 2.444 (*df*) 51	36.16 ± 4.6 Vm vs. 49.26 ± 3.4 Vm	5 vs. 9 cells
		LIII: P3-5 vs. P15-17	**0.0003**	(*t*) 3.858 (*df*) 51	12.06 ± 8.1 Vm vs. 36.79 ± 3.6 Vm	3 vs. 9 cells
		LIII: P3-5 vs. P22-24	**0.0012**	(*t*) 3.431 (*df*) 51	12.06 ± 8.1 Vm vs. 36.16 ± 4.6 Vm	3 vs. 5 cells
		LIII: P15-17 vs. P22-24	0.9062	(*t*) 0.118 (*df*) 51	36.79 ± 3.6 Vm vs. 36.16 ± 4.6 Vm	9 vs. 5 cells
		LX: P3-5 vs. P15-17	**<0.0001**	(*t*) 5.473 (*df*) 51	18.07 ± 1.8 Vm vs. 37.22 ± 2.7 Vm	18 vs. 13 cells
		LX: P3-5 vs. P22-24	**<0.0001**	(*t*) 7.947 (*df*) 51	18.07 ± 1.8 Vm vs. 49.26 ± 3.4 Vm	18 vs. 9 cells
		LX: P15-17 vs. P22-24	**0.0057**	(*t*) 2.888 (*df*) 51	37.22 ± 2.7 Vm vs. 49.26 ± 3.4 Vm	13 vs. 9 cells
Figure 4 S1C	Brown-Forsythe and Welch ANOVA test	3-5: LIII vs. LX	>0.9999	(*t*) 0.248 (*df*) 3	32.00 ± 6.7 pA vs. 30.17 ± 3.2 pA	3 vs. 18 cells
		P15-17: LIII vs LX	0.9287	(*t*) 1.332 (*df*) 17	83.67 ± 16.2 pA vs. 56.08 ± 12.9 pA	9 vs. 13 cells
		P22-24: LIII vs LX	0.9882	(*t*) 1.020 (*df*) 12	60.80 ± 21.0 pA vs. 93.89 ± 24.7 pA	5 vs. 9 cells
		LIII: P3-5 vs. P15-17	0.1519	(*t*) 2.955 (*df*) 10	32.00 ± 6.7 pA vs. 83.67 ± 16.2 pA	3 vs. 9 cells
		LIII: P3-5 vs. P22-24	0.9049	(*t*) 1.306 (*df*) 5	32.00 ± 6.7 pA vs. 60.80 ± 21.0 pA	3 vs. 5 cells
		LIII: P15-17 vs. P22-24	0.9964	(*t*) 0.862 (*df*) 9	83.67 ± 16.2 pA vs. 60.80 ± 21.0 pA	9 vs. 5 cells
		LX: P3-5 vs. P15-17	0.1071	(*t*) 1.943 (*df*) 14	30.17 ± 3.2 pA vs. 56.08 ± 12.9 pA	18 vs. 13 cells
		LX: P3-5 vs. P22-24	0.2931	(*t*) 2.559 (*df*) 8	30.17 ± 3.2 pA vs. 93.89 ± 24.7 pA	18 vs. 9 cells
		LX: P15-17 vs. P22-24	0.9124	(*t*) 1.356 (*df*) 12	56.08 ± 12.9 pA vs. 93.89 ± 24.7 pA	13 vs. 9 cells
Figure 4 S1D	Kruskal–Wallis test	3-5: LIII vs. LX	-	-	-	3 vs. 18 cells
		P15-17: LIII vs LX	0.2252	(*Z*) 1.213	0.09 ± 0.01 msec vs. 0.14 ± 0.03 msec	9 vs. 13 cells
		P22-24: LIII vs LX	**0.0196**	(*Z*) 2.333	0.12 ± 0.02 msec vs. 0.29 ± 0.07 msec	5 vs. 9 cells
		LIII: P15-17 vs. P22-24	0.3718	(*Z*) 0.893	0.09 ± 0.01 msec vs. 0.12 ± 0.02 msec	9 vs. 5 cells
		LX: P15-17 vs. P22-24	**0.0046**	(*Z*) 2.834	0.14 ± 0.03 msec vs. 0.29 ± 0.07 msec	13 vs. 9 cells

Table S5. Statistical data for in vitro whole cell patch electrophysiology in the juvenile mouse (P15-17), for negative and positive current step analysis. Descriptive values are represented as ± SEM. Abbreviations: LIII = Lobule III, LX = Lobule X, *df* = degrees of freedom, Hz = hertz, mV = millivolt.

Related Figure	Test Applied	Group	*p*-Value	Population-Value	Descriptive Values	Population Size
Figure 5A1	Mixed-effects analysis	-0 pA: LIII vs. LX			0.00 ± 0.0 mV vs. 0.00 ± 0.0 mV	9 vs. 14 cells
		-40 pA: LIII vs. LX	0.0515	(*t*) 1.956 (*df*) 258	-7.51 ± 2.3 mV vs. -2.59 ± 0.9 mV	9 vs. 14 cells
		-80 pA: LIII vs. LX	0.5948	(*t*) 0.533 (*df*) 258	-13.63 ± 1.2 mV vs. -12.29 ± 1.8 mV	9 vs. 14 cells
		-120 pA: LIII vs. LX	0.4763	(*t*) 0.713 (*df*) 258	-15.76 ± 1.3 mV vs. -17.55 ± 1.6 mV	9 vs. 14 cells
		-160 pA: LIII vs. LX	0.4571	(*t*) 0.745 (*df*) 258	-17.76 ± 1.3 mV vs. -19.44 ± 1.6 mV	9 vs. 14 cells
		-200 pA: LIII vs. LX	0.1532	(*t*) 1.432 (*df*) 258	-19.44 ± 1.4 mV vs. -23.04 ± 1.7 mV	9 vs. 14 cells
		-240 pA: LIII vs. LX	0.0743	(*t*) 1.792 (*df*) 258	-20.95 ± 1.5 mV vs. -25.45 ± 1.6 mV	9 vs. 14 cells
		-280 pA: LIII vs. LX	0.0574	(*t*) 1.909 (*df*) 258	-22.79 ± 1.6 mV vs. -27.59 ± 1.5 mV	9 vs. 14 cells
		-320 pA: LIII vs. LX	**0.0291**	(*t*) 2.195 (*df*) 258	-24.38 ± 1.6 mV vs. -29.89 ± 1.6 mV	9 vs. 14 cells
		-360 pA: LIII vs. LX	**0.0211**	(*t*) 2.321 (*df*) 258	-26.03 ± 1.7 mV vs. -31.86 ± 1.6 mV	9 vs. 14 cells
		-400 pA: LIII vs. LX	**0.0083**	(*t*) 2.661 (*df*) 258	-27.60 ± 1.9 mV vs. -34.29 ± 1.7 mV	9 vs. 14 cells
		-440 pA: LIII vs. LX	**0.0067**	(*t*) 2.731 (*df*) 258	-27.12 ± 5.4 mV vs. -41.42 ± 5.0 mV	2 vs. 3 cells
		-480 pA: LIII vs. LX	**0.0013**	(*t*) 3.245 (*df*) 258	-28.59 ± 6.0 mV vs. -45.05 ± 5.4 mV	2 vs. 3 cells
		-520 pA: LIII vs. LX	**0.0002**	(*t*) 3.751 (*df*) 258	-30.03 ± 6.7 mV vs. -48.62 ± 5.8 mV	2 vs. 3 cells
		-560 pA: LIII vs. LX	**<0.0001**	(*t*) 4.303 (*df*) 258	-31.63 ± 7.3 mV vs. -52.55 ± 6.4 mV	2 vs. 3 cells
		-600 pA: LIII vs. LX	**<0.0001**	(*t*) 4.945 (*df*) 258	-32.60 ± 7.0 mV vs. -56.22 ± 6.7 mV	2 vs. 3 cells
		-640 pA: LIII vs. LX	**<0.0001**	(*t*) 5.282 (*df*) 258	-35.08 ± 8.4 mV vs. -60.12 ± 7.2 mV	2 vs. 3 cells
		-680 pA: LIII vs. LX	**<0.0001**	(*t*) 5.654 (*df*) 258	-37.07 ± 9.2 mV vs. -63.68 ± 7.8 mV	2 vs. 3 cells
		-720 pA: LIII vs. LX	**<0.0001**	(*t*) 5.785 (*df*) 258	-38.90 ± 9.7 mV vs. -66.06 ± 7.4 mV	2 vs. 3 cells
		-760 pA: LIII vs. LX	**<0.0001**	(*t*) 5.933 (*df*) 258	-40.67 ± 10.3 mV vs. -68.45 ± 7.4 mV	2 vs. 3 cells
Figure 5A2	Mixed-effects analysis	-0 pA: LIII vs. LX	0.7509	(*t*) 0.318 (*df*) 420	48.11 ± 10.0 Hz vs. 46.79 ± 7.3 Hz	9 vs. 14 cells
		-40 pA: LIII vs. LX	**0.0019**	(*t*) 3.129 (*df*) 420	16.44 ± 8.3 Hz vs. 29.50 ± 6.5 Hz	9 vs. 14 cells
		-80 pA: LIII vs. LX	**0.0255**	(*t*) 2.242 (*df*) 420	0.00 ± 0.0 Hz vs. 9.36 ± 5.5 Hz	9 vs. 14 cells
		-120 pA: LIII vs. LX	0.3131	(*t*) 1.010 (*df*) 420	0.00 ± 0.0 Hz vs. 4.21 ± 4.2 Hz	9 vs. 14 cells
		-160 pA: LIII vs. LX	0.4517	(*t*) 0.753 (*df*) 420	0.00 ± 0.0 Hz vs. 3.14 ± 3.1 Hz	9 vs. 14 cells
		-200 pA: LIII vs. LX	>0.9999	(*t*) 0.000 (*df*) 420	0.00 ± 0.0 Hz vs. 0.00 ± 0.0 Hz	9 vs. 14 cells
		-240 pA: LIII vs. LX	>0.9999	(*t*) 0.000 (*df*) 420	0.00 ± 0.0 Hz vs. 0.00 ± 0.0 Hz	9 vs. 14 cells
		-280 pA: LIII vs. LX	>0.9999	(*t*) 0.000 (*df*) 420	0.00 ± 0.0 Hz vs. 0.00 ± 0.0 Hz	9 vs. 14 cells
		-320 pA: LIII vs. LX	>0.9999	(*t*) 0.000 (*df*) 420	0.00 ± 0.0 Hz vs. 0.00 ± 0.0 Hz	9 vs. 14 cells
		-360 pA: LIII vs. LX	>0.9999	(*t*) 0.000 (*df*) 420	0.00 ± 0.0 Hz vs. 0.00 ± 0.0 Hz	9 vs. 14 cells
		-400 pA: LIII vs. LX	>0.9999	(*t*) 0.000 (*df*) 420	0.00 ± 0.0 Hz vs. 0.00 ± 0.0 Hz	9 vs. 14 cells
		-440 pA: LIII vs. LX	>0.9999	(*t*) 0.000 (*df*) 420	0.00 ± 0.0 Hz vs. 0.00 ± 0.0 Hz	2 vs. 3 cells
		-480 pA: LIII vs. LX	>0.9999	(*t*) 0.000 (*df*) 420	0.00 ± 0.0 Hz vs. 0.00 ± 0.0 Hz	2 vs. 3 cells
		-520 pA: LIII vs. LX	>0.9999	(*t*) 0.000 (*df*) 420	0.00 ± 0.0 Hz vs. 0.00 ± 0.0 Hz	2 vs. 3 cells
		-560 pA: LIII vs. LX	>0.9999	(*t*) 0.000 (*df*) 420	0.00 ± 0.0 Hz vs. 0.00 ± 0.0 Hz	2 vs. 3 cells
		-600 pA: LIII vs. LX	>0.9999	(*t*) 0.000 (*df*) 420	0.00 ± 0.0 Hz vs. 0.00 ± 0.0 Hz	2 vs. 3 cells
		-640 pA: LIII vs. LX	>0.9999	(*t*) 0.000 (*df*) 420	0.00 ± 0.0 Hz vs. 0.00 ± 0.0 Hz	2 vs. 3 cells
		-680 pA: LIII vs. LX	>0.9999	(*t*) 0.000 (*df*) 420	0.00 ± 0.0 Hz vs. 0.00 ± 0.0 Hz	2 vs. 3 cells
		-720 pA: LIII vs. LX	>0.9999	(*t*) 0.000 (*df*) 420	0.00 ± 0.0 Hz vs. 0.00 ± 0.0 Hz	2 vs. 3 cells
		-760 pA: LIII vs. LX	>0.9999	(*t*) 0.000 (*df*) 420	0.00 ± 0.0 Hz vs. 0.00 ± 0.0 Hz	2 vs. 3 cells
Figure 5B1	Mixed-effects analysis	0 pA: LIII vs. LX			0.00 ± 0.0 mV vs. 0.00 ± 0.0 mV	8 vs. 12 cells
		40 pA: LIII vs. LX	0.9486	(*t*) 0.065 (*df*) 360	3.83 ± 1.8 mV vs. 3.68 ± 0.8 mV	8 vs. 12 cells
		80 pA: LIII vs. LX	0.1793	(*t*) 1.345 (*df*) 360	6.28 ± 1.9 mV vs. 9.37 ± 1.2 mV	8 vs. 12 cells
		120 pA: LIII vs. LX	0.0522	(*t*) 1.948 (*df*) 360	9.41 ± 1.7 mV vs. 13.89 ± 1.4 mV	8 vs. 12 cells
		160 pA: LIII vs. LX	0.3968	(*t*) 0.8484 (*df*) 360	13.16 ± 1.5 mV vs. 15.11 ± 1.3 mV	8 vs. 12 cells
		200 pA: LIII vs. LX	0.5652	(*t*) 0.5757 (*df*) 360	15.03 ± 1.4 mV vs. 16.36 ± 1.3 mV	8 vs. 12 cells
		240 pA: LIII vs. LX	0.5147	(*t*) 0.6522 (*df*) 360	16.07 ± 1.5 mV vs. 17.57 ± 1.3 mV	8 vs. 12 cells
		280 pA: LIII vs. LX	0.4095	(*t*) 0.8257 (*df*) 360	16.81 ± 1.5 mV vs. 18.71 ± 1.1 mV	8 vs. 12 cells
		320 pA: LIII vs. LX	0.4746	(*t*) 0.7158 (*df*) 360	17.74 ± 1.5 mV vs. 19.39 ± 1.1 mV	8 vs. 12 cells
		360 pA: LIII vs. LX	0.7415	(*t*) 0.3302 (*df*) 360	18.70 ± 1.5 mV vs. 19.46 ± 1.4 mV	8 vs. 12 cells
		400 pA: LIII vs. LX	0.9070	(*t*) 0.1170 (*df*) 360	19.80 ± 1.4 mV vs. 20.07 ± 1.4 mV	8 vs. 12 cells
		440 pA: LIII vs. LX	0.5091	(*t*) 0.6609 (*df*) 360	22.33 ± 2.5 mV vs. 20.82 ± 1.3 mV	8 vs. 12 cells
		480 pA: LIII vs. LX	0.5194	(*t*) 0.6449 (*df*) 360	22.93 ± 2.6 mV vs. 21.45 ± 1.2 mV	8 vs. 12 cells
		520 pA: LIII vs. LX	0.5967	(*t*) 0.5296 (*df*) 360	23.85 ± 2.7 mV vs. 22.63 ± 1.3 mV	8 vs. 12 cells
		560 pA: LIII vs. LX	0.8822	(*t*) 0.1483 (*df*) 360	24.91 ± 2.8 mV vs. 24.56 ± 1.9 mV	8 vs. 12 cells
		600 pA: LIII vs. LX	0.6381	(*t*) 0.4707 (*df*) 360	26.00 ± 2.8 mV vs. 24.92 ± 1.1 mV	8 vs. 12 cells
		640 pA: LIII vs. LX	0.6201	(*t*) 0.4961 (*df*) 360	26.74 ± 2.7 mV vs. 25.60 ± 1.1 mV	8 vs. 12 cells
		680 pA: LIII vs. LX	0.6735	(*t*) 0.4217 (*df*) 360	27.55 ± 2.8 mV vs. 26.58 ± 1.1 mV	8 vs. 12 cells

		720 pA: LIII vs. LX	0.6445	(*t*) 0.4618 (*df*) 360	28.47 ± 2.8 mV vs. 27.41 ± 1.2 mV	8 vs. 12 cells
		760 pA: LIII vs. LX	0.5222	(*t*) 0.6404 (*df*) 360	29.22 ± 2.9 mV vs. 27.74 ± 1.1 mV	8 vs. 12 cells
Figure 5B2	Two-way ANOVA	0 pA: LIII vs. LX	>0.9999	(*t*) 0.000 (*df*) 360	0.00 ± 0.0 Hz vs. 0.00 ± 0.0 Hz	8 vs. 12 cells
		40 pA: LIII vs. LX	0.7705	(*t*) 0.292 (*df*) 360	6.88 ± 6.9 Hz vs. 1.00 ± 1.0 Hz	8 vs. 12 cells
		80 pA: LIII vs. LX	0.8898	(*t*) 0.139 (*df*) 360	18.38 ± 11.8 Hz vs. 15.58 ± 5.6 Hz	8 vs. 12 cells
		120 pA: LIII vs. LX	0.7626	(*t*) 0.302 (*df*) 360	29.00 ± 14.1 Hz vs. 35.08 ± 6.9 Hz	8 vs. 12 cells
		160 pA: LIII vs. LX	0.8979	(*t*) 0.128 (*df*) 360	41.50 ± 15.1 Hz vs. 44.08 ± 7.6 Hz	8 vs. 12 cells
		200 pA: LIII vs. LX	0.8930	(*t*) 0.135 (*df*) 360	54.13 ± 15.8 Hz vs. 51.42 ± 8.0 Hz	8 vs. 12 cells
		240 pA: LIII vs. LX	0.7563	(*t*) 0.311 (*df*) 360	64.00 ± 16.7 Hz vs. 57.75 ± 8.4 Hz	8 vs. 12 cells
		280 pA: LIII vs. LX	0.6731	(*t*) 0.422 (*df*) 360	73.50 ± 18.2 Hz vs. 65.00 ± 8.8 Hz	8 vs. 12 cells
		320 pA: LIII vs. LX	0.6343	(*t*) 0.476 (*df*) 360	82.00 ± 19.1 Hz vs. 72.42 ± 8.4 Hz	8 vs. 12 cells
		360 pA: LIII vs. LX	0.5542	(*t*) 0.592 (*df*) 360	90.75 ± 19.8 Hz vs. 78.83 ± 8.7 Hz	8 vs. 12 cells
		400 pA: LIII vs. LX	0.4188	(*t*) 0.809 (*df*) 360	99.88 ± 18.3 Hz vs. 83.58 ± 9.0 Hz	8 vs. 12 cells
		440 pA: LIII vs. LX	0.5350	(*t*) 0.621 (*df*) 360	100.75 ± 16.1 Hz vs. 88.25 ± 9.3 Hz	8 vs. 12 cells
		480 pA: LIII vs. LX	0.8458	(*t*) 0.195 (*df*) 360	97.50 ± 16.6 Hz vs. 93.58 ± 9.7 Hz	8 vs. 12 cells
		520 pA: LIII vs. LX	0.7438	(*t*) 0.327 (*df*) 360	91.50 ± 24.3 Hz vs. 98.08 ± 10.1 Hz	8 vs. 12 cells
		560 pA: LIII vs. LX	0.9571	(*t*) 0.054 (*df*) 360	97.25 ± 25.6 Hz vs. 98.33 ± 10.9 Hz	8 vs. 12 cells
		600 pA: LIII vs. LX	0.8963	(*t*) 0.130 (*df*) 360	102.88 ± 27.1 Hz vs. 105.50 ± 10.5 Hz	8 vs. 12 cells
		640 pA: LIII vs. LX	0.9291	(*t*) 0.089 (*df*) 360	108.38 ± 28.6 Hz vs. 110.17 ± 10.7 Hz	8 vs. 12 cells
		680 pA: LIII vs. LX	0.9357	(*t*) 0.081 (*df*) 360	112.38 ± 30.1 Hz vs. 114.00 ± 10.8 Hz	8 vs. 12 cells
		720 pA: LIII vs. LX	0.9127	(*t*) 0.110 (*df*) 360	115.13 ± 31.4 Hz vs. 117.33 ± 11.0 Hz	8 vs. 12 cells
		760 pA: LIII vs. LX	0.9160	(*t*) 0.106 (*df*) 360	117.88 ± 32.1 Hz vs. 120.00 ± 11.0 Hz	8 vs. 12 cells
Figure 5 S1A	Mixed-effects analysis	-0 pA: LIII vs. LX			0.00 ± 0.0 mV vs. 0.00 ± 0.0 mV	9 vs. 14 cells
		-40 pA: LIII vs. LX	0.1468	(*t*) 1.541 (*df*) 13	2.71 ± 1.0 mV vs. 0.98 ± 0.6 mV	9 vs. 14 cells
		-80 pA: LIII vs. LX	0.4077	(*t*) 0.845 (*df*) 21	4.36 ± 0.5 mV vs. 3.60 ± 0.7 mV	9 vs. 14 cells
		-120 pA: LIII vs. LX	0.9351	(*t*) 0.082 (*df*) 20	6.07 ± 0.8 mV vs. 5.98 ± 0.8 mV	9 vs. 14 cells
		-160 pA: LIII vs. LX	0.8841	(*t*) 0.148 (*df*) 14	7.25 ± 0.8 mV vs. 7.07 ± 0.9 mV	9 vs. 14 cells
		-200 pA: LIII vs. LX	0.6238	(*t*) 0.501 (*df*) 20	8.17 ± 0.9 mV vs. 8.70 ± 0.6 mV	9 vs. 14 cells
		-240 pA: LIII vs. LX	0.4813	(*t*) 0.717 (*df*) 20	9.14 ± 0.8 mV vs. 10.00 ± 0.9 mV	9 vs. 14 cells
		-280 pA: LIII vs. LX	0.3542	(*t*) 0.949 (*df*) 19	10.01 ± 0.9 mV vs. 11.26 ± 0.9 mV	9 vs. 14 cells
		-320 pA: LIII vs. LX	0.1522	(*t*) 1.490 (*df*) 18	10.27 ± 1.0 mV vs. 12.24 ± 0.9 mV	9 vs. 14 cells
		-360 pA: LIII vs. LX	0.3530	(*t*) 0.954 (*df*) 17	11.74 ± 1.1 mV vs. 13.09 ± 0.9 mV	9 vs. 14 cells
		-400 pA: LIII vs. LX	0.296	(*t*) 1.079 (*df*) 3	12.31 ± 1.2 mV vs. 13.89 ± 0.9 mV	9 vs. 14 cells
		-440 pA: LIII vs. LX	0.2426	(*t*) 1.472 (*df*) 3	13.79 ± 1.6 mV vs. 17.20 ± 1.7 mV	2 vs. 3 cells
		-480 pA: LIII vs. LX	0.2694	(*t*) 1.362 (*df*) 3	14.41 ± 1.3 mV vs. 17.60 ± 1.5 mV	2 vs. 3 cells
		-520 pA: LIII vs. LX	0.4793	(*t*) 0.823 (*df*) 3	14.94 ± 1.6 mV vs. 16.79 ± 1.6 mV	2 vs. 3 cells
		-560 pA: LIII vs. LX	0.4976	(*t*) 0.783 (*df*) 2	15.28 ± 1.4 mV vs. 16.87 ± 1.4 mV	2 vs. 3 cells
		-600 pA: LIII vs. LX	0.9716	(*t*) 0.040 (*df*) 3	16.42 ± 2.0 mV vs. 16.32 ± 1.4 mV	2 vs. 3 cells
		-640 pA: LIII vs. LX	0.9944	(*t*) 0.008 (*df*) 2	16.25 ± 0.9 mV vs. 16.24 ± 1.5 mV	2 vs. 3 cells
		-680 pA: LIII vs. LX	0.5381	(*t*) 0.719 (*df*) 2	16.80 ± 0.6 mV vs. 15.19 ± 2.1 mV	2 vs. 3 cells
		-720 pA: LIII vs. LX	0.8118	(*t*) 0.301 (*df*) 1	16.88 ± 0.1 mV vs. 17.11 ± 0.8 mV	2 vs. 3 cells
		-760 pA: LIII vs. LX	0.4779	(*t*) 1.070 (*df*) 1	17.80 ± 0.1 mV vs. 16.60 ± 1.1 mV	2 vs. 3 cells
Figure 5 S1B	Two-way ANOVA	0 pA: LIII vs. LX	>0.9999	(*t*) 0.000 (*df*) 380	0.00 ± 0.0 mV vs. 0.00 ± 0.0 mV	8 vs. 12 cells
		40 pA: LIII vs. LX	0.6175	(*t*) 0.500 (*df*) 380	1.18 ± 1.2 mV vs. 0.55 ± 0.6 mV	8 vs. 12 cells
		80 pA: LIII vs. LX	0.7990	(*t*) 0.255 (*df*) 380	2.67 ± 1.2 mV vs. 2.99 ± 0.8 mV	8 vs. 12 cells
		120 pA: LIII vs. LX	0.6746	(*t*) 0.420 (*df*) 380	3.71 ± 1.5 mV vs. 4.24 ± 0.7 mV	8 vs. 12 cells
		160 pA: LIII vs. LX	0.6921	(*t*) 0.396 (*df*) 380	5.66 ± 1.4 mV vs. 5.16 ± 0.8 mV	8 vs. 12 cells
		200 pA: LIII vs. LX	0.2857	(*t*) 1.069 (*df*) 380	6.73 ± 1.2 mV vs. 5.39 ± 0.7 mV	8 vs. 12 cells
		240 pA: LIII vs. LX	0.2216	(*t*) 1.224 (*df*) 380	6.98 ± 1.2 mV vs. 5.44 ± 0.8 mV	8 vs. 12 cells
		280 pA: LIII vs. LX	0.2847	(*t*) 1.071 (*df*) 380	7.17 ± 1.3 mV vs. 5.82 ± 0.6 mV	8 vs. 12 cells
		320 pA: LIII vs. LX	0.3375	(*t*) 0.960 (*df*) 380	7.47 ± 1.3 mV vs. 6.26 ± 0.7 mV	8 vs. 12 cells
		360 pA: LIII vs. LX	**0.0348**	(*t*) 2.118 (*df*) 380	8.86 ± 0.7 mV vs. 6.20 ± 0.6 mV	8 vs. 12 cells
		400 pA: LIII vs. LX	**0.0405**	(*t*) 2.055 (*df*) 380	9.08 ± 0.7 mV vs. 6.49 ± 0.7 mV	8 vs. 12 cells
		440 pA: LIII vs. LX	0.0504	(*t*) 1.963 (*df*) 380	9.30 ± 0.8 mV vs. 6.83 ± 0.7 mV	8 vs. 12 cells
		480 pA: LIII vs. LX	0.0641	(*t*) 1.857 (*df*) 380	9.23 ± 0.8 mV vs. 6.90 ± 0.8 mV	8 vs. 12 cells
		520 pA: LIII vs. LX	**0.0371**	(*t*) 2.092 (*df*) 380	9.42 ± 0.8 mV vs. 6.79 ± 0.8 mV	8 vs. 12 cells
		560 pA: LIII vs. LX	**0.0455**	(*t*) 2.007 (*df*) 380	9.60 ± 0.9 mV vs. 7.08 ± 0.8 mV	8 vs. 12 cells
		600 pA: LIII vs. LX	0.0641	(*t*) 1.857 (*df*) 380	9.67 ± 1.1 mV vs. 7.34 ± 0.8 mV	8 vs. 12 cells
		640 pA: LIII vs. LX	0.1420	(*t*) 1.472 (*df*) 380	9.51 ± 1.2 mV vs. 7.66 ± 0.8 mV	8 vs. 12 cells
		680 pA: LIII vs. LX	0.1660	(*t*) 1.388 (*df*) 380	9.47 ± 1.3 mV vs. 7.72 ± 0.8 mV	8 vs. 12 cells
		720 pA: LIII vs. LX	0.1552	(*t*) 1.424 (*df*) 380	9.62 ± 1.3 mV vs. 7.83 ± 0.8 mV	8 vs. 12 cells
		760 pA: LIII vs. LX	0.1468	(*t*) 1.454 (*df*) 380	9.65 ± 1.3 mV vs. 7.82 ± 0.7 mV	8 vs. 12 cells

Table S6. Statistical data for in vitro whole cell patch electrophysiology in the juvenile mouse (P15-17), for negative and positive current ramp analysis. Descriptive values are represented as ± SEM. Abbreviations: LIII = Lobule III, LIII↓ = Lobule III downphase at the current ramp, LIII↑ = Lobule III upphase at the current ramp, LX = Lobule X, LX↓ = Lobule X downphase at the current ramp, LX↑ = Lobule X upphase at the current ramp, *df* = degrees of freedom, Hz = hertz, mV = millivolt.

Related Figure	Test Applied	Group	*p*-Value	Population-Value	Descriptive Values	Population Size
		-0 pA: LIII vs. LX			0.00 ± 0.0 mV vs. 0.00 ± 0.0 mV	9 vs. 15 cells
		-0 pA: LIII vs. LX	0.6918	(*t*) 0.048 (*df*) 748	0.01 ± 0.1 mV vs. -0.07 ± 0.0 mV	9 vs. 15 cells
		-0 pA: LIII vs. LX	0.9354	(*t*) 0.081 (*df*) 748	0.11 ± 0.1 mV vs. -0.02 ± 0.0 mV	9 vs. 15 cells
		-0 pA: LIII vs. LX	0.9920	(*t*) 0.010 (*df*) 748	0.02 ± 0.1 mV vs. 0.00 ± 0.0 mV	9 vs. 15 cells
		-0 pA: LIII vs. LX	0.9503	(*t*) 0.062 (*df*) 748	0.11 ± 0.1 mV vs. 0.01 ± 0.0 mV	9 vs. 15 cells
		-0 pA: LIII vs. LX	0.8939	(*t*) 0.133 (*df*) 748	0.18 ± 0.2 mV vs. -0.04 ± 0.0 mV	9 vs. 15 cells
		-0 pA: LIII vs. LX	0.9374	(*t*) 0.079 (*df*) 748	0.13 ± 0.2 mV vs. -0.01 ± 0.1 mV	9 vs. 15 cells
		-0 pA: LIII vs. LX	0.9191	(*t*) 0.102 (*df*) 748	0.14 ± 0.2 mV vs. -0.03 ± 0.0 mV	9 vs. 15 cells
		-0 pA: LIII vs. LX	0.9394	(*t*) 0.076 (*df*) 748	0.09 ± 0.2 mV vs. -0.04 ± 0.1 mV	9 vs. 15 cells
		-0 pA: LIII vs. LX	0.8930	(*t*) 0.135 (*df*) 748	0.16 ± 0.2 mV vs. -0.07 ± 0.1 mV	9 vs. 15 cells
		-40 pA: LIII vs. LX	0.9745	(*t*) 0.032 (*df*) 748	-0.50 ± 0.2 mV vs. -0.55 ± 0.1 mV	9 vs. 15 cells
		-80 pA: LIII vs. LX	**0.0292**	(*t*) 2.185 (*df*) 748	-7.40 ± 2.1 mV vs. -3.73 ± 1.3 mV	9 vs. 15 cells
		-120 pA: LIII vs. LX	**0.0013**	(*t*) 3.226 (*df*) 748	-15.26 ± 2.5 mV vs. -9.85 ± 2.1 mV	9 vs. 15 cells
		-160 pA: LIII vs. LX	0.6269	(*t*) 0.486 (*df*) 748	-18.03 ± 1.5 mV vs. -17.21 ± 1.7 mV	9 vs. 15 cells
		-200 pA: LIII vs. LX	0.1484	(*t*) 1.447 (*df*) 748	-19.62 ± 1.4 mV vs. -22.05 ± 1.8 mV	9 vs. 15 cells
		-240 pA: LIII vs. LX	0.1093	(*t*) 1.603 (*df*) 748	-21.44 ± 1.5 mV vs. -24.13 ± 1.8 mV	9 vs. 15 cells
Figure 6A1	Two-way ANOVA	-280 pA: LIII vs. LX	**0.0106**	(*t*) 2.563 (*df*) 748	-23.24 ± 1.6 mV vs. -27.55 ± 1.4 mV	9 vs. 15 cells
		-320 pA: LIII vs. LX	**0.0012**	(*t*) 3.248 (*df*) 748	-24.58 ± 1.7 mV vs. -30.03 ± 1.7 mV	9 vs. 15 cells
		-280 pA: LIII vs. LX	**0.0091**	(*t*) 2.617 (*df*) 748	-21.83 ± 1.6 mV vs. -26.22 ± 1.6 mV	9 vs. 15 cells
		-240 pA: LIII vs. LX	**0.0327**	(*t*) 2.140 (*df*) 748	-20.11 ± 1.4 mV vs. -23.70 ± 1.4 mV	9 vs. 15 cells
		-200 pA: LIII vs. LX	0.0795	(*t*) 1.756 (*df*) 748	-18.07 ± 1.3 mV vs. -21.02 ± 1.4 mV	9 vs. 15 cells
		-160 pA: LIII vs. LX	0.1504	(*t*) 1.440 (*df*) 748	-15.9. ± 1.3 mV vs. -18.35 ± 1.4 mV	9 vs. 15 cells
		-120 pA: LIII vs. LX	0.2127	(*t*) 1.247 (*df*) 748	-13.56 ± 1.2 mV vs. -15.66 ± 1.5 mV	9 vs. 15 cells
		-80 pA: LIII vs. LX	0.4610	(*t*) 0.738 (*df*) 748	-11.17 ± 1.1 mV vs. -12.41 ± 1.6 mV	9 vs. 15 cells
		-40 pA: LIII vs. LX	0.2649	(*t*) 1.116 (*df*) 748	-7.05 ± 1.6 mV vs. -8.92 ± 1.7 mV	9 vs. 15 cells
		-0 pA: LIII vs. LX	0.2222	(*t*) 1.222 (*df*) 748	-2.79 ± 0.9 mV vs. -4.84 ± 1.3 mV	9 vs. 15 cells
		-0 pA: LIII vs. LX	0.3454	(*t*) 0.944 (*df*) 748	-1.84 ± 0.6 mV vs. -3.43 ± 1.3 mV	9 vs. 15 cells
		-0 pA: LIII vs. LX	0.5009	(*t*) 0.673 (*df*) 748	-1.51 ± 0.6 mV vs. -2.64 ± 1.1 mV	9 vs. 15 cells
		-0 pA: LIII vs. LX	0.6346	(*t*) 0.476 (*df*) 748	-0.98 ± 0.5 mV vs. -1.78 ± 1.1 mV	9 vs. 15 cells
		-0 pA: LIII vs. LX	0.6565	(*t*) 0.445 (*df*) 748	-0.85 ± 0.4 mV vs. -1.59 ± 0.9 mV	9 vs. 15 cells
		-0 pA: LIII vs. LX	0.7153	(*t*) 0.365 (*df*) 748	-0.73 ± 0.4 mV vs. -1.34 ± 0.7 mV	9 vs. 15 cells
		-0 pA: LIII vs. LX	0.8853	(*t*) 0.144 (*df*) 748	-0.62 ± 0.4 mV vs. -0.86 ± 0.4 mV	9 vs. 15 cells
		-0 pA: LIII vs. LX	0.8547	(*t*) 0.183 (*df*) 748	-0.31 ± 0.2 mV vs. -0.61 ± 0.3 mV	9 vs. 15 cells
		-0 pA: LIII vs. LX	0.9434	(*t*) 0.071 (*df*) 748	-0.34 ± 0.2 mV vs. -0.22 ± 0.1mV	9 vs. 15 cells
		-0 pA: LIII↓ vs. LIII↑	0.7208	(*t*) 0.358 (*df*) 128	0.01 ± 0.1 mV vs. -0.34 ± 0.2 mV	
		-0 pA: LIII↓ vs. LIII↑	0.6707	(*t*) 0.426 (*df*) 128	0.11 ± 0.1 mV vs. -0.31 ± 0.2 mV	
		-0 pA: LIII↓ vs. LIII↑	0.5143	(*t*) 0.654 (*df*) 128	0.02 ± 0.1 mV vs. -0.62 ± 0.4 mV	
		-0 pA: LIII↓ vs. LIII↑	0.3925	(*t*) 0.858 (*df*) 128	0.11 ± 0.1 mV vs. -0.73 ± 0.4 mV	
		-0 pA: LIII↓ vs. LIII↑	0.2969	(*t*) 1.047 (*df*) 128	0.18 ± 0.2 mV vs. -0.85 ± 0.4 mV	
		-0 pA: LIII↓ vs. LIII↑	0.2610	(*t*) 1.129 (*df*) 128	0.13 ± 0.2 mV vs. -0.98 ± 0.5 mV	
		-0 pA: LIII↓ vs. LIII↑	0.0963	(*t*) 1.675 (*df*) 128	0.14 ± 0.2 mV vs. -1.51 ± 0.6 mV	
		-0 pA: LIII↓ vs. LIII↑	0.0510	(*t*) 1.970 (*df*) 128	0.09 ± 0.2 mV vs. -1.84 ± 0.6 mV	
Figure 6A2	Two-way ANOVA	-0 pA: LIII↓ vs. LIII↑	**0.0032**	(*t*) 3.003 (*df*) 128	0.16 ± 0.2 mV vs. -2.79 ± 0.9 mV	9 cells
		-40 pA: LIII↓ vs. LIII↑	**<0.0001**	(*t*) 6.684 (*df*) 128	-0.50 ± 0.2 mV vs. -7.05 ± 1.6 mV	
		-80 pA: LIII↓ vs. LIII↑	**0.0002**	(*t*) 3.852 (*df*) 128	-7.40 ± 2.1 mV vs. -11.17 ± 1.1 mV	
		-120 pA: LIII↓ vs. LIII↑	0.0853	(*t*) 1.734 (*df*) 128	-15.26 ± 2.5 mV vs. -13.56 ± 1.2 mV	
		-160 pA: LIII↓ vs. LIII↑	**0.0342**	(*t*) 2.141 (*df*) 128	-18.03 ± 1.5 mV vs. -15.93 ± 1.3 mV	
		-200 pA: LIII↓ vs. LIII↑	0.1171	(*t*) 1.578 (*df*) 128	-19.62 ± 1.4 mV vs. -18.07 ± 1.3 mV	
		-240 pA: LIII↓ vs. LIII↑	0.1772	(*t*) 1.357 (*df*) 128	-21.44 ± 1.5 mV vs. -20.11 ± 1.4 mV	
		-280 pA: LIII↓ vs. LIII↑	0.1512	(*t*) 1.444 (*df*) 128	-23.24 ± 1.6 mV vs. -21.83 ± 1.6 mV	
		-320 pA			-24.58 ± 1.7 mV	
		-0 pA: LX↓ vs. LX↑	0.8897	(*t*) 0.139 (*df*) 224	-0.07 ± 0.0 mV vs. -0.22 ± 0.1 mV	
		-0 pA: LX↓ vs. LX↑	0.5891	(*t*) 0.541 (*df*) 224	-0.02 ± 0.0 mV vs. -0.61 ± 0.3 mV	
Figure 6A3	Two-way ANOVA	-0 pA: LX↓ vs. LX↑	0.4272	(*t*) 0.800 (*df*) 224	0.00 ± 0.0 mV vs. -0.86 ± 0.4 mV	15 cells
		-0 pA: LX↓ vs. LX↑	0.2168	(*t*) 1.239 (*df*) 224	0.01 ± 0.0 mV vs. -1.34 ± 0.7 mV	
		-0 pA: LX↓ vs. LX↑	0.1562	(*t*) 1.423 (*df*) 224	-0.04 ± 0.0 mV vs. -1.59 ± 0.9 mV	
		-0 pA: LX↓ vs. LX↑	0.1050	(*t*) 1.628 (*df*) 224	-0.01 ± 0.1 mV vs. -1.78 ± 1.1 mV	

		-0 pA: LX↓ vs. LX↑	**0.0177**	(*t*) 2.389 (*df*) 224	-0.03 ± 0.0 mV vs. -2.64 ± 1.1 mV	
		-0 pA: LX↓ vs. LX↑	**0.0021**	(*t*) 3.111 (*df*) 224	-0.04 ± 0.1 mV vs. -3.43 ± 1.3 mV	
		-0 pA: LX↓ vs. LX↑	**<0.0001**	(*t*) 4.378 (*df*) 224	-0.07 ± 0.1 mV vs. -4.84 ± 1.3 mV	
		-40 pA: LX↓ vs. LX↑	**<0.0001**	(*t*) 7.685 (*df*) 224	-0.55 ± 0.1 mV vs. -8.92 ± 1.7 mV	
		-80 pA: LX↓ vs. LX↑	**<0.0001**	(*t*) 7.972 (*df*) 224	-3.73 ± 1.3 mV vs. -12.41 ± 1.6 mV	
		-120 pA: LX↓ vs. LX↑	**<0.0001**	(*t*) 5.336 (*df*) 224	-9.85 ± 2.1 mV vs. -15.66 ± 1.5 mV	
		-160 pA: LX↓ vs. LX↑	0.2982	(*t*) 1.043 (*df*) 224	-17.21 ± 1.7 mV vs. -18.35 ± 1.4 mV	
		-200 pA: LX↓ vs. LX↑	0.3470	(*t*) 0.942 (*df*) 224	-22.05 ± 1.8 mV vs. -21.02 ± 1.4 mV	
		-240 pA: LX↓ vs. LX↑	0.6944	(*t*) 0.394 (*df*) 224	-24.13 ± 1.8 mV vs. -23.70 ± 1.4 mV	
		-280 pA: LX↓ vs. LX↑	0.2251	(*t*) 1.216 (*df*) 224	-27.55 ± 1.4 mV vs. -26.22 ± 1.6 mV	
		-320 pA			-30.03 ± 1.7 mV	
Figure 6B1	Two-way ANOVA	-0 pA: LIII vs. LX	0.4425	(*t*) 0.773 (*df*) 68	42.06 ± 9.0 Hz vs. 42.94 ± 7.0 Hz	9 vs. 15 cells
		-0 pA: LIII vs. LX	0.5014	(*t*) 0.676 (*df*) 68	42.49 ± 9.2 Hz vs. 43.12 ± 7.0 Hz	9 vs. 15 cells
		-0 pA: LIII vs. LX	0.4813	(*t*) 0.708 (*df*) 68	43.73 ± 9.1 Hz vs. 43.71 ± 7.2 Hz	9 vs. 15 cells
		-0 pA: LIII vs. LX	0.5014	(*t*) 0.676 (*df*) 68	44.43 ± 9.5 Hz vs. 43.95 ± 7.1 Hz	9 vs. 15 cells
		-0 pA: LIII vs. LX	0.4425	(*t*) 0.773 (*df*) 68	45.45 ± 9.5 Hz vs. 44.39 ± 7.2 Hz	9 vs. 15 cells
		-0 pA: LIII vs. LX	0.4813	(*t*) 0.708 (*df*) 68	45.62 ± 9.4 Hz vs. 44.16 ± 7.2 Hz	9 vs. 15 cells
		-0 pA: LIII vs. LX	0.4425	(*t*) 0.773 (*df*) 68	45.85 ± 9.4 Hz vs. 44.37 ± 7.1 Hz	9 vs. 15 cells
		-0 pA: LIII vs. LX	0.3377	(*t*) 0.708 (*df*) 68	46.17 ± 9.8 Hz vs. 44.48 ± 7.2 Hz	9 vs. 15 cells
		-0 pA: LIII vs. LX	0.4813	(*t*) 0.773 (*df*) 68	46.80 ± 9.6 Hz vs. 44.39 ± 7.1 Hz	9 vs. 15 cells
		-0 pA: LIII vs. LX	0.5014	(*t*) 0.966 (*df*) 68	47.29 ± 10.0 Hz vs. 44.49 ± 7.1 Hz	9 vs. 15 cells
		-40 pA: LIII vs. LX	0.5642	(*t*) 0.708 (*df*) 68	34.41 ± 8.6 Hz vs. 37.28 ± 7.0 Hz	9 vs. 15 cells
		-80 pA: LIII vs. LX	0.8474	(*t*) 0.676 (*df*) 68	10.51 ± 5.3 Hz vs. 24.99 ± 6.7 Hz	9 vs. 15 cells
		-120 pA: LIII vs. LX	0.6769	(*t*) 0.579 (*df*) 68	2.00 ± 1.7 Hz vs. 13.07 ± 5.7 Hz	9 vs. 15 cells
		-160 pA: LIII vs. LX	0.9744	(*t*) 0.193 (*df*) 68	0.00 ± 0.0 Hz vs. 6.59 ± 4.5 Hz	9 vs. 15 cells
		-200 pA: LIII vs. LX	>0.9999	(*t*) 0.418 (*df*) 68	0.00 ± 0.0 Hz vs. 3.43 ± 3.3 Hz	9 vs. 15 cells
		-240 pA: LIII vs. LX	>0.9999	(*t*) 0.032 (*df*) 68	0.00 ± 0.0 Hz vs. 2.07 ± 2.1 Hz	9 vs. 15 cells
		-280 pA: LIII vs. LX	>0.9999	(*t*) 0.000 (*df*) 68	0.00 ± 0.0 Hz vs. 0.15 ± 0.1 Hz	9 vs. 15 cells
		-320 pA: LIII vs. LX	>0.9999	(*t*) 0.000 (*df*) 68	0.00 ± 0.0 Hz vs. 0.00 ± 0.0 Hz	9 vs. 15 cells
		-280 pA: LIII vs. LX	>0.9999	(*t*) 0.000 (*df*) 68	0.00 ± 0.0 Hz vs. 0.00 ± 0.0 Hz	9 vs. 15 cells
		-240 pA: LIII vs. LX	>0.9999	(*t*) 0.000 (*df*) 68	0.00 ± 0.0 Hz vs. 0.00 ± 0.0 Hz	9 vs. 15 cells
		-200 pA: LIII vs. LX	>0.9999	(*t*) 0.000 (*df*) 68	0.00 ± 0.0 Hz vs. 0.00 ± 0.0 Hz	9 vs. 15 cells
		-160 pA: LIII vs. LX	>0.9999	(*t*) 0.000 (*df*) 68	0.00 ± 0.0 Hz vs. 0.00 ± 0.0 Hz	9 vs. 15 cells
		-120 pA: LIII vs. LX	>0.9999	(*t*) 0.000 (*df*) 68	0.00 ± 0.0 Hz vs. 0.00 ± 0.0 Hz	9 vs. 15 cells
		-80 pA: LIII vs. LX	>0.9999	(*t*) 0.000 (*df*) 68	0.90 ± 0.8 Hz vs. 1.03 ± 0.9 Hz	9 vs. 15 cells
		-40 pA: LIII vs. LX	0.9744	(*t*) 0.032 (*df*) 68	13.66 ± 8.1 Hz vs. 4.03 ± 2.2 Hz	9 vs. 15 cells
		-0 pA: LIII vs. LX	0.9234	(*t*) 0.097 (*df*) 68	21.86 ± 9.8 Hz vs. 9.69 ± 2.8 Hz	9 vs. 15 cells
		-0 pA: LIII vs. LX	0.8726	(*t*) 0.161 (*df*) 68	25.37 ± 9.1 Hz vs. 17.63 ± 4.6 Hz	9 vs. 15 cells
		-0 pA: LIII vs. LX	0.7485	(*t*) 0.322 (*df*) 68	27.74 ± 8.9 Hz vs. 22.61 ± 4.8 Hz	9 vs. 15 cells
		-0 pA: LIII vs. LX	0.7729	(*t*) 0.290 (*df*) 68	29.40 ± 8.2 Hz vs. 26.58 ± 5.1 Hz	9 vs. 15 cells
		-0 pA: LIII vs. LX	0.5642	(*t*) 0.579 (*df*) 68	31.97 ± 8.1 Hz vs. 29.81 ± 5.1 Hz	9 vs. 15 cells
		-0 pA: LIII vs. LX	0.5429	(*t*) 0.612 (*df*) 68	33.80 ± 8.3 Hz vs. 31.58 ± 5.2 Hz	9 vs. 15 cells
		-0 pA: LIII vs. LX	0.5429	(*t*) 0.612 (*df*) 68	34.96 ± 8.4 Hz vs. 34.45 ± 5.5 Hz	9 vs. 15 cells
		-0 pA: LIII vs. LX	0.5642	(*t*) 0.579 (*df*) 68	36.08 ± 8.0 Hz vs. 36.33 ± 5.7 Hz	9 vs. 15 cells
		-0 pA: LIII vs. LX	0.3706	(*t*) 0.901 (*df*) 68	38.46 ± 8.6 Hz vs. 38.73 ± 6.1 Hz	9 vs. 15 cells
Figure 6B2	Two-way ANOVA	-0 pA: LIII↓ vs. LIII↑	0.2434	(*t*) 1.172 (*df*) 128	42.49 ± 9.2 Hz vs. 38.46 ± 8.6 Hz	9 cells
		-0 pA: LIII↓ vs. LIII↑	**0.0278**	(*t*) 2.226 (*df*) 128	43.73 ± 9.1 Hz vs. 36.08 ± 8.0 Hz	
		-0 pA: LIII↓ vs. LIII↑	**0.0067**	(*t*) 2.754 (*df*) 128	44.43 ± 9.5 Hz vs. 34.96 ± 8.4 Hz	
		-0 pA: LIII↓ vs. LIII↑	**0.0009**	(*t*) 3.389 (*df*) 128	45.45 ± 9.5 Hz vs. 33.80 ± 8.3 Hz	
		-0 pA: LIII↓ vs. LIII↑	**0.0001**	(*t*) 3.970 (*df*) 128	45.62 ± 9.4 Hz vs. 31.97 ± 8.1 Hz	
		-0 pA: LIII↓ vs. LIII↑	**<0.0001**	(*t*) 4.781 (*df*) 128	45.85 ± 9.4 Hz vs. 29.40 ± 8.2 Hz	
		-0 pA: LIII↓ vs. LIII↑	**<0.0001**	(*t*) 5.360 (*df*) 128	46.17 ± 9.8 Hz vs. 27.74 ± 8.9 Hz	
		-0 pA: LIII↓ vs. LIII↑	**<0.0001**	(*t*) 6.231 (*df*) 128	46.80 ± 9.6 Hz vs. 25.37 ± 9.1 Hz	
		-0 pA: LIII↓ vs. LIII↑	**<0.0001**	(*t*) 7.393 (*df*) 128	47.29 ± 10.0 Hz vs. 21.86 ± 9.8 Hz	
		-40 pA: LIII↓ vs. LIII↑	**<0.0001**	(*t*) 6.033 (*df*) 128	34.41 ± 8.6 Hz vs. 13.66 ± 8.1 Hz	
		-80 pA: LIII↓ vs. LIII↑	**0.0060**	(*t*) 2.792 (*df*) 128	10.51 ± 5.3 Hz vs. 0.90 ± 0.8 Hz	
		-120 pA: LIII↓ vs. LIII↑	0.5619	(*t*) 0.582 (*df*) 128	2.00 ± 1.7 Hz vs. 0.00 ± 0.0 Hz	
		-160 pA: LIII↓ vs. LIII↑	>0.9999	(*t*) 0.000 (*df*) 128	0.00 ± 0.0 Hz vs. 0.00 ± 0.0 Hz	
		-200 pA: LIII↓ vs. LIII↑	>0.9999	(*t*) 0.000 (*df*) 128	0.00 ± 0.0 Hz vs. 0.00 ± 0.0 Hz	
		-240 pA: LIII↓ vs. LIII↑	>0.9999	(*t*) 0.000 (*df*) 128	0.00 ± 0.0 Hz vs. 0.00 ± 0.0 Hz	
		-280 pA: LIII↓ vs. LIII↑	>0.9999	(*t*) 0.000 (*df*) 128	0.00 ± 0.0 Hz vs. 0.00 ± 0.0 Hz	
		-320 pA			0.00 ± 0.0 Hz	
Figure 6B3	Two-way ANOVA	-0 pA: LX↓ vs. LX↑	0.1967	(*t*) 1.295 (*df*) 224	43.12 ± 7.0 Hz vs. 38.73 ± 6.1 Hz	15 cells
		-0 pA: LX↓ vs. LX↑	**0.0303**	(*t*) 2.179 (*df*) 224	43.71 ± 7.2 Hz vs. 36.33 ± 5.7 Hz	

		-0 pA: LX↓ vs. LX↑	**0.0055**	(*t*) 2.804 (*df*) 224	43.95 ± 7.1 Hz vs. 34.45 ± 5.5 Hz	
		-0 pA: LX↓ vs. LX↑	**0.0002**	(*t*) 3.783 (*df*) 224	44.39 ± 7.2 Hz vs. 31.58 ± 5.2 Hz	
		-0 pA: LX↓ vs. LX↑	**<0.0001**	(*t*) 5.190 (*df*) 224	44.16 ± 7.2 Hz vs. 26.58 ± 5.1 Hz	
		-0 pA: LX↓ vs. LX↑	**<0.0001**	(*t*) 6.423 (*df*) 224	44.37 ± 7.1 Hz vs. 22.61 ± 4.8 Hz	
		-0 pA: LX↓ vs. LX↑	**<0.0001**	(*t*) 7.925 (*df*) 224	44.48 ± 7.2 Hz vs. 17.63 ± 4.6 Hz	
		-0 pA: LX↓ vs. LX↑	**<0.0001**	(*t*) 10.24 (*df*) 224	44.39 ± 7.1 Hz vs. 9.69 ± 2.8 Hz	
		-0 pA: LX↓ vs. LX↑	**<0.0001**	(*t*) 11.94 (*df*) 224	44.49 ± 7.1 Hz vs. 4.03 ± 2.2 Hz	
		-40 pA: LX↓ vs. LX↑	**<0.0001**	(*t*) 10.70 (*df*) 224	37.28 ± 7.0 Hz vs. 1.03 ± 0.9 Hz	
		-80 pA: LX↓ vs. LX↑	**<0.0001**	(*t*) 7.379 (*df*) 224	24.99 ± 6.7 Hz vs. 0.00 ± 0.0 Hz	
		-120 pA: LX↓ vs. LX↑	**0.0001**	(*t*) 3.858 (*df*) 224	13.07 ± 5.7 Hz vs. 0.00 ± 0.0 Hz	
		-160 pA: LX↓ vs. LX↑	0.0530	(*t*) 1.946 (*df*) 224	6.59 ± 4.5 Hz vs. 0.00 ± 0.0 Hz	
		-200 pA: LX↓ vs. LX↑	0.3129	(*t*) 1.011 (*df*) 224	3.43 ± 3.3 Hz vs. 0.00 ± 0.0 Hz	
		-240 pA: LX↓ vs. LX↑	0.5409	(*t*) 0.612 (*df*) 224	2.07 ± 2.1 Hz vs. 0.00 ± 0.0 Hz	
		-280 pA: LX↓ vs. LX↑	0.9652	(*t*) 0.044 (*df*) 224	0.15 ± 0.1 Hz vs. 0.00 ± 0.0 Hz	
		-320 pA			0.00 ± 0.0 Hz	
		0 pA: LIII vs. LX			0.00 ± 0.0 mV vs. 0.00 ± 0.0 mV	8 vs. 14 cells
		0 pA: LIII vs. LX	0.9931	(*t*) 0.009 (*df*) 68	-0.03 ± 0.0 mV vs. 0.00 ± 0.0 mV	8 vs. 14 cells
		0 pA: LIII vs. LX	0.9942	(*t*) 0.007 (*df*) 68	0.03 ± 0.0 mV vs. 0.01 ± 0.0 mV	8 vs. 14 cells
		0 pA: LIII vs. LX	0.9919	(*t*) 0.010 (*df*) 68	0.07 ± 0.0 mV vs. 0.03 ± 0.0 mV	8 vs. 14 cells
		0 pA: LIII vs. LX	0.9977	(*t*) 0.003 (*df*) 68	0.09 ± 0.0 mV vs. 0.03 ± 0.0 mV	8 vs. 14 cells
		0 pA: LIII vs. LX	0.9816	(*t*) 0.023 (*df*) 68	0.11 ± 0.0 mV vs. 0.05 ± 0.0 mV	8 vs. 14 cells
		0 pA: LIII vs. LX	0.9885	(*t*) 0.014 (*df*) 68	0.07 ± 0.0 mV vs. 0.08 ± 0.0 mV	8 vs. 14 cells
		0 pA: LIII vs. LX	0.9850	(*t*) 0.019 (*df*) 68	0.14 ± 0.0 mV vs. 0.03 ± 0.0 mV	8 vs. 14 cells
		0 pA: LIII vs. LX	0.9850	(*t*) 0.019 (*df*) 68	0.16 ± 0.0 mV vs. 0.07 ± 0.0 mV	8 vs. 14 cells
		0 pA: LIII vs. LX	0.9954	(*t*) 0.006 (*df*) 68	0.15 ± 0.0 mV vs. 0.10 ± 0.0 mV	8 vs. 14 cells
		40 pA: LIII vs. LX	0.9425	(*t*) 0.072 (*df*) 68	0.76 ± 0.1 mV vs. 0.30 ± 0.1 mV	8 vs. 14 cells
		80 pA: LIII vs. LX	0.7488	(*t*) 0.322 (*df*) 68	4.77 ± 0.5 mV vs. 5.95 ± 0.8 mV	8 vs. 14 cells
		120 pA: LIII vs. LX	0.9977	(*t*) 0.003 (*df*) 68	9.52 ± 1.9 mV vs. 10.19 ± 0.9 mV	8 vs. 14 cells
		160 pA: LIII vs. LX	0.3919	(*t*) 0.862 (*df*) 68	13.27 ± 1.8 mV vs. 14.14 ± 0.9 mV	8 vs. 14 cells
		200 pA: LIII vs. LX	0.7002	(*t*) 0.387 (*df*) 68	16.07 ± 1.5 mV vs. 17.33 ± 1.0 mV	8 vs. 14 cells
		240 pA: LIII vs. LX	0.5403	(*t*) 0.615 (*df*) 68	17.89 ± 1.3 mV vs. 18.42 ± 1.0 mV	8 vs. 14 cells
Figure 6C1	Two-way ANOVA	280 pA: LIII vs. LX	0.4689	(*t*) 0.728 (*df*) 68	19.04 ± 1.4 mV vs. 19.72 ± 1.0 mV	8 vs. 14 cells
		320 pA: LIII vs. LX	0.5375	(*t*) 0.620 (*df*) 68	19.91 ± 1.5 mV vs. 20.34 ± 1.1 mV	8 vs. 14 cells
		280 pA: LIII vs. LX	0.4760	(*t*) 0.719 (*df*) 68	19.20 ± 1.4 mV vs. 19.91 ± 1.1 mV	8 vs. 14 cells
		240 pA: LIII vs. LX	0.5318	(*t*) 0.629 (*df*) 68	18.17 ± 1.4 mV vs. 18.95 ± 1.1 mV	8 vs. 14 cells
		200 pA: LIII vs. LX	0.6099	(*t*) 0.513 (*df*) 68	17.07 ± 1.4 mV vs. 18.08 ± 1.1 mV	8 vs. 14 cells
		160 pA: LIII vs. LX	0.8694	(*t*) 0.165 (*df*) 68	13.59 ± 2.3 mV vs. 16.85 ± 1.0 mV	8 vs. 14 cells
		120 pA: LIII vs. LX	0.9104	(*t*) 0.113 (*df*) 68	10.68 ± 2.4 mV vs. 14.86 ± 1.4 mV	8 vs. 14 cells
		80 pA: LIII vs. LX	0.4813	(*t*) 0.708 (*df*) 68	5.94 ± 2.8 mV vs. 12.04 ± 1.8 mV	8 vs. 14 cells
		40 pA: LIII vs. LX	**0.0452**	(*t*) 2.040 (*df*) 68	2.27 ± 2.4 mV vs. 9.04 ± 2.1 mV	8 vs. 14 cells
		0 pA: LIII vs. LX	**0.0319**	(*t*) 2.191 (*df*) 68	-1.71 ± 1.0 mV vs. -1.52 ± 0.9 mV	8 vs. 14 cells
		0 pA: LIII vs. LX	0.7379	(*t*) 0.336 (*df*) 68	-0.41 ± 0.1 mV vs. -0.78 ± 0.4 mV	8 vs. 14 cells
		0 pA: LIII vs. LX	0.7863	(*t*) 0.272 (*df*) 68	-0.35 ± 0.1 mV vs. -0.56 ± 0.1 mV	8 vs. 14 cells
		0 pA: LIII vs. LX	0.9908	(*t*) 0.116 (*df*) 68	-0.33 ± 0.1 mV vs. -0.45 ± 0.1 mV	8 vs. 14 cells
		0 pA: LIII vs. LX	0.9977	(*t*) 0.003 (*df*) 68	-0.24 ± 0.1 mV vs. -0.35 ± 0.1 mV	8 vs. 14 cells
		0 pA: LIII vs. LX	0.9965	(*t*) 0.004 (*df*) 68	-0.22 ± 0.1 mV vs. -0.27 ± 0.1 mV	8 vs. 14 cells
		0 pA: LIII vs. LX	0.9896	(*t*) 0.013 (*df*) 68	-0.15 ± 0.1 mV vs. -0.26 ± 0.1 mV	8 vs. 14 cells
		0 pA: LIII vs. LX	0.9862	(*t*) 0.017 (*df*) 68	-0.13 ± 0.0 mV vs. -0.25 ± 0.1 mV	8 vs. 14 cells
		0 pA: LIII vs. LX	0.9655	(*t*) 0.043 (*df*) 68	-0.10 ± 0.0 mV vs. -0.22 ± 0.1 mV	8 vs. 14 cells
		0 pA: LIII↓ vs. LIII↑	0.9454	(*t*) 0.069 (*df*) 112	-0.03 ± 0.0 mV vs. -0.10 ± 0.0 mV	
		0 pA: LIII↓ vs. LIII↑	0.8752	(*t*) 0.157 (*df*) 112	0.03 ± 0.0 mV vs. -0.13 ± 0.0 mV	
		0 pA: LIII↓ vs. LIII↑	0.8284	(*t*) 0.217 (*df*) 112	0.07 ± 0.0 mV vs. -0.15 ± 0.1 mV	
		0 pA: LIII↓ vs. LIII↑	0.7564	(*t*) 0.311 (*df*) 112	0.09 ± 0.0 mV vs. -0.22 ± 0.1 mV	
		0 pA: LIII↓ vs. LIII↑	0.7272	(*t*) 0.350 (*df*) 112	0.11 ± 0.0 mV vs. -0.24 ± 0.1 mV	
		0 pA: LIII↓ vs. LIII↑	0.6978	(*t*) 0.393 (*df*) 112	0.07 ± 0.0 mV vs. -0.33 ± 0.1 mV	
		0 pA: LIII↓ vs. LIII↑	0.6254	(*t*) 0.490 (*df*) 112	0.14 ± 0.0 mV vs. -0.35 ± 0.1 mV	
Figure 6C2	Two-way ANOVA	0 pA: LIII↓ vs. LIII↑	0.5676	(*t*) 0.573 (*df*) 112	0.16 ± 0.0 mV vs. -0.41 ± 0.1 mV	8 cells
		0 pA: LIII↓ vs. LIII↑	0.0657	(*t*) 1.858 (*df*) 112	0.15 ± 0.0 mV vs. -1.71 ± 1.0 mV	
		40 pA: LIII↓ vs. LIII↑	0.1329	(*t*) 1.514 (*df*) 112	0.76 ± 0.1 mV vs. 2.27 ± 2.4 mV	
		80 pA: LIII↓ vs. LIII↑	0.2444	(*t*) 1.170 (*df*) 112	4.77 ± 0.5 mV vs. 5.94 ± 2.8 mV	
		120 pA: LIII↓ vs. LIII↑	0.2499	(*t*) 1.157 (*df*) 112	9.52 ± 1.9 mV vs. 10.68 ± 2.4 mV	
		160 pA: LIII↓ vs. LIII↑	0.7517	(*t*) 0.317 (*df*) 112	13.27 ± 1.8 mV vs. 13.59 ± 2.3 mV	
		200 pA: LIII↓ vs. LIII↑	0.3205	(*t*) 0.998 (*df*) 112	16.07 ± 1.5 mV vs. 17.07 ± 1.4 mV	
		240 pA: LIII↓ vs. LIII↑	0.7754	(*t*) 0.286 (*df*) 112	17.89 ± 1.3 mV vs. 18.17 ± 1.4 mV	

		-280 pA: LIII↓ vs. LIII↑	0.5762	(*t*) 0.156 (*df*) 112	19.04 ± 1.4 mV vs. 19.20 ± 1.4 mV	
		-320 pA			19.81 ± 1.5 mV	
Figure 6C3	Two-way ANOVA	0 pA: LX↓ vs. LX↑	0.8004	(*t*) 0.253 (*df*) 208	0.00 ± 0.0 mV vs. -0.22 ± 0.1 mV	14 cells
		0 pA: LX↓ vs. LX↑	0.7591	(*t*) 0.307 (*df*) 208	0.01 ± 0.0 mV vs. -0.25 ± 0.1 mV	
		0 pA: LX↓ vs. LX↑	0.7415	(*t*) 0.330 (*df*) 208	0.03 ± 0.0 mV vs. -0.26 ± 0.1 mV	
		0 pA: LX↓ vs. LX↑	0.7334	(*t*) 0.341 (*df*) 208	0.03 ± 0.0 mV vs. -0.27 ± 0.1 mV	
		0 pA: LX↓ vs. LX↑	0.6449	(*t*) 0.462 (*df*) 208	0.05 ± 0.0 mV vs. -0.35 ± 0.1 mV	
		0 pA: LX↓ vs. LX↑	0.8387	(*t*) 0.616 (*df*) 208	0.08 ± 0.0 mV vs. -0.45 ± 0.1 mV	
		0 pA: LX↓ vs. LX↑	0.4937	(*t*) 0.686 (*df*) 208	0.03 ± 0.0 mV vs. -0.56 ± 0.1 mV	
		0 pA: LX↓ vs. LX↑	0.3240	(*t*) 0.989 (*df*) 208	0.07 ± 0.0 mV vs. -0.78 ± 0.4 mV	
		0 pA: LX↓ vs. LX↑	0.0613	(*t*) 1.882 (*df*) 208	0.10 ± 0.0 mV vs. -1.52 ± 0.9 mV	
		40 pA: LX↓ vs. LX↑	**<0.0001**	(*t*) 9.802 (*df*) 208	0.60 ± 0.1 mV vs. 9.04 ± 2.1 mV	
		80 pA: LX↓ vs. LX↑	**<0.0001**	(*t*) 7.077 (*df*) 208	5.95 ± 0.8 mV vs. 12.04 ± 1.8 mV	
		120 pA: LX↓ vs. LX↑	**<0.0001**	(*t*) 5.431 (*df*) 208	10.19 ± 0.9 mV vs. 14.86 ± 1.4 mV	
		160 pA: LX↓ vs. LX↑	**0.0049**	(*t*) 3.145 (*df*) 208	14.14 ± 0.9 mV vs. 16.85 ± 1.0 mV	
		200 pA: LX↓ vs. LX↑	0.3886	(*t*) 0.864 (*df*) 208	17.33 ± 1.0 mV vs. 18.08 ± 1.1 mV	
		240 pA: LX↓ vs. LX↑	0.5354	(*t*) 0.621 (*df*) 208	18.42 ± 1.0 mV vs. 18.95 ± 1.1 mV	
		280 pA: LX↓ vs. LX↑	0.8203	(*t*) 0.227 (*df*) 208	19.72 ± 1.0 mV vs. 19.91 ± 1.1 mV	
		320 pA			20.34 ± 1.1 mV	
Figure 6D1	Two-way ANOVA	0 pA: LIII vs. LX	>0.9999	(*t*) 0.000 (*df*) 680	0.00 ± 0.0 Hz vs. 0.00 ± 0.0 Hz	8 vs. 14 cells
		0 pA: LIII vs. LX	>0.9999	(*t*) 0.000 (*df*) 680	0.00 ± 0.0 Hz vs. 0.00 ± 0.0 Hz	8 vs. 14 cells
		0 pA: LIII vs. LX	>0.9999	(*t*) 0.000 (*df*) 680	0.00 ± 0.0 Hz vs. 0.00 ± 0.0 Hz	8 vs. 14 cells
		0 pA: LIII vs. LX	>0.9999	(*t*) 0.000 (*df*) 680	0.00 ± 0.0 Hz vs. 0.00 ± 0.0 Hz	8 vs. 14 cells
		0 pA: LIII vs. LX	>0.9999	(*t*) 0.000 (*df*) 680	0.00 ± 0.0 Hz vs. 0.00 ± 0.0 Hz	8 vs. 14 cells
		0 pA: LIII vs. LX	>0.9999	(*t*) 0.000 (*df*) 680	0.00 ± 0.0 Hz vs. 0.00 ± 0.0 Hz	8 vs. 14 cells
		0 pA: LIII vs. LX	>0.9999	(*t*) 0.000 (*df*) 680	0.00 ± 0.0 Hz vs. 0.00 ± 0.0 Hz	8 vs. 14 cells
		0 pA: LIII vs. LX	>0.9999	(*t*) 0.000 (*df*) 680	0.00 ± 0.0 Hz vs. 0.00 ± 0.0 Hz	8 vs. 14 cells
		0 pA: LIII vs. LX	>0.9999	(*t*) 0.000 (*df*) 680	0.00 ± 0.0 Hz vs. 0.00 ± 0.0 Hz	8 vs. 14 cells
		0 pA: LIII vs. LX	>0.9999	(*t*) 0.000 (*df*) 680	0.00 ± 0.0 Hz vs. 0.00 ± 0.0 Hz	8 vs. 14 cells
		40 pA: LIII vs. LX	>0.9999	(*t*) 0.000 (*df*) 680	0.00 ± 0.0 Hz vs. 0.00 ± 0.0 Hz	8 vs. 14 cells
		80 pA: LIII vs. LX	0.9056	(*t*) 0.119 (*df*) 680	0.94 ± 0.9 Hz vs. 1.82 ± 1.4 Hz	8 vs. 14 cells
		120 pA: LIII vs. LX	0.7201	(*t*) 0.358 (*df*) 680	14.56 ± 7.2 Hz vs. 11.89 ± 4.0 Hz	8 vs. 14 cells
		160 pA: LIII vs. LX	0.2843	(*t*) 1.072 (*df*) 680	32.69 ± 11.5 Hz vs. 24.71 ± 5.8 Hz	8 vs. 14 cells
		200 pA: LIII vs. LX	0.1556	(*t*) 1.422 (*df*) 680	50.38 ± 13.4 Hz vs. 39.79 ± 6.8 Hz	8 vs. 14 cells
		240 pA: LIII vs. LX	0.2014	(*t*) 1.279 (*df*) 680	66.31 ± 14.2 Hz vs. 56.79 ± 6.4 Hz	8 vs. 14 cells
		280 pA: LIII vs. LX	0.0787	(*t*) 1.761 (*df*) 680	81.25 ± 15.5 Hz vs. 68.13 ± 6.2 Hz	8 vs. 14 cells
		320 pA: LIII vs. LX	**0.0355**	(*t*) 2.107 (*df*) 680	91.00 ± 16.4 Hz vs. 75.30 ± 5.9 Hz	8 vs. 14 cells
		280 pA: LIII vs. LX	0.0969	(*t*) 1.663 (*df*) 680	81.44 ± 15.5 Hz vs. 69.05 ± 5.9 Hz	8 vs. 14 cells
		240 pA: LIII vs. LX	0.3302	(*t*) 0.975 (*df*) 680	67.81 ± 14.5 Hz vs. 60.55 ± 5.5 Hz	8 vs. 14 cells
		200 pA: LIII vs. LX	0.8163	(*t*) 0.233 (*df*) 680	53.13 ± 14.2 Hz vs. 51.39 ± 4.3 Hz	8 vs. 14 cells
		160 pA: LIII vs. LX	0.5443	(*t*) 0.607 (*df*) 680	37.00 ± 12.6 Hz vs. 41.52 ± 2.3 Hz	8 vs. 14 cells
		120 pA: LIII vs. LX	0.2935	(*t*) 1.051 (*df*) 680	22.75 ± 10.6 Hz vs. 30.58 ± 0.4 Hz	8 vs. 14 cells
		80 pA: LIII vs. LX	0.2056	(*t*) 1.267 (*df*) 680	10.75 ± 7.4 Hz vs. 20.19 ± 0.2 Hz	8 vs. 14 cells
		40 pA: LIII vs. LX	0.4561	(*t*) 0.746 (*df*) 680	2.38 ± 2.4 Hz vs. 7.93 ± 0.0 Hz	8 vs. 14 cells
		0 pA: LIII vs. LX	0.9236	(*t*) 0.096 (*df*) 680	0.00 ± 0.0 Hz vs. 0.71 ± 0.0 Hz	8 vs. 14 cells
		0 pA: LIII vs. LX	0.9771	(*t*) 0.029 (*df*) 680	0.00 ± 0.0 Hz vs. 0.21 ± 0.0 Hz	8 vs. 14 cells
		0 pA: LIII vs. LX	>0.9999	(*t*) 0.000 (*df*) 680	0.00 ± 0.0 Hz vs. 0.00 ± 0.0 Hz	8 vs. 14 cells
		0 pA: LIII vs. LX	>0.9999	(*t*) 0.000 (*df*) 680	0.00 ± 0.0 Hz vs. 0.00 ± 0.0 Hz	8 vs. 14 cells
		0 pA: LIII vs. LX	>0.9999	(*t*) 0.000 (*df*) 680	0.00 ± 0.0 Hz vs. 0.00 ± 0.0 Hz	8 vs. 14 cells
		0 pA: LIII vs. LX	>0.9999	(*t*) 0.000 (*df*) 680	0.00 ± 0.0 Hz vs. 0.00 ± 0.0 Hz	8 vs. 14 cells
		0 pA: LIII vs. LX	>0.9999	(*t*) 0.000 (*df*) 680	0.00 ± 0.0 Hz vs. 0.00 ± 0.0 Hz	8 vs. 14 cells
		0 pA: LIII vs. LX	>0.9999	(*t*) 0.000 (*df*) 680	0.00 ± 0.0 Hz vs. 0.00 ± 0.0 Hz	8 vs. 14 cells
		0 pA: LIII vs. LX	>0.9999	(*t*) 0.000 (*df*) 680	0.00 ± 0.0 Hz vs. 0.00 ± 0.0 Hz	8 vs. 14 cells
Figure 6D2	Two-way ANOVA	0 pA: LIII↓ vs. LIII↑	>0.9999	(*t*) 0.000 (*df*) 112	0.00 ± 0.0 Hz vs. 0.00 ± 0.0 Hz	8 cells
		0 pA: LIII↓ vs. LIII↑	>0.9999	(*t*) 0.000 (*df*) 112	0.00 ± 0.0 Hz vs. 0.00 ± 0.0 Hz	
		0 pA: LIII↓ vs. LIII↑	>0.9999	(*t*) 0.000 (*df*) 112	0.00 ± 0.0 Hz vs. 0.00 ± 0.0 Hz	
		0 pA: LIII↓ vs. LIII↑	>0.9999	(*t*) 0.000 (*df*) 112	0.00 ± 0.0 Hz vs. 0.00 ± 0.0 Hz	
		0 pA: LIII↓ vs. LIII↑	>0.9999	(*t*) 0.000 (*df*) 112	0.00 ± 0.0 Hz vs. 0.00 ± 0.0 Hz	
		0 pA: LIII↓ vs. LIII↑	>0.9999	(*t*) 0.000 (*df*) 112	0.00 ± 0.0 Hz vs. 0.00 ± 0.0 Hz	
		0 pA: LIII↓ vs. LIII↑	>0.9999	(*t*) 0.000 (*df*) 112	0.00 ± 0.0 Hz vs. 0.00 ± 0.0 Hz	
		0 pA: LIII↓ vs. LIII↑	>0.9999	(*t*) 0.000 (*df*) 112	0.00 ± 0.0 Hz vs. 0.00 ± 0.0 Hz	
		0 pA: LIII↓ vs. LIII↑	>0.9999	(*t*) 0.000 (*df*) 112	0.00 ± 0.0 Hz vs. 0.00 ± 0.0 Hz	
		40 pA: LIII↓ vs. LIII↑	0.3496	(*t*) 0.939 (*df*) 112	0.00 ± 0.0 Hz vs. 2.38 ± 2.4 Hz	
		80 pA: LIII↓ vs. LIII↑	**0.0002**	(*t*) 3.881 (*df*) 112	0.94 ± 0.9 Hz vs. 10.75 ± 7.4 Hz	

		120 pA: LIII↓ vs. LIII↑	**<u>0.0016</u>**	(*t*) 3.238 (*df*) 112	14.56 ± 7.2 Hz vs. 22.75 ± 10.6 Hz	
		160 pA: LIII↓ vs. LIII↑	0.0909	(*t*) 1.705 (*df*) 112	32.69 ± 11.5 Hz vs. 37.00 ± 12.6 Hz	
		200 pA: LIII↓ vs. LIII↑	0.2791	(*t*) 1.088 (*df*) 112	50.38 ± 13.4 Hz vs. 53.13 ± 14.2 Hz	
		240 pA: LIII↓ vs. LIII↑	0.5542	(*t*) 0.593 (*df*) 112	66.31 ± 14.2 Hz vs. 67.81 ± 14.5 Hz	
		280 pA: LIII↓ vs. LIII↑	0.9410	(*t*) 0.074 (*df*) 112	81.25 ± 15.5 Hz vs. 81.44 ± 15.5 Hz	
		320 pA			91.00 ± 16.4 Hz	
Figure 6D3	Two-way ANOVA	0 pA: LX↓ vs. LX↑	>0.9999	(*t*) 0.000 (*df*) 208	0.00 ± 0.0 Hz vs. 0.00 ± 0.0 Hz	14 cells
		0 pA: LX↓ vs. LX↑	>0.9999	(*t*) 0.000 (*df*) 208	0.00 ± 0.0 Hz vs. 0.00 ± 0.0 Hz	
		0 pA: LX↓ vs. LX↑	>0.9999	(*t*) 0.000 (*df*) 208	0.00 ± 0.0 Hz vs. 0.00 ± 0.0 Hz	
		0 pA: LX↓ vs. LX↑	>0.9999	(*t*) 0.000 (*df*) 208	0.00 ± 0.0 Hz vs. 0.00 ± 0.0 Hz	
		0 pA: LX↓ vs. LX↑	>0.9999	(*t*) 0.000 (*df*) 208	0.00 ± 0.0 Hz vs. 0.00 ± 0.0 Hz	
		0 pA: LX↓ vs. LX↑	>0.9999	(*t*) 0.000 (*df*) 208	0.00 ± 0.0 Hz vs. 0.00 ± 0.0 Hz	
		0 pA: LX↓ vs. LX↑	>0.9999	(*t*) 0.000 (*df*) 208	0.00 ± 0.0 Hz vs. 0.00 ± 0.0 Hz	
		0 pA: LX↓ vs. LX↑	0.9341	(*t*) 0.083 (*df*) 208	0.00 ± 0.0 Hz vs. 0.21 ± 0.2 Hz	
		0 pA: LX↓ vs. LX↑	0.7828	(*t*) 0.276 (*df*) 208	0.00 ± 0.0 Hz vs. 0.71 ± 0.4 Hz	
		40 pA: LX↓ vs. LX↑	**<u>0.0025</u>**	(*t*) 3.064 (*df*) 208	0.00 ± 0.0 Hz vs. 7.93 ± 2.3 Hz	
		80 pA: LX↓ vs. LX↑	**<u><0.0001</u>**	(*t*) 7.098 (*df*) 208	1.82 ± 1.4 Hz vs. 20.19 ± 4.3 Hz	
		120 pA: LX↓ vs. LX↑	**<u><0.0001</u>**	(*t*) 7.222 (*df*) 208	11.89 ± 4.0 Hz vs. 30.58 ± 5.5 Hz	
		160 pA: LX↓ vs. LX↑	**<u><0.0001</u>**	(*t*) 6.498 (*df*) 208	24.71 ± 5.8 Hz vs. 41.52 ± 5.9 Hz	
		200 pA: LX↓ vs. LX↑	**<u><0.0001</u>**	(*t*) 4.486 (*df*) 208	39.79 ± 6.8 Hz vs. 51.39 ± 5.9 Hz	
		240 pA: LX↓ vs. LX↑	0.1468	(*t*) 1.456 (*df*) 208	56.79 ± 6.0 Hz vs. 60.55 ± 6.2 Hz	
		280 pA: LX↓ vs. LX↑	0.7225	(*t*) 0.356 (*df*) 208	68.13 ± 6.3 Hz vs. 69.05 ± 6.4 Hz	
		320 pA			75.30 ± 6.8 Hz	

Table S7. Statistical data for in vitro whole cell patch electrophysiology in the adult mouse (P>30), tail-current analysis. Data for *in vitro* extracellular recordings in the adult mouse (P>30), for propofol effect analysis, and whole cell for action potential properties with propofol. Descriptive values are represented as ± SEM. Abbreviations: LIII = Lobule III, LX = Lobule X, *df* = degrees of freedom, pA = picoampere, Hz = hertz, mV = millivolt, Vm = membrane voltage, Ω = Ohm, msec = milliseconds, aCSF = artificial cerebral spinal fluid aCSF+P = aCSF with propofol added, aCSF+PC = aCSF with propofol and CGP added, aCSF+Z = aCSF with propofol and zatebradine added.

Related Figure	Test Applied	Group	*p*-Value	Population-Value	Descriptive Values	Population Size
Figure 7B1	Mixed-effects analysis	Time x Column factor	**<0.0001**	F(16, 416)		
		-130 pA: LIII vs. LX	**<0.0001**	(*t*) 4.324 (*df*) 445	193.9 ± 12 pA vs. 144.4 ± 11 pA	16 vs. 15 cells
		-125 pA: LIII vs. LX	**<0.0001**	(*t*) 4.773 (*df*) 445	194.7 ± 13 pA vs. 136.8 ± 11 pA	12 vs. 13 cells
		-120 pA: LIII vs. LX	**<0.0001**	(*t*) 4.843 (*df*) 445	187.4 ± 14 pA vs. 131.9 ± 9 pA	16 vs. 15 cells
		-115 pA: LIII vs. LX	**<0.0001**	(*t*) 5.110 (*df*) 445	187.3 ± 11 pA vs. 125.3 ± 11 pA	12 vs. 13 cells
		-110 pA: LIII vs. LX	**<0.0001**	(*t*) 5.110 (*df*) 445	175.9 ± 10 pA vs. 117.3 ± 9 pA	16 vs. 15 cells
		-105 pA: LIII vs. LX	**<0.0001**	(*t*) 4.684 (*df*) 445	172.1 ± 9 pA vs. 115.2 ± 9 pA	12 vs. 13 cells
		-100 pA: LIII vs. LX	**<0.0001**	(*t*) 4.797 (*df*) 445	160.6 ± 9 pA vs. 105.6 ± 8 pA	16 vs. 15 cells
		-95 pA: LIII vs. LX	**<0.0001**	(*t*) 4.914 (*df*) 445	158.8 ± 7 pA vs. 99.1 ± 9 pA	12 vs. 13 cells
		-90 pA: LIII vs. LX	**<0.0001**	(*t*) 4.278 (*df*) 445	139.1 ± 8 pA vs. 90.0 ± 7 pA	16 vs. 15 cells
		-85 pA: LIII vs. LX	**0.0004**	(*t*) 3.596 (*df*) 445	123.8 ± 6 pA vs. 79.9 ± 8 pA	12 vs. 13 cells
		-80 pA: LIII vs. LX	**0.0011**	(*t*) 3.285 (*df*) 445	105.1 ± 7 pA vs. 67.4 ± 7 pA	16 vs. 15 cells
		-75 pA: LIII vs. LX	**0.0048**	(*t*) 2.836 (*df*) 445	89.5 ± 8 pA vs. 54.7 ± 6 pA	12 vs. 13 cells
		-70 pA: LIII vs. LX	**0.0220**	(*t*) 2.299 (*df*) 445	66.5 ± 6 pA vs. 40.2 ± 5 pA	16 vs. 15 cells
		-65 pA: LIII vs. LX	0.1641	(*t*) 1.394 (*df*) 445	45.5 ± 7 pA vs. 28.0 ± 3 pA	12 vs. 13 cells
		-60 pA: LIII vs. LX	0.2438	(*t*) 1.167 (*df*) 445	30.6 ± 3 pA vs. 17.2 ± 2 pA	16 vs. 15 cells
		-55 pA: LIII vs. LX	0.8729	(*t*) 0.160 (*df*) 445	14.1 ± 2 pA vs. 11.3 ± 2 pA	12 vs. 13 cells
		-50 pA: LIII vs. LX	>0.9999	(*t*) 0.000 (*df*) 445	0.0 ± 0 pA vs. 0.0 ± 0 pA	16 vs. 15 cells
Figure 7B2	Mixed-effects analysis	Time x Column factor	**<0.0001**	F(16, 422)		
		-130 pA: LIII vs. LX	**0.0013**	(*t*) 3.241 (*df*) 451	123.5 ± 12 pA vs. 93.5 ± 9 pA	16 vs. 15 cells
		-125 pA: LIII vs. LX	**0.0018**	(*t*) 3.144 (*df*) 451	115.1 ± 13 pA vs. 90.9 ± 8 pA	12 vs. 13 cells
		-120 pA: LIII vs. LX	**0.0004**	(*t*) 3.544 (*df*) 451	115.8 ± 11 pA vs. 81.6 ± 6 pA	16 vs. 15 cells
		-115 pA: LIII vs. LX	**0.0003**	(*t*) 3.606 (*df*) 451	107.7 ± 12 pA vs. 77.0 ± 8 pA	12 vs. 13 cells
		-110 pA: LIII vs. LX	**0.0003**	(*t*) 3.686 (*df*) 451	104.2 ± 10 pA vs. 68.6 ± 7 pA	16 vs. 15 cells
		-105 pA: LIII vs. LX	**0.0036**	(*t*) 2.925 (*df*) 451	94.7 ± 11 pA vs. 70.8 ± 7 pA	12 vs. 13 cells
		-100 pA: LIII vs. LX	**0.0053**	(*t*) 2.804 (*df*) 451	89.5 ± 9 pA vs. 62.4 ± 7 pA	16 vs. 15 cells
		-95 pA: LIII vs. LX	**0.0136**	(*t*) 2.479 (*df*) 451	75.5 ± 9 pA vs. 56.2 ± 7 pA	12 vs. 13 cells
		-90 pA: LIII vs. LX	**0.0356**	(*t*) 2.108 (*df*) 451	69.8 ± 8 pA vs. 49.4 ± 6 pA	16 vs. 15 cells
		-85 pA: LIII vs. LX	**0.0450**	(*t*) 2.010 (*df*) 451	55.5 ± 7 pA vs. 40.8 ± 5 pA	12 vs. 13 cells
		-80 pA: LIII vs. LX	0.2164	(*t*) 1.238 (*df*) 451	46.4 ± 6 pA vs. 34.5 ± 5 pA	16 vs. 15 cells
		-75 pA: LIII vs. LX	0.1599	(*t*) 1.408 (*df*) 451	33.8 ± 5 pA vs. 25.1 ± 5 pA	12 vs. 13 cells
		-70 pA: LIII vs. LX	0.4581	(*t*) 0.743 (*df*) 451	26.3 ± 3 pA vs. 19.2 ± 3 pA	16 vs. 15 cells
		-65 pA: LIII vs. LX	0.4646	(*t*) 0.732 (*df*) 451	17.1 ± 2 pA vs. 15.1 ± 3 pA	12 vs. 13 cells
		-60 pA: LIII vs. LX	0.6434	(*t*) 0.463 (*df*) 451	13.9 ± 2 pA vs. 9.4 ± 2 pA	16 vs. 15 cells
		-55 pA: LIII vs. LX	0.3855	(*t*) 0.869 (*df*) 451	10.2 ± 2 pA vs. 6.9 ± 2 pA	12 vs. 13 cells
		-50 pA: LIII vs. LX	>0.9999	(*t*) 0.000 (*df*) 451	0.0 ± 0 pA vs. 0.0 ± 0 pA	16 vs. 15 cells
Figure 7B3	Mixed-effects analysis	Time x Column factor	**<0.0210**	F(16, 422)		
		-130 pA: LIII vs. LX	0.0778	(*t*) 1.768 (*df*) 451	69.8 ± 9 pA vs. 50.9 ± 10 pA	16 vs. 15 cells
		-125 pA: LIII vs. LX	0.2572	(*t*) 1.134 (*df*) 451	62.8 ± 15 pA vs. 45.9 ± 9 pA	12 vs. 13 cells
		-120 pA: LIII vs. LX	**0.0362**	(*t*) 2.101 (*df*) 451	71.5 ± 8 pA vs. 50.3 ± 7 pA	16 vs. 15 cells
		-115 pA: LIII vs. LX	0.2040	(*t*) 1.272 (*df*) 451	65.2 ± 14 pA vs. 48.3 ± 9 pA	12 vs. 13 cells
		-110 pA: LIII vs. LX	**0.0238**	(*t*) 2.268 (*df*) 451	71.7 ± 7 pA vs. 48.7 ± 7 pA	16 vs. 15 cells
		-105 pA: LIII vs. LX	0.1231	(*t*) 1.545 (*df*) 451	64.2 ± 13 pA vs. 44.4 ± 7 pA	12 vs. 13 cells
		-100 pA: LIII vs. LX	**0.0061**	(*t*) 2.756 (*df*) 451	71.1 ± 7 pA vs. 43.2 ± 6 pA	16 vs. 15 cells
		-95 pA: LIII vs. LX	**0.0205**	(*t*) 2.324 (*df*) 451	71.0 ± 12 pA vs. 43.0 ± 8 pA	12 vs. 13 cells
		-90 pA: LIII vs. LX	**0.0048**	(*t*) 2.833 (*df*) 451	69.2 ± 6 pA vs. 40.6 ± 6 pA	16 vs. 15 cells
		-85 pA: LIII vs. LX	0.1267	(*t*) 1.530 (*df*) 451	58.8 ± 11 pA vs. 39.1 ± 6 pA	12 vs. 13 cells
		-80 pA: LIII vs. LX	**0.0114**	(*t*) 2.539 (*df*) 451	58.6 ± 6 pA vs. 33.0 ± 6 pA	16 vs. 15 cells
		-75 pA: LIII vs. LX	0.1368	(*t*) 1.490 (*df*) 451	48.8 ± 9 pA vs. 29.6 ± 5 pA	12 vs. 13 cells
		-70 pA: LIII vs. LX	0.0587	(*t*) 1.895 (*df*) 451	40.2 ± 5 pA vs. 21.0 ± 4 pA	16 vs. 15 cells
		-65 pA: LIII vs. LX	0.4142	(*t*) 0.817 (*df*) 451	25.0 ± 6 pA vs. 12.8 ± 3 pA	12 vs. 13 cells
		-60 pA: LIII vs. LX	0.3795	(*t*) 0.880 (*df*) 451	16.6 ± 3 pA vs. 7.7 ± 2 pA	16 vs. 15 cells
		-55 pA: LIII vs. LX	0.6312	(*t*) 0.480 (*df*) 451	2.8 ± 3 pA vs. 4.4 ± 1 pA	12 vs. 13 cells
		-50 pA: LIII vs. LX	>0.9999	(*t*) 0.000 (*df*) 451	0.0 ± 0 pA vs. 0.0 ± 0 pA	16 vs. 15 cells

Figure 7C2	Kruskal–Wallis test	aCSF+P: LIII vs LX	0.0565	(*Z*) 1.907	78.88 ± 6.0 Hz vs. 60.48 ± 5.5 Hz	28 vs. 27 cells
		aCSF+PC: LIII vs LX	**0.0009**	(*Z*) 3.330	81.90 ± 7.3 Hz vs. 43.99 ± 6.6 Hz	11 vs. 11 cells
		LIII: aCSF+P vs. aCSF+PC	0.5824	(*Z*) 0.550	78.88 ± 6.0 Hz vs. 81.90 ± 7.3 Hz	28 vs. 11 cells
		LX: aCSF+P vs. aCSF+PC	**0.0471**	(*Z*) 1.985	60.48 ± 5.5 Hz vs. 43.99 ± 6.6 Hz	27 vs. 11 cells
Figure 7C3	Kruskal–Wallis test	aCSF+P: LIII vs LX	**0.0011**	(*Z*) 3.269	0.118 ± 0.02 vs. 0.093 ± 0.02	28 vs. 27 cells
		aCSF+PC: LIII vs LX	**0.0006**	(*Z*) 3.411	0.104 ± 0.02 vs. 0.040 ± 0.00	11 vs. 11 cells
		LIII: aCSF+P vs. aCSF+PC	0.8958	(*Z*) 0.131	0.118 ± 0.02 vs. 0.104 ± 0.02	28 vs. 11 cells
		LX: aCSF+P vs. aCSF+PC	0.0833	(*Z*) 1.732	0.093 ± 0.02 vs. 0.040 ± 0.00	27 vs. 11 cells
Figure 7D	Unpaired *t*-test	LIII vs. LX	0.9912	(*t*) 0.011 (*df*) 18	-43.66 ± 1.6 Vm vs. -43.64 ± 1.2 Vm	9 vs. 11 cells
Figure 7E	Unpaired *t*-test	LIII vs. LX	0.1387	(*t*) 1.549 (*df*) 18	85.60 ± 1.9 mV vs. 92.16 ± 3.5 mV	9 vs. 11 cells
Figure 7F1	Welch's *t*-test	LIII vs. LX	**0.0023**	(*t*) 3.772 (*df*) 13	0.156 ± 0.006 msec vs. 0.211 ± 0.014 msec	9 vs. 11 cells
Figure 7F2	Unpaired *t*-test	LIII vs. LX	0.5847	(*t*) 0.557 (*df*) 18	0.133 ± 0.006 msec vs. 0.138 ± 0.006 msec	9 vs. 11 cells
Figure 7F3	Welch's *t*-test	LIII vs. LX	**<0.0001**	(*t*) 6.379 (*df*) 11	0.116 ± 0.003 msec vs. 0.229 ± 0.190 msec	9 vs. 11 cells
Figure 7 S1A1	Welch's *t*-test	LIII vs. LX	**0.0127**	(*t*) 2.785 (*df*) 17	84.40 ± 5.6 Ω vs. 134.3 ± 17.0 Ω	16 vs. 15 cells
Figure 7 S1A2	Welch's *t*-test	LIII vs. LX	**0.0118**	(*t*) 2.824 (*df*) 17	68.67 ± 5.4 Ω vs. 118.7 ± 16.9 Ω	16 vs. 15 cells
Figure 7 S1A3	Unpaired *t*-test	LIII vs. LX	0.8988	(*t*) 0.128 (*df*) 29	15.73 ± 0.8 Ω vs. 15.58 ± 0.8 Ω	16 vs. 15 cells
Figure 7 S1B1	Mixed-effects analysis	LIII: aCSF vs. aCSF+P	**<0.0001**	F(16, 182)		
		LIII: aCSF vs. aCSF+Z	**<0.0001**	F(16, 21)		
		LIII: aCSF+P vs. aCSF+Z	**<0.0001**	F(16, 27)		
Figure 7 S1B2	Mixed-effects analysis	LX: aCSF vs. aCSF+P	**<0.0001**	F(16, 192)		
		LX: aCSF vs. aCSF+Z	**<0.0001**	F(16, 16)		
		LX: aCSF+P vs. aCSF+Z	**<0.0001**	F(16, 18)		
Figure 7 S1B3	Two-way ANOVA	LIII aCSF+Z vs. LX aCSF+Z	0.7754	F(16, 80)		
Figure 7 S1D1	Unpaired *t*-test	LIII vs. LX	**0.0358**	(*t*) 2.252 (*df*) 20	64.95 ± 3.9 Ω vs. 84.07 ± 6.5 Ω	9 vs. 13 cells
Figure 7 S1D2	Mann–Whitney U test	LIII vs. LX	**0.0364**	(*U*) 27	49.54 ± 3.8 Ω vs. 68.56 ± 6.4 Ω	9 vs. 13 cells
Figure 7 S1D3	Unpaired *t*-test	LIII vs. LX	0.9232	(*t*) 0.098 (*df*) 20	15.41 ± 0.7 Ω vs. 15.51 ± 0.7 Ω	9 vs. 13 cells
Figure 7 S1E	Unpaired *t*-test	LIII vs. LX	0.8031	(*t*) 0.2531 (*df*) 18	76.00 ± 12.5 pA vs. 69.91 ± 19.2 pA	9 vs. 11 cells
Figure 7 S1F	Unpaired *t*-test	LIII vs. LX	**0.0585**	(*t*) 2.020 (*df*) 18	34.90 ± 1.6 Vm vs. 42.13 ± 3.0 Vm	9 vs. 11 cells
